# Adding α,α-disubstituted and β-linked monomers to the genetic code of an organism

**DOI:** 10.1038/s41586-023-06897-6

**Published:** 2024-01-10

**Authors:** Daniel L. Dunkelmann, Carlos Piedrafita, Alexandre Dickson, Kim C. Liu, Thomas S. Elliott, Marc Fiedler, Dom Bellini, Andrew Zhou, Daniele Cervettini, Jason W. Chin

**Affiliations:** Medical Research Council Laboratory of Molecular Biology, Cambridge, UK

**Keywords:** Synthetic biology, Synthetic biology, tRNAs

## Abstract

The genetic code of living cells has been reprogrammed to enable the site-specific incorporation of hundreds of non-canonical amino acids into proteins, and the encoded synthesis of non-canonical polymers and macrocyclic peptides and depsipeptides^1–3^. Current methods for engineering orthogonal aminoacyl-tRNA synthetases to acylate new monomers, as required for the expansion and reprogramming of the genetic code, rely on translational readouts and therefore require the monomers to be ribosomal substrates^4–6^. Orthogonal synthetases cannot be evolved to acylate orthogonal tRNAs with non-canonical monomers (ncMs) that are poor ribosomal substrates, and ribosomes cannot be evolved to polymerize ncMs that cannot be acylated onto orthogonal tRNAs—this co-dependence creates an evolutionary deadlock that has essentially restricted the scope of translation in living cells to α-l-amino acids and closely related hydroxy acids. Here we break this deadlock by developing tRNA display, which enables direct, rapid and scalable selection for orthogonal synthetases that selectively acylate their cognate orthogonal tRNAs with ncMs in *Escherichia coli*, independent of whether the ncMs are ribosomal substrates. Using tRNA display, we directly select orthogonal synthetases that specifically acylate their cognate orthogonal tRNA with eight non-canonical amino acids and eight ncMs, including several β-amino acids, α,α-disubstituted-amino acids and β-hydroxy acids. We build on these advances to demonstrate the genetically encoded, site-specific cellular incorporation of β-amino acids and α,α-disubstituted amino acids into a protein, and thereby expand the chemical scope of the genetic code to new classes of monomers.

## Abstract (web summary)

tRNA display enables the direct selection of orthogonal aminoacyl-tRNA synthetases that acylate orthogonal tRNAs with non-canonical monomers, enabling in vivo synthesis of proteins that include these monomers and expanding the repertoire of the genetic code.

## Main

The genetic code of living cells has been reprogrammed to enable the site-specific incorporation of hundreds of non-canonical amino acids (ncAAs) into proteins^1,7,8^, and the encoded synthesis of non-canonical polymers and macrocyclic peptides and depsipeptides^2,3,9^. Despite remarkable progress, the monomers that can be site-specifically incorporated into proteins in cells have been essentially limited to α-l-amino acids with variant side chains, and closely related hydroxy acids. Although a wider range of monomers have been incorporated in in vitro translation reactions^10–14^ primarily into short peptides, these in vitro approaches cannot be extended to living cells. Under starvation conditions, the permissivity of an endogenous phenylalanyl-tRNA synthetase to a β-amino acid has been exploited for its low-level incorporation, in competition with phenylalanine, at all phenylalanine codons^15^; this approach generates a mixture of cellular proteins, is incompatible with quantitative, site-specific incorporation at a single position in response to a single codon, and is therefore fundamentally incompatible with reprogramming the genetic code.

**Extended Data Fig. 1:**
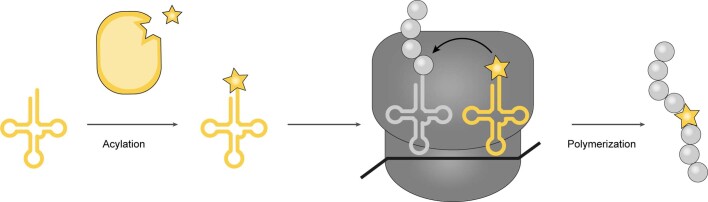
Encoded cellular incorporation of non-canonical monomers into proteins and into non-canonical polymers requires both tRNA acylation and ribosomal polymerization. The encoded, site specific, incorporation of a non-canonical monomer (ncM, yellow star) via cellular translation requires both the acylation of an orthogonal tRNA with the ncM by an orthogonal synthetase, and ribosomal polymerization of the ncM into a polymer chain; arrow indicates peptide bond formation between A-site monomer and P-site nascent chain. Current methods for engineering aminoacyl-tRNA synthetases that acylate new monomers rely on translational readouts and therefore require the monomers to be ribosomal substrates. For ncMs that are poor ribosomal substrates this co-dependence creates an evolutionary deadlock in cells; an orthogonal synthetase cannot be evolved to acylate an orthogonal tRNA with ncMs that are poor ribosomal substrates, and ribosomes cannot be evolved to polymerize ncMs that cannot be acylated onto orthogonal tRNAs. To break this deadlock, we develop direct selections for orthogonal synthetases to aminoacylate their cognate orthogonal tRNAs with ncMs, independent of whether the ncMs are ribosomal substrates.

The encoded, site-specific, incorporation of ncMs via cellular translation requires the creation of orthogonal aminoacyl-tRNA synthetase (aaRS)–orthogonal tRNA pairs. The orthogonal synthetase recognizes a ncM but not the canonical amino acids present in the cell, and selectively transfers the ncM onto its cognate orthogonal tRNA, which is targeted to a blank codon (most commonly the amber stop codon). The ncM, once loaded onto the orthogonal tRNA, must also be a substrate for ribosomal polymerization (Extended Data Fig. 1). Current methods for engineering aaRSs that selectively acylate new monomers onto their cognate tRNAs rely on translational readouts^4,6,16^ and therefore require the monomers to be ribosomal substrates for incorporation, often at specific sites in proteins. Since many ncMs of interest are poor ribosomal substrates^13,15,17–21^, this creates an evolutionary deadlock in cells; an orthogonal synthetase cannot be evolved to selectively acylate an orthogonal tRNA with ncMs that are poor ribosomal substrates, and ribosomes cannot be evolved to polymerize ncMs that cannot be selectively acylated onto orthogonal tRNAs. We realized that this deadlock might be broken by developing direct selections for orthogonal synthetases that selectively acylate their cognate orthogonal tRNAs with ncMs, independent of whether the ncMs are ribosomal substrates.

We previously described tRNA extension (tREX), a rapid method to determine the aminoacylation status of user-defined tRNAs from cells^22^. Here we develop derivatives of tREX that enable specific acylated tRNAs, isolated from cells, to be fluorescently labelled (fluoro-tREX) or captured (bio–tREX). We create strategies for producing split ${{\rm{t}}{\rm{R}}{\rm{N}}{\rm{A}}}^{{\rm{P}}{\rm{y}}{\rm{l}}}$ (derived from *Methanosarcina mazei* pyrrolysl tRNA_CUA_), which contains new 5′ and 3′ ends at the anticodon.

We connect the genotype responsible for acylation to the acylation itself by fusing the gene for *M. mazei* pyrrolysyl-tRNA synthetase (hereafter referred to as PylRS) to a split tRNA^Pyl^ (stRNA^Pyl^) gene, creating stmRNA^Pyl^ genes that encode stmRNA^Pyl^, in which the 3′ half of the stRNA^Pyl^ is fused to the PylRS mRNA. We demonstrate that we can selectively enrich—by more than 300-fold—stmRNAs encoding active PylRS variants with respect to attenuated activity variants, using bio–mREX (a variation of bio–tREX applied to the stmRNA).

Bio–mREX forms the basis of tRNA display, a method for discovering synthetases that acylate their cognate tRNAs with ncMs, independent of whether the ncMs are substrates for ribosomal translation. We use tRNA display to select orthogonal aaRSs that specifically acylate their cognate, orthogonal tRNAs with several β-amino acids, an α,α-disubstituted amino acid and a β-hydroxy acid. Moreover, we build on our advance to enable the site-specific co-translational incorporation of selected β-amino acids and α,α-disubstituted amino acids into a recombinant protein produced in *E. coli*; we produce milligrams of ncM-containing protein per litre of culture, and solve the structure of a β-amino acid-containing protein.

## Detection and isolation of acylated tRNAs

**Fig. 1:**
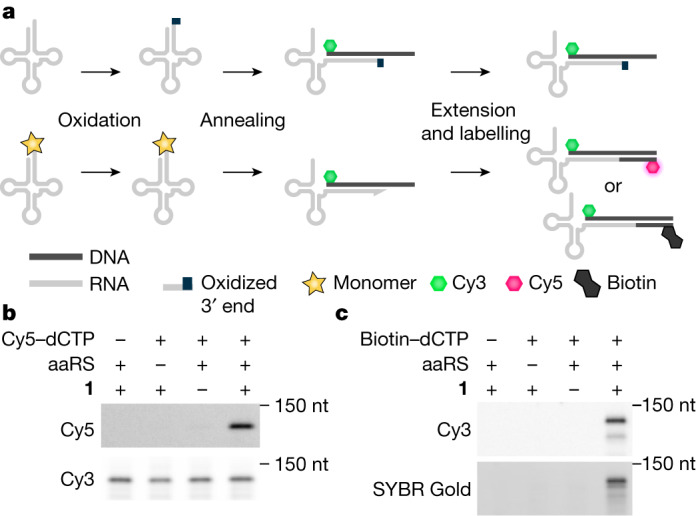
Acylation-dependent tRNA extension enables the sensitive detection and isolation of acylated tRNAs. **a**, Schematic of fluoro-tREX and bio–tREX protocols. tRNAs are isolated from cells, and the diol functionality of the 3′ ribose on non-acylated tRNAs is oxidized to the dialdehyde. The acyl group of charged tRNAs protects the diol functionality of the 3′ ribose and prevents oxidation to a dialdehyde. A Cy3-labelled DNA probe complementary to the 3′ end of a target tRNA is annealed, and target tRNAs that were acylated are extended upon addition of Klenow fragment exo− and modified nucleotides. For fluoro-tREX, Cy5-labelled nucleotides are incorporated. Acylated tRNAs lead to a Cy5 and Cy3 signal, whereas non-acylated tRNAs only give a Cy3 signal. For bio–tREX, biotinylated nucleotides are incorporated and the tRNAs that were acylated are purified using streptavidin beads and can be visualized by SYBR gold staining following gel electrophoresis. **b**, Fluoro-tREX detected the acylation of ${{\rm{tRNA}}}_{{\rm{C}}{\rm{U}}{\rm{A}}}^{{\rm{P}}{\rm{y}}{\rm{l}}}$ in the presence of PylRS and BocK (**1**). Cells expressing ${{\rm{tRNA}}}_{{\rm{C}}{\rm{U}}{\rm{A}}}^{{\rm{P}}{\rm{y}}{\rm{l}}}$ were grown in the presence and absence of PylRS and BocK (**1**). **c**, Bio–tREX enables the selective isolation of previously acylated tRNAs. Cells harbouring ${{\rm{tRNA}}}_{{\rm{C}}{\rm{U}}{\rm{A}}}^{{\rm{P}}{\rm{y}}{\rm{l}}}$ were grown in the presence and absence of PylRS and BocK (**1**). Isolation of the tRNA and associated probe was visualized by SYBR Gold staining for RNA and Cy3 fluorescence for the probe. Experiments in **b**,**c** were repeated three times with similar results. For full, uncropped gels for all figures see Supplementary Fig. 1.

We demonstrated that we could determine the aminoacylation status of a specific tRNA isolated from cells by periodate oxidation followed by selective, probe-mediated extension of non-oxidized tRNAs with nucleotide derivatives bearing a fluorophore (Cy5) or biotin (Fig. 1a).

We isolated tRNAs from cells expressing ${{\rm{tRNA}}}_{{\rm{C}}{\rm{U}}{\rm{A}}}^{{\rm{P}}{\rm{y}}{\rm{l}}}$ in the presence and absence of PylRS and (N^ε^-((*tert*-butoxy)carbonyl)-l-lysine (BocK, **1**)), a known and efficient substrate of PylRS. We oxidized the isolated tRNAs with sodium periodate and annealed a DNA probe, containing a 3′ Cy3 and a 5′ poly-G stretch, to the 3′ end of tRNA^Pyl^. We extended the free 3′ end of ${{\rm{tRNA}}}_{{\rm{C}}{\rm{U}}{\rm{A}}}^{{\rm{P}}{\rm{y}}{\rm{l}}}$, resulting from aminoacylation-mediated protection from periodate oxidation, using Klenow fragment exo− and a dNTP mix in which dCTP was replaced with Cy5-labelled dCTP; the free 3′ end of the tRNA acts as an RNA primer for Klenow fragment exo−, which uses the dNTPs to produce the reverse complement of the annealed DNA probe. We visualized the fluorescent signals following gel electrophoresis, specifically detecting a strong Cy5-labelled band corresponding to extended ${{\rm{tRNA}}}_{{\rm{C}}{\rm{U}}{\rm{A}}}^{{\rm{P}}{\rm{y}}{\rm{l}}}$ from cells expressing PylRS and provided with BocK (**1**) (Fig. 1b). The Cy3 signal resulting from the tRNA–DNA probe hybrid provided a measure of tRNA abundance (Supplementary Fig. 2). These experiments demonstrated that our approach, using fluoro-tREX, enables the acylation of a tRNA to be followed via the generation of a fluorescent signal. We confirmed that fluoro-tREX has a wide dynamic range and can detect the activity of synthetase variants with low activities (Supplementary Fig. 3).

As we sought to use tRNA extension-based methods to detect tRNAs that were acylated with monomers beyond α-l-amino acids, we wanted to ensure that following periodate oxidation, a range of monomers could be hydrolysed from the tRNA—this hydrolysis is required to generate the acylation-dependent signal in tREX-based approaches. The rate of ester hydrolysis for deacylating an acylated tRNA is expected to be inversely related to the p*K*_a_ (the negative logarithm of the acid dissociation constant, *K*_a_) of the carboxylic acid in the monomer that acylated the tRNA^23^. We acylated ${{\rm{tRNA}}}_{{\rm{C}}{\rm{U}}{\rm{A}}}^{{\rm{P}}{\rm{y}}{\rm{l}}}$ with the α-l-amino acid BocK (**1**), its hydroxy acid analogue, and its desamino carboxylic acid analogue^24^ (Supplementary Fig. 4); the calculated p*K*_a_ of these monomers is about 2.3, 3.7, and 4.6 respectively^25^. Although we detected acylation by the α-l-amino acid with our initial fluoro-tREX protocol, we did not detect acylation of tRNAs by the hydroxy acid or simple carboxylic acid; we hypothesized that this difference in detection was due to incomplete deacylation of the tRNAs loaded with the hydroxy acid or simple carboxylic acid following oxidation, in our fluoro-tREX protocol. By treating tRNAs with base, following oxidation, we improved the detection of acylation for all monomers tested. These results suggested that our revised protocol would enable detection of tRNA acylation for an extended set of monomers.

Next, we replaced Cy5–dCTP with biotinylated dCTP (bio–dCTP) in the extension step of fluoro-tREX, thereby creating biotin-tREX (bio–tREX). We selectively captured the biotinylated extension product (resulting from tRNA molecules that were protected from periodate oxidation by their aminoacylation) on streptavidin beads, washed the beads, and eluted bound tRNA extension products. The presence of the ${{\rm{tRNA}}}_{{\rm{C}}{\rm{U}}{\rm{A}}}^{{\rm{P}}{\rm{y}}{\rm{l}}}$ extension product in the eluate was dependent on the presence of PylRS and BocK (**1**) in cells, and the addition of bio–dCTP to the extension reaction (Fig. 1c). We conclude that bio–tREX enabled the selective capture of tRNA extension products from tRNAs that were acylated.

## Production and acylation of split tRNAs

**Fig. 2:**
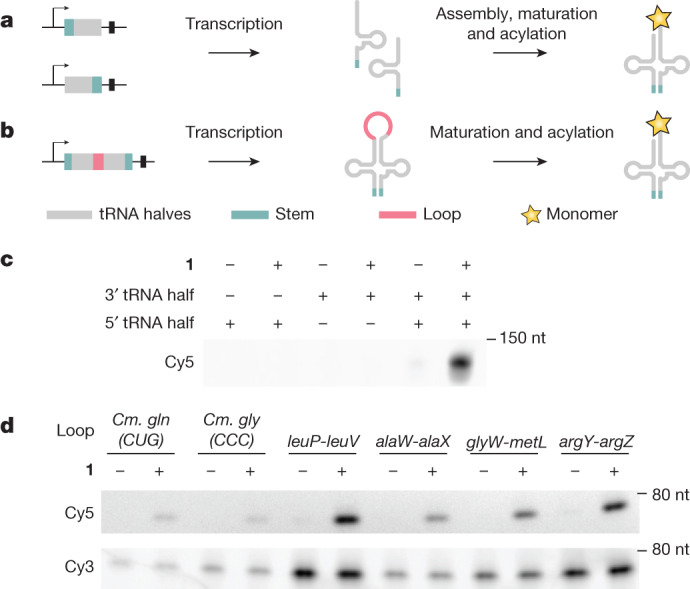
Production and acylation of split tRNAs expressed from split and circularly permuted genes. **a**, Schematic for producing split tRNAs in *trans*. The tRNA gene is split at the anticodon loop and the anticodon stem sequence is extended for optimal assembly of the transcribed RNA in vivo; this creates two genes: one for the 5′ half and one for the 3′ half of the split tRNA. These genes are transcribed and the split tRNA is assembled, matured and acylated in cells. **b**, Schematic for producing split tRNAs in *cis* from a single gene. The tRNA sequence is circularly permutated by connecting the 3′ half, via an intervening loop sequence, to the 5′ half, splitting the sequence at the anticodon and extending the anticodon stem. Transcription, assembly in *cis* and maturation leads to a functional split tRNA. **c**, in vivo transcription, assembly, maturation and acylation of split tRNA^Pyl^ produced from genes for the 5′ half and 3′ half. Cells were grown in the presence of PylRS. Only the expression of both tRNA^Pyl^ halves led to a BocK (**1**)-dependent acylation signal, as judged by fluoro-tREX. Note that under the purification conditions used to isolate these stRNAs, we do not observe the Cy3 probe. **d**, Circularly permutated split tRNA^Pyl^ with different loop sequences were assayed by fluoro-tREX. For the *argY*–*argZ* and *leuP*–*leuV* loops derived from the intergenic regions of pairs of tRNA genes in *E. coli*, the fluoro-tREX signal for split tRNA production (Cy3) and acylation (Cy5) was comparable to the corresponding signal for intact tRNA^Pyl^ (Supplementary Fig. 6). Experiments in **c**,**d** were repeated three times with similar results.

Next, we tested whether we could split the ${{\rm{tRNA}}}_{{\rm{C}}{\rm{U}}{\rm{A}}}^{{\rm{P}}{\rm{y}}{\rm{l}}}$ gene at the anticodon to create a split tRNA^Pyl^ (stRNA^Pyl^). We envisioned a system in which the 5′ and 3′ halves of a split tRNA gene were transcribed, assembled in vivo via non-covalent interactions (including base-pairing), matured by the cellular tRNA processing machinery, and recognized and efficiently acylated by PylRS, which does not recognize the anticodon^26,27^ (Fig. 2a,b).

We first designed a series of constructs in which we split the tRNA gene at the anticodon. We replaced the sequence of the anticodon stem loop in each half of the ${{\rm{tRNA}}}_{{\rm{C}}{\rm{U}}{\rm{A}}}^{{\rm{P}}{\rm{y}}{\rm{l}}}$ gene with an extension of 0 to 14 nucleotides in length; the extensions within each pair of tRNA halves were designed to base pair with each other and form a stem to stabilize the stRNA^Pyl^.

We expressed each pair of tRNA halves in *trans* (from two different plasmids) in the presence or absence of PylRS and BocK (**1**). We analysed the acylation of stRNA^Pyl^ using fluoro-tREX, as a measure of stRNA^Pyl^ assembly and function. For the stRNA^Pyl^ molecules with a stem length of 0 and 8 base pairs (bp), we observed attenuated acylation, and these constructs may not stably assemble in cells (Supplementary Fig. 5). For stRNA^Pyl^ molecules with stems comprising more than 12 bp, we observed gel bands consistent with degradation products (Supplementary Fig. 5). Stems of 10 or 12 bp resulted in robust aminoacylation, which was dependent on the presence of both tRNA halves, PylRS and BocK (**1**) (Fig. 2c). We concluded that a 10-bp stem is sufficient to facilitate the association of the 2 tRNA halves, minimize degradation and enable robust aminoacylation of the assembled tRNA. We therefore performed all subsequent experiments with stRNA^Pyl^ molecules with a 10-bp stem. To our knowledge, this is the first demonstration of a split tRNA being assembled, matured and acylated in cells.

Next, we focused on designing an expression system for stRNA^Pyl^ that would both ensure equimolar stoichiometry of both tRNA halves and facilitate spatial proximity, and thereby the assembly, of the two halves. We hypothesized that we could produce the two tRNA halves in *cis* from one transcript by inserting an intervening sequence between them, thereby creating a circular permutation of the parent tRNA. Processing to remove the intervening sequence would yield the stRNA (Fig. 2b).

We noted that the intergenic regions of polycistronic tRNA operons in *E. coli* connect the 3′ end of a tRNA gene to the 5′ end of the following tRNA gene and are efficiently removed from the resulting transcripts^28^. We used the tRNA operon generator^29^ to select four *E. coli* intergenic regions (and also selected two intervening sequences from circularly permuted *Cyanidioschyzon merolae* tRNA genes^30^) as the intervening sequences for our circular permutation strategy.

We created *cis* stRNA^Pyl^ genes in which the 3′ half of the stRNA^Pyl^ gene was connected through each selected intervening ‘loop’ sequence to the 5′ half of the split tRNA gene. Fluoro-tREX revealed efficient expression of stRNAs from *cis* stRNA^Pyl^ genes (Cy3 signal), and substantial, BocK (**1**)-dependent acylation of the stRNA^Pyl^ molecules produced from the *cis* stRNA^Pyl^ genes (Cy5 signal) in the presence of PylRS (Fig. 2d). *cis* stRNA^Pyl^ genes with the intergenic regions from *E. coli* produced stRNA^Pyl^ at levels comparable to ${{\rm{tRNA}}}_{{\rm{C}}{\rm{U}}{\rm{A}}}^{{\rm{P}}{\rm{y}}{\rm{l}}}$, and stRNA^Pyl^ was acylated at comparable levels to ${{\rm{tRNA}}}_{{\rm{C}}{\rm{U}}{\rm{A}}}^{{\rm{P}}{\rm{y}}{\rm{l}}}$ (Supplementary Fig. 6). We used the *E. coli* intergenic region *leuP–leuV* for all further experiments. We conclude that ${{\rm{tRNA}}}_{{\rm{C}}{\rm{U}}{\rm{A}}}^{{\rm{P}}{\rm{y}}{\rm{l}}}$ can be split and expressed from a single transcript in *cis*, and that the split tRNA functions as an efficient substrate for acylation by PylRS.

## Linking acylation phenotype and genotype

**Fig. 3:**
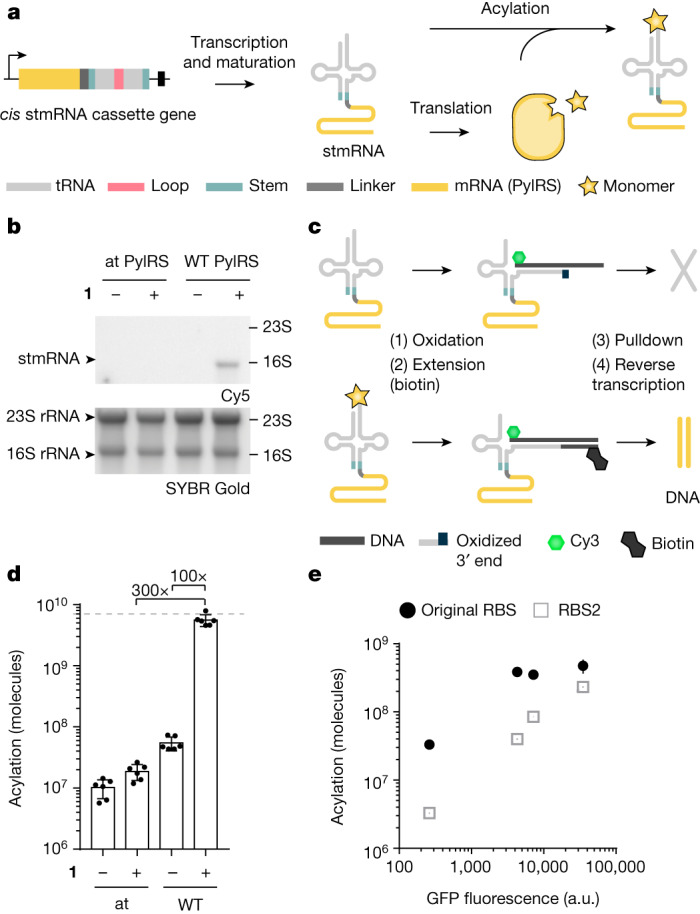
stmRNAs enable selective isolation of active PylRS variants. **a**, Schematic of the *cis* split tRNA–mRNA fusion (stmRNA) gene, and the production and acylation of stmRNA. **b**, stmRNA acylation visualized by fluorescent mRNA extension (fluoro-mREX). Cells harboured an stmRNA gene, wild-type (WT) or attenuated (at) PylRS. Positions of 16S (1.5 kb) and 23S rRNA (2.9 kb) are indicated. The fusion between the 3′ half of the tRNA and the mRNA is 1.5 kb. The fluoro-mREX signal was visualized on denaturing gels; representative of three independent replicates. **c**, Schematic of biotin mRNA extension (bio–mREX). Biotinylated stmRNAs are enriched on streptavidin beads. The mRNA of PylRS within is reverse transcribed on the beads and quantified by qPCR. **d**, Efficient and selective isolation of active PylRS variant cDNA via bio–mREX. Cells harbouring the indicated stmRNA were grown in the presence or absence of BocK (**1**) and bio–mREX was performed. Following pulldown and reverse transcription, we determined the number of cDNA molecules. Dashed line indicates 2.5% of input (Supplementary Fig. 8). The bars represent the mean of five biological replicates, dots represent individual data points, and error bars show the s.d. **e**, Tailoring the RBS of PylRS mRNAs within stmRNAs leads to a stronger correlation between the acylation of stmRNAs and readthrough of an amber stop codon (original RBS: *R*^2^ = 0.4694, *P* = 0.3148; RBS2: *R*^2^ = 0.9742, *P* = 0.013). Bio–mREX was performed from cells harbouring stmRNA genes encoding PylRS(CbzK1–4) with either the original RBS or RBS2 grown in the presence of CbzK (**2**). The measured cDNA molecules were plotted against the fluorescence intensity of GFP(150CbzK)–His_6_, resulting from readthrough of the amber codon in GFP(150TAG)His_6_ by each PylRS variant paired with ${{\rm{tRNA}}}_{{\rm{C}}{\rm{U}}{\rm{A}}}^{{\rm{P}}{\rm{y}}{\rm{l}}}$ in cells provided with CbzK (**2**). Bio–mREX was performed in duplicates and GFP fluorescence was measured in triplicates. Error bars show the s.d. a.u., arbitrary units.

Next, we fused the PylRS coding sequence and a linker sequence to the 5′ end of the 3′ half of the *cis* stRNA^Pyl^. We put the resulting stmRNA^Pyl^ cassette (Fig. 3a) under the control of an inducible *T7* promoter to maximize its transcription. We hypothesized that transcription, processing and maturation of this construct would lead to an stmRNA in which the mRNA encoding the synthetase was covalently linked to the 5′ end of the 3′ half of the stRNA, and associated with the 5′ half of the stRNA. Translation of the synthetase mRNA within the stmRNA^Pyl^ would generate the synthetase protein, which—in the presence of its cognate ncM—would acylate the stmRNA^Pyl^ in the same cell (Fig. 3a). This would generate a covalent link between the monomer attached to the tRNA and the mRNA of the synthetase gene that catalysed the attachment.

We grew cells expressing stmRNAs in the presence and absence of BocK (**1**), isolated total RNA and performed fluoro-mREX, a modification of fluoro-tREX for larger RNAs. For the wild-type stmRNA we observed a BocK (**1**)-dependent fluorescent (Cy5) band in fluoro-mREX (Fig. 3b). We did not observe a fluorescent signal for stmRNA^at^ (an stmRNA containing a PylRS gene encoding a protein with attenuated activity; Supplementary Fig. 7) in fluoro-mREX (Fig. 3b).

We conclude that our stmRNA construct is functional; it is transcribed and processed to generate a split tRNA in which the 3′ half is fused to the synthetase mRNA. The synthetase mRNA is translated, and the resulting protein catalyses the acylation of the 3′ end of the 3′ half of the stmRNA. In fluoro-mREX the acylation is converted into a fluorescent ‘phenotype’, thereby creating a physical linkage between the mRNA sequence of the synthetase, its genotype, and the fluorescent phenotype, generated as a result of the activity of the synthetase.

## Acylation-specific enrichment of PylRS

Next, we aimed to selectively isolate acylated stmRNA with respect to non-acylated stmRNA and reverse transcribe the isolated PylRS mRNA within stmRNA to directly yield the cDNA of the PylRS gene responsible for cellular acylation (Fig. 3c).

To selectively isolate acylated stmRNAs with respect to non-acylated stmRNAs we created bio–mREX (Fig. 3c), an adaptation of bio–tREX, in which we used the same methods to isolate RNA that we used for fluoro-mREX. In this approach, the biotinylated stmRNAs (which result from aminoacylated stmRNAs) are selectively captured on streptavidin beads. The PylRS mRNAs are then directly reverse transcribed from the stmRNA captured on the beads, to create PylRS cDNA. The PylRS cDNA is then released by RNase H treatment and heating, and quantified by quantitative PCR (qPCR).

To test our approach, we grew *E. coli* cells harbouring the stmRNAs in the presence and absence of BocK (**1**) and performed bio–mREX. We recovered 100-fold more cDNA molecules for wild-type stmRNA in the presence of BocK (**1**) than in the absence of BocK (**1**) (Fig. 3d). Moreover, we recovered 300-fold more DNA molecules from wild-type stmRNA with BocK (**1**) than from stmRNA^at^ (Fig. 3d, Supplementary Fig. 8 and Supplementary Data 1). We concluded that bio–mREX enables the selective recovery of stmRNAs and the genes for synthetases that acylate the split tRNAs within them.

Additional experiments suggested that above a threshold activity, the first generation of bio–mREX could not effectively differentiate between PylRS variants with different acylation activities (Fig. 3e, Extended Data Fig. 3, Supplementary Note 1, Supplementary Fig. 9 and Supplementary Data 1). To increase the dynamic range of the bio–mREX system, we attenuated the level of PylRS protein by tuning the ribosome binding site (RBS) from which it is expressed within the stmRNA. We designed 5′ untranslated region sequences with predicted attenuated translation rates^31^ and introduced them into the mRNAs for PylRS variants of varying activity within stmRNA. For all new RBS sequences, the correlation between GFP expression resulting from amber suppression and the number of molecules recovered by bio–mREX increased with respect to the original construct (Fig. 3e, Supplementary Fig. 9 and Supplementary Data 1). RBS2 displayed a strong correlation and we proceeded to use the stmRNA utilizing this RBS, termed stmRNA^vol2^, for all subsequent experiments.

In conclusion, by combining the stmRNA^vol2^ construct with bio–mREX, we developed a pulldown to selectively isolate the cDNA of active PylRS variants and to effectively differentiate between variants with a range of activities.

## tRNA display

**Fig. 4:**
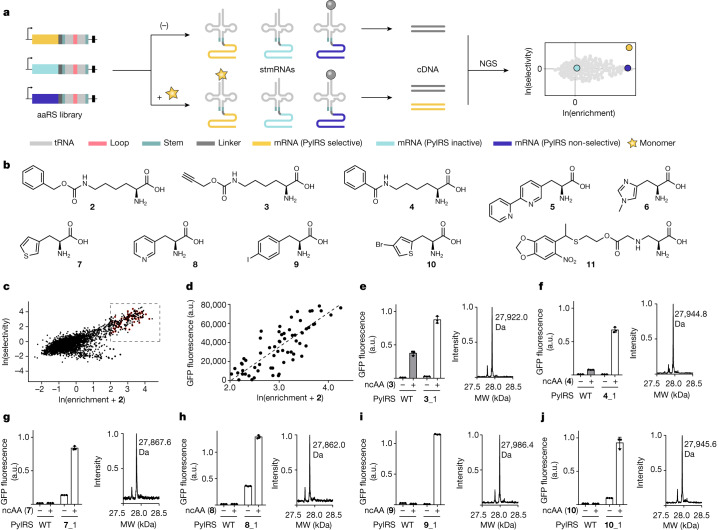
tRNA display enables the direct selection of orthogonal aminoacyl-tRNA synthetases that aminoacylate their cognate orthogonal tRNAs with ncAAs. **a**, Schematic of tRNA display. A library of aaRSs encoded within stmRNA genes is grown in the presence and absence of non-canonical monomers of interest (yellow star). Bio–mREX is performed and the cDNA is sequenced by NGS. The data are used to generate spindle plots. **b**, The numbered structures of non-canonical α-amino acids used in this study. **c**, tRNA display with stmRNA^vol2^-^lib1^. Spindle plot from one step tRNA display selection with stmRNA^vol2^-^lib1^ and CbzK (**2**). Samples were run in triplicate and data were processed as described in Methods. Red dots indicate 65 clones that were further characterized. **d**, Plot of ln(enrichment + **2**) of PylRS mutants derived from tRNA display (red dots in **c**) against GFP fluorescence from cells containing the corresponding PylRS mutant–${{\rm{tRNA}}}_{{\rm{C}}{\rm{U}}{\rm{A}}}^{{\rm{P}}{\rm{y}}{\rm{l}}}$ pair, GFP(150TAG)His_6_ and CbzK (**2**). The dotted line represents a linear regression for the data points. *R*^2^ = 0.6611, *P* < 0.0001. **e**–**j**, Left, GFP fluorescence from cells containing GFP(150TAG)His_6_, the indicated PylRS variant–${{\rm{tRNA}}}_{{\rm{C}}{\rm{U}}{\rm{A}}}^{{\rm{P}}{\rm{y}}{\rm{l}}}$ pair, and ncAA (white bar), or the wild-type PylRS–${{\rm{tRNA}}}_{{\rm{C}}{\rm{U}}{\rm{A}}}^{{\rm{P}}{\rm{y}}{\rm{l}}}$ pair with the same ncAA (grey bar). Fluorescence is plotted as a fraction of the signal generated by the wild-type PylRS– ${{\rm{tRNA}}}_{{\rm{C}}{\rm{U}}{\rm{A}}}^{{\rm{P}}{\rm{y}}{\rm{l}}}$ pair with 2 mM BocK (**1**) and GFP(150TAG)His_6_. Right, ESI–MS analysis of GFP(150X)–His_6_, where X is the indicated ncAA. **e**, Found mass: 27,922.0 Da, expected mass 27,923.3 Da. **f**, Found mass: 27,944.8 Da, expected mass 27,945.5 Da. **g**, Found mass: 27,867.6 Da, expected mass 27,866.4 Da. **h**, Found mass: 27,862.0 Da, expected mass 27,861.4 Da. **i**, Found mass: 27,986.4 Da, expected mass 27,986.2 Da. **j**, Found mass: 27,945.6 Da, expected mass 27,944.3 Da. Bars represent the mean of three biological replicates, data points are shown as dots, and error bars represent s.d. Mass spectrometry data are from single replicates.

We envisaged efficiently identifying PylRS variants from large libraries of mutants (in stmRNA^vol2^) that are active and selective for ncMs by running parallel bio–mREX-based selections, in the presence (positive sample) and absence (negative sample) of ncMs. The experiments would be performed in multiple replicates, and cDNA of the positive and negative samples, as well as the cDNA reverse transcribed from the input library, would subsequently be barcoded and sequenced by next-generation sequencing (NGS) (Fig. 4a). NGS data from the selections of the samples would enable the calculation of two key parameters: (1) the enrichment, defined as the average abundance of a particular sequence in the positive samples over the abundance of the same sequence in the input RNA; and (2) the selectivity, defined as the ratio of the abundance of a sequence in the positive sample over the abundance of the same sequence in the negative sample. Desired PylRS variants would have sequences that are highly enriched and selective (Fig. 4a). We termed this selection approach tRNA display.

To test tRNA display we generated a small PylRS library, in which we expected many sequences to be active, in stmRNA^vol2^. The library targeted positions Y306, L309 and N346 in PylRS, where mutations had previously been identified^32,33^ that enable the efficient incorporation of CbzK (**2**) (Fig. 4b and Extended Data Fig. 4). Following a single round of selection, we observed a large population of highly enriched and selective variant PylRS sequences in the spindle plot derived from the sequencing of this tRNA display experiment (Fig. 4c).

To assess the predictive power of tRNA display, we measured the in vivo production of GFP(150CbzK)–His_6_ (His_6_-tagged GFP protein with a CbzK substitution at position 150) from GFP(150TAG)His_6_ in the presence of ${{\rm{tRNA}}}_{{\rm{C}}{\rm{U}}{\rm{A}}}^{{\rm{P}}{\rm{y}}{\rm{l}}}$ and 65 PylRS variants, which were hits on the basis of their position on the spindle plot (Supplementary Fig. 10). We observed a positive correlation between the enrichment derived from NGS data and the translation activity derived from GFP production for these hits (Fig. 4d), and found the vast majority of these hits to be selective (Supplementary Fig. 10). We concluded that tRNA display enables the direct, translation independent, identification of active and selective PylRS enzymes from a library of PylRS sequences.

## Scalable tRNA display-based discovery

**Extended Data Fig. 2:**
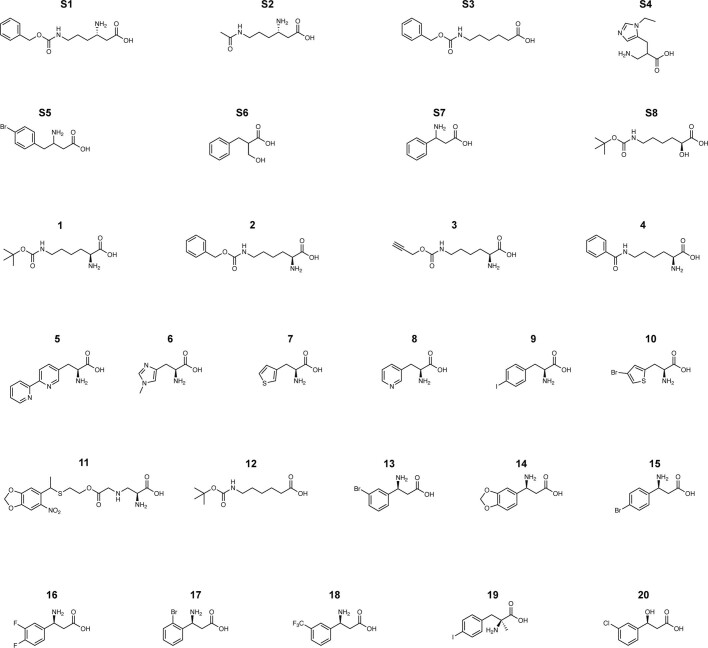
Chemical structures of all compounds used in this study. (*S*)-3-amino-6-(((benzyloxy)carbonyl)amino)hexanoic acid ((*S*)-β^3^CbzK) (**S1**), (*S*)-6-acetamido-3-aminohexanoic acid ((*S*)-β^3^AcK) (**S2**), 6-(((benzyloxy)carbonyl)amino)hexanoic acid (CbzAhx) (**S3**), 3-amino-2-((1-ethyl-1*H*-imidazol-5-yl)methyl)propanoic acid (β^2^NeH (**S4**)), 3-amino-4-(4-bromophenyl)butanoic acid (β^3^pBrhF) (**S5**), 2-benzyl-3-hydroxypropanoic acid (β^2^OH-F) (**S6**), 3-amino-3-phenylpropanoic acid (β^3^F) (**S7**), (*S*)-*6*-((tert-butoxycarbonyl)amino)-2-hydroxyhexanoic acid (OH-BocK) (**S8**). *N*^*6*^-(*tert*-butoxycarbonyl)-*L*-lysine (BocK) (**1**), *N*^6^-((benzyloxy)carbonyl)-*L*-lysine (CbzK) (**2**), *N*^6^-((prop-2-yn-1-yloxy)carbonyl)-*L*-lysine (AlkyneK) (**3**), *N*^6^-benzoyl-*L*-lysine (BenzK) (**4**), 3-([2,2’-bipyridin]-5-yl)-2-aminopropanoic acid (BiPyA) (**5**), *N*^τ^-methyl-*L*-histidine (N^τ^mH) (**6**), (*S*)-2-amino-3-(thiophen-3-yl)propanoic acid (3-ThiA) (**7**), (*S*)-2-amino-3-(pyridin-3-yl)propanoic acid (PyA) (**8**), (*S*)-2-amino-3-(4-iodophenyl)propanoic acid (pIF) (**9**), (*S*)-2-amino-3-(4-bromothiophen-2-yl)propanoic acid (BrThiA) (**10**), (2 *S*)-2-amino-3-(((2-((1-(6-nitrobenzo[*d*][1,3]dioxol-5yl)ethyl)thio)ethoxy)carbonyl)amino)propanoic acid (pcDAP) (**11**), *N*^*6*^-(*tert*-butoxycarbonyl)-aminohexanoic acid (BocAhx) (**12**), (*S*)-3-amino-3-(3-bromophenyl)propanoic acid ((*S*)β^3^mBrF) (**13**), (*S*)-3-amino-3-(benzo[*d*][1,3]dioxol-5-yl)propanoic acid ((*S*)β^3^MDF) (**14**), (*S*)-3-amino-3-(4-bromophenyl)propanoic acid ((*S*)β^3^pBrF) (**15**), (*S*)-3-amino-3-(3,4-difluorophenyl)propanoic acid ((*S*)β^3^pmFF) (**16**), (*S*)-3-amino-3-(2-bromophenyl)propanoic acid ((*S*)β^3^oBrF) (**17**), (*S*)-3-amino-3-(3-(trifluoromethyl)phenyl)propanoic acid (**18**) ((*S*)β^3^mCF_3_F), (*S*)-2-amino-3-(4-iodophenyl)-2-methylpropanoic acid (**19**) ((*S*)α-Me-pIF), (*S*)-3-(3-chlorophenyl)-3-hydroxypropanoic acid (**20**) (OH-(*S*)β^3^mClF).

**Extended Data Fig. 3:**
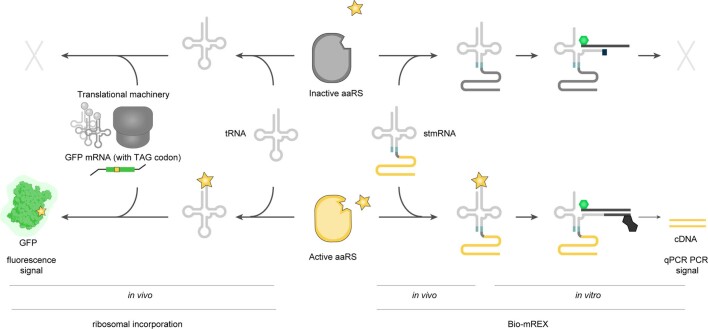
Relationship between cDNA retrieved from bio-mREX and fluorescence obtained from in vivo genetic code expansion. Relationship between the acylation signal measured, by bio-mREX, for stmRNAs and the GFP fluorescence signal measured for intact, translation-competent tRNAs. For stmRNAs, active aminoacyl-tRNA synthetases (aaRS) lead to the acylation of their encoding stmRNAs, which by bio-mREX get extended, separated and ultimately reverse transcribed. This results in the cDNA of the active synthetase, which can be quantified by qPCR. In the case of an inactive aaRS the stmRNAs is not acylated and no cDNA is produced in bio-mREX experiments. Therefore, the activity of a synthetase in bio-mREX correlates with the number of cDNA molecules measured by qPCR. In canonical translation an active aaRS enzyme leads to an acylated, intact, cognate tRNA_CUA_ which is used in protein translation. Inactive aaRS enzymes lead to non-acylated tRNAs, which are not used in protein translation. The production of GFP protein from *GFP(150TAG)His*_6_, as measured by GFP fluorescence, reports on the acylation of tRNA_CUA_, as well as the other steps in the production of protein.

**Extended Data Fig. 4:**
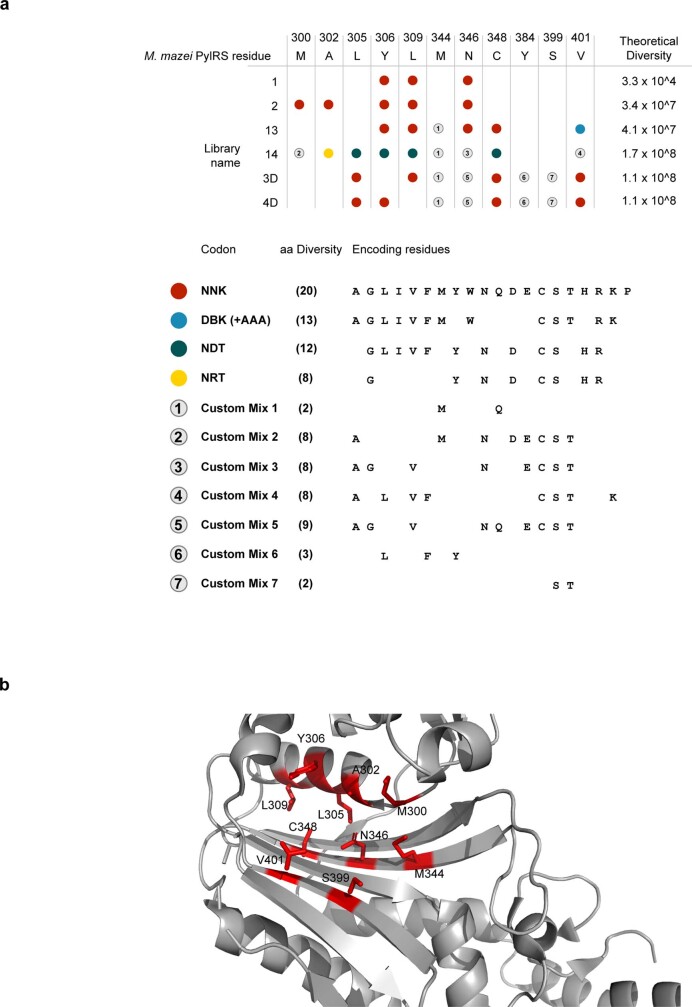
Library design for this study. PylRS libraries used in this work. **a**, Overview of the seven libraries designed and created. These libraries target a total of 11 amino acid residues in the PylRS active site and employed several types of degenerate codons. NNK codons are depcited as dark red, DBK codons (+lysine codon) as blue, NDT codons as dark green, NRT codons as yellow. For certain sites, custom residue mixes encompassing the most commonly observed mutations were used (1-7 mixes, depcited as grey spheres). All libraries were created with at least 10^9^ independent transformants. N = A, T, G, C; K = G, T; D = G, A, T; B = G, T, C; R = G, A. The custom mixes are described in the methods. **b**, The eleven amino acid residues targeted for mutagenesis in the PylRS active site are shown in red. Image was rendered using Pymol, based on the PDB structure 2ZIN.

To further validate the utility of tRNA display, we ran parallel selections (Supplementary Fig. 11) using six, highly diverse PylRS active site libraries (Extended Data Fig. 4) and ten ncAAs (**2**–**11**) (Fig. 4b and Extended Data Fig. 2). After two rounds of selection, we analysed the spindle plots derived from the NGS data and identified individual PylRS mutants for ncAAs **2**, **3**, **4**, **7**, **8**, **9**, **10** and **11** that were enriched and selective; the enriched and selective mutants for each of these ncAAs showed convergent sequence motifs (Supplementary Figs. 12–21).

We demonstrated the incorporation of ncAAs **3**, **4**, **7**, **8**, **9**, **10** and **11** in response to the amber codon in GFP(150TAG)His_6_ in cells containing ${{\rm{tRNA}}}_{{\rm{C}}{\rm{U}}{\rm{A}}}^{{\rm{P}}{\rm{y}}{\rm{l}}}$ and the corresponding PylRS mutants identified by tRNA display. GFP production was dependent on the presence of ncAAs, and electrospray ionization mass spectrometry (ESI–MS) confirmed the incorporation of each ncAA in GFP (Fig. 4e–j, Supplementary Figs. 12–21 and Supplementary Data 1). We note that our results include an aminoacyl-tRNA synthetase for **7**, which, to our knowledge, enables the first incorporation of this thiophene containing ncAA into a protein. Out of the 30 characterized variants with a selectivity score greater than or equal to 10 and an enrichment score greater than or equal to 5, 27 were active with their cognate ncAA in protein expression (20 variants were active at levels corresponding to at least 50% of the activity of PylRS with BocK (**1**)). All 27 variants selectively incorporated their ncAA substrate, as judged by mass spectrometry (Fig. 4e–j and Supplementary Figs. 12–21).

In summary, we demonstrated the parallel, scalable and rapid selection of PylRS variants using diverse libraries and ncAAs through tRNA display. We note that the amount of ncAA used in each tRNA display selection was 50–100 times lower than that used in current methods for synthetase selection.

## Selective orthogonal pairs for ncMs

**Fig. 5:**
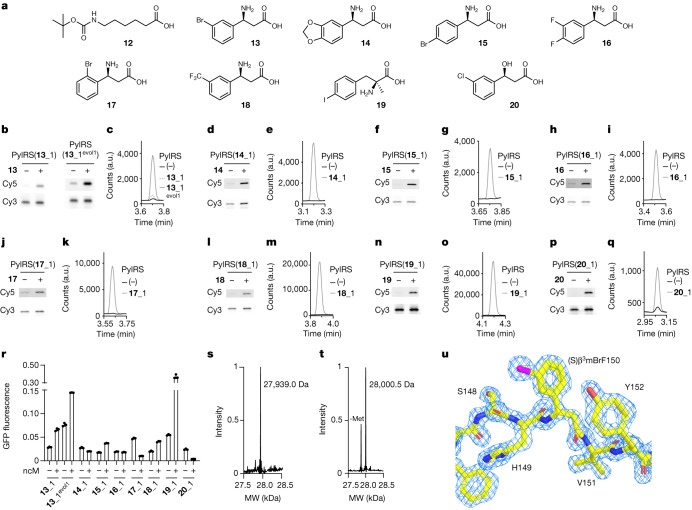
tRNA display selection of orthogonal synthetases that charge ncMs. **a**, ncMs for which selective PylRS mutants were discovered. **b**, Fluoro-tREX. A representative gel is shown for each PylRS variant. Experiments performed in independent triplicates with similar results. **c**, Selected PylRS variants acylate ${{\rm{tRNA}}}_{{\rm{C}}{\rm{U}}{\rm{A}}}^{{\rm{P}}{\rm{y}}{\rm{l}}}$ with **13**. LC–MS traces (scanning ion mode on 6-aminoquinolyl-*N*-hydroxysuccinimidyl carbamate (AQC) adduct of **13**) of AQC-derivative eluted from tRNA pulldowns. Cells harbouring the corresponding PylRS variant and ${{\rm{tRNA}}}_{{\rm{C}}{\rm{U}}{\rm{A}}}^{{\rm{P}}{\rm{y}}{\rm{l}}}$ (or only ${{\rm{tRNA}}}_{{\rm{C}}{\rm{U}}{\rm{A}}}^{{\rm{P}}{\rm{y}}{\rm{l}}}$ (−)) were grown with **13**. Pulldowns used a biotinylated probe against ${{\rm{tRNA}}}_{{\rm{C}}{\rm{U}}{\rm{A}}}^{{\rm{P}}{\rm{y}}{\rm{l}}}$. Representative traces are shown. **d**,**f**,**h**,**j**,**l**,**n**,**p**, As in **b**, but with indicated PylRS variants and **14** (**d**), **15** (**f**), **16** (**h**), **17** (**j**), **18** (**l**), **19** (**n**) or **20** (**p**). **e**,**g**,**i**,**k**,**m**,**o**,**q**, As in **c**, but with indicated PylRS variants and **14** (**e**), **15** (**g**), **16** (**i**), **17** (**k**), **18** (**m**), **19** (**o**) or **20** (**q**). **e**,**g**,**i**, Performed in duplicate, all replicates yielded similar results. **c**,**k**,**m**,**o**,**q**, Performed in triplicate, all replicates yielded similar results. **r**, Fluorescence from cells containing GFP(150TAG)His_6_, indicated PylRS variant and ${{\rm{tRNA}}}_{{\rm{C}}{\rm{U}}{\rm{A}}}^{{\rm{P}}{\rm{y}}{\rm{l}}}$, and grown in the presence or absence of ncM (**13**–**20**, 4 mM). Fluorescence is shown as a fraction of the fluorescence generated by the wild-type PylRS–${{\rm{tRNA}}}_{{\rm{C}}{\rm{U}}{\rm{A}}}^{{\rm{P}}{\rm{y}}{\rm{l}}}$ pair with 4 mM BocK (**1**) and GFP(150TAG)His_6_. Bar graphs represent mean of three independent measurements, individual data points are shown as dots and error bars indicate s.d. **s**, ESI–MS of GFP(150(*S*)β^3^mBrF)–His_6_ purified from cells harbouring PylRS(**13**_1^evol1^), ${{\rm{tRNA}}}_{{\rm{C}}{\rm{U}}{\rm{A}}}^{{\rm{P}}{\rm{y}}{\rm{l}}}$ and GFP(150TAG)_His6_ grown with **13**. Found mass: 27,939.0 Da, predicted mass: 27,938.2 Da. Spectra acquired once. **t**, ESI–MS of GFP(150(*S*)α-Me-pIF)–His_6_ purified from cells harbouring PylRS(**19**_1), ${{\rm{tRNA}}}_{{\rm{C}}{\rm{U}}{\rm{A}}}^{{\rm{P}}{\rm{y}}{\rm{l}}}$ and GFP(150TAG)_His6_ grown with **19**. Found mass: 28,000.5 Da, predicted mass: 28,000.2 Da. Spectra were acquired once. **u**, Close up on residue 150 of GFP(150(*S*)β^3^mBrF)–His_6_, from a crystal structure determined at 1.5 Å. The 2*F*_o_ − *F*_c_ map is shown at contour level of *σ* = 2 (Protein Data Bank (PDB) ID 8OVY), electron density (blue).

Next, we challenged tRNA display to discover synthetases that are selective for classes of ncMs that either cannot be translated or are likely to be poor ribosomal substrates^13,15^ (Fig. 5, Extended Data Fig. 2 and Supplementary Fig. 22).

To select PylRS variants for selected monomers, we ran a single round of parallel tRNA display selections (Supplementary Fig. 22) using a highly diverse library (lib14), which mutated residues 300, 302, 305, 306, 309, 344, 346, 348 and 401 in the PylRS active site, and a collection of ncMs (Extended Data Fig. 4). We analysed the spindle plots derived from the NGS data for selections with each ncM to identify PylRS variants that were enriched and selective (Supplementary Figs. 23–31). For ncMs **12** (6-((*tert*-butoxycarbonyl)amino)hexanoic acid (BocAhx)) and **13** ((*S*)-3-amino-3-(3-bromophenyl)propanoic acid (β^3^-mBrF)), we identified enriched and selective sequences, and found that the most selective hits converged on a distinct sequence pattern for each of these ncM.

The four most selective and active hits from the selection with **12**, PylRS(**12**_1) to PylRS(**12**_4), differ from the wild-type sequence by point mutations of V401. We showed, by fluoro-tREX, that all of these selected sequences were active and selective for **12** (Supplementary Fig. 23).

**Extended Data Fig. 5:**
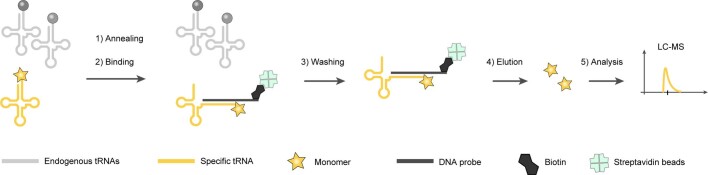
Assay for determination of acylating monomer identity. Schematic of tRNA pulldown followed by LC-MS analysis to determine the identity of the monomer on the target tRNA. tRNAs are extracted from cells expressing the tRNA of interest and the cognate orthogonal aaRS, grown in the presence of the ncM. A biotinylated probe is annealed, and the targeted tRNA is pulled down. After washing, the ncM is eluted by alkaline deacylation, derivatised with 6-aminoquinolyl-*N*-hydroxysuccinimidyl carbamate (AQC), and detected using LC-MS.

Notably, two PylRS variants, PylRS(**13**_1) and PylRS(**13**_2), identified by tRNA display selection, directed **13**-dependent acylation of ${{\rm{tRNA}}}_{{\rm{C}}{\rm{U}}{\rm{A}}}^{{\rm{P}}{\rm{y}}{\rm{l}}}$, as judged by fluoro-tREX (Fig. 5c and Supplementary Fig. 24). To verify the identity of the monomer attached to the tRNA by PylRS(**13**_1) and PylRS(**13**_2), we captured the acylated ${{\rm{tRNA}}}_{{\rm{C}}{\rm{U}}{\rm{A}}}^{{\rm{P}}{\rm{y}}{\rm{l}}}$ on streptavidin beads via a biotinylated probe for ${{\rm{tRNA}}}_{{\rm{C}}{\rm{U}}{\rm{A}}}^{{\rm{P}}{\rm{y}}{\rm{l}}}$, washed the beads and eluted the ncM by heating under alkaline conditions. We then derivatized the free ncM and analysed the sample by liquid chromatography–mass spectrometry (LC–MS) (Extended Data Fig. 5). Using this approach, we confirmed that both PylRS variants charged ${{\rm{tRNA}}}_{{\rm{C}}{\rm{U}}{\rm{A}}}^{{\rm{P}}{\rm{y}}{\rm{l}}}$ with the ncM **13** (Fig. 5b and Supplementary Fig. 32).

Next we increased the activity of PylRS(**13**_1) and PylRS(**13**_2) by random mutagenesis of the active site region of the PylRS gene within the stmRNA construct followed by tRNA display-based selection (Supplementary Figs. 33 and 34). Three of the most enriched and selective hits from the resulting spindle plot, PylRS(**13**_1^evol1–3^), were derivatives of PylRS(**13**_1). We confirmed the specificity of PylRS(**13**_1^evol1–3^) for acylating ${{\rm{tRNA}}}_{{\rm{C}}{\rm{U}}{\rm{A}}}^{{\rm{P}}{\rm{y}}{\rm{l}}}$ with **13**, by both fluoro-tREX and our LC–MS-based assay (Fig. 5b,c and Supplementary Figs. 32 and 34). PylRS(**13**_1^evol1–3^) were notably more active in acylating ${{\rm{tRNA}}}_{{\rm{C}}{\rm{U}}{\rm{A}}}^{{\rm{P}}{\rm{y}}{\rm{l}}}$ with **13** than PylRS(**13**_1**)** (Fig. 5b,c).

Next, we performed two rounds of selection with lib14 and ncMs **14**, **15**, **16**, **17** and **18** (β-amino acids with different side chains), **19** (an α,α-disubstituted amino acid) and **20** (a β-hydroxy acid) (Fig. 5a and Supplementary Fig. 35). From the resulting spindle plots (Supplementary Figs. 36–42), we identified enriched and selective sequences, and the most selective hits converged on a distinct sequence pattern for each ncM. We note that the synthetases selected for all six β-amino acids differ in sequence, but contain common mutations M300D and A302H (Supplementary Figs. 25 and 38–42). The sequence pattern observed for the β-hydroxy acid **20** is similar to the one observed for β-amino acids. However, the residue at position 300—which may be in direct proximity to the amine or hydroxy group—is changed from aspartic acid to asparagine (Supplementary Fig. 42). The PylRS variants identified by tRNA display selection directed the specific acylation of ${{\rm{tRNA}}}_{{\rm{C}}{\rm{U}}{\rm{A}}}^{{\rm{P}}{\rm{y}}{\rm{l}}}$ by their cognate monomer, as judged by fluoro-tREX and our LC–MS-based assay (Fig. 5d–q). We quantified the fraction of acylation as a function of ncM concentration (Supplementary Fig. 43 and Supplementary Data 1). To our knowledge, this is the first report of specific aminoacyl-tRNA synthetase–tRNA pairs for three distinct classes of ncM: β-amino acids, α,α-disubstituted amino acids and β-hydroxy acids.

## Encoding ncMs in proteins

To investigate the incorporation of the β-amino acids, α,α-disubstituted amino acids and β-hydroxy acids into proteins we added the orthogonal synthetase–orthogonal tRNA pairs for **13**–**20**, the cognate ncMs, and GFP(150TAG)His_6_ to cells. We observed ncM-dependent GFP production for **13**, **15**, **18** and **19**, with isolated yields ranging from 3 to 35 mg per litre of culture, and mass spectrometry confirmed the incorporation of these β-amino acids and α,α-disubstituted amino acids in GFP (Fig. 5r–t and Supplementary Figs. 44 and 45). In the absence of **13**, we observed some GFP production resulting from the incorporation of natural amino acids (Fig. 5r). However, in the presence of **13**, we produced more GFP, and we only detected incorporation of **13** by intact MS and MS/MS (Fig. 5s and Supplementary Fig. 44). We concluded that in the presence of **13**, the background incorporation observed in the absence of **13** was effectively outcompeted. We made similar observations for **15**, **18** and **19** (Fig. 5s,t and Supplementary Fig. 44). Similar observations have previously been made for efficient and selective ncAA incorporation systems^34^ and the fidelity of the natural code also relies on competition^35^. We concluded that **13**, **15**, **18** and **19** are site-specifically incorporated with high fidelity. We did not observe incorporation of ncMs **13**, **15**, **18** or **19** at position 3 of GFP (from GFP(3TAG)His_6_) (Supplementary Fig. 45), indicating that these ncMs are not tolerated at all positions in a protein. Similar site-dependent incorporation efficiency has previously been observed for ncAAs^36,37^.

We note that we did not observe an ncM-dependent increase in production of GFP from GFP(150TAG)His_6_ or GFP(3TAG)His_6_ with **14**, **16**, **17** or **20** when cells were provided with these ncMs and their cognate orthogonal synthetase–orthogonal tRNA pairs (Fig. 5r and Supplementary Fig. 45). These observations are consistent with these ncMs being poor substrates for ribosomal polymerization. For **17** and **20**, we observed a decrease in GFP production upon addition of ncM (Fig. 5r). This is consistent with these ncMs, once acylated onto the orthogonal tRNA, inhibiting readthrough of the amber codon. The discovery of orthogonal synthetases that are specific for these ncMs provides a starting point for selecting ribosomes that can efficiently polymerize them.

## Structure of β-amino acid-containing protein

**Extended Data Fig. 6:**
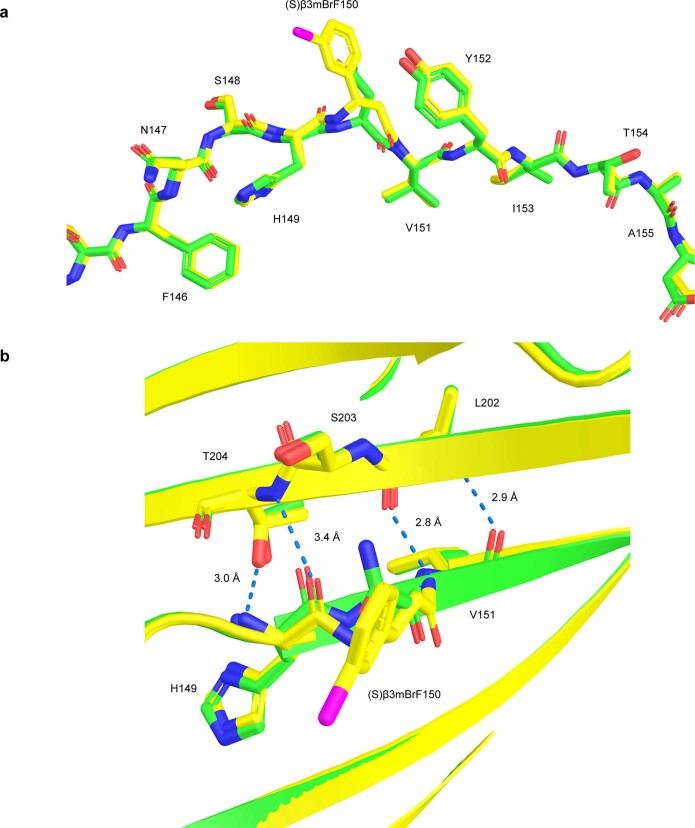
Detail of the structure of GFP containing a β-amino acid. Details of structure GFP(150(S)β^3^mBrF)–His_6_ (PDB code 8OVY). **a**, Detail of protein chain at position 150. The acquired structure for GFP(150(S)β^3^mBrF)–His_6_ (in yellow) is superimposed with the structure of wt GFP used for refinement (PDB: 2B3P), showing the kink in the backbone induced by the β^3^-amino acid. **b**, Detail of hydrogen bond network in the beta-barrel at position 150. The GFP(150(S)β^3^mBrF)–His_6_ structure (yellow) is superimposed to the structure of WT GFP (PDB: 2B3P). The kink induced by incorporation of (S)β^3^mBrF (**13**) affects the local hydrogen bond at that position, however the remaining contacts of the corresponding beta-strand remain intact.

To further characterize the incorporation of **13** at position 150 of GFP, we solved the structure of GFP(150(S)β^3^mBrF)–His_6_ at 1.5 Å resolution by X-ray crystallography (the protein was purified from cells harbouring PylRS(**13**_1) and ${{\rm{tRNA}}}_{{\rm{C}}{\rm{U}}{\rm{A}}}^{{\rm{P}}{\rm{y}}{\rm{l}}}$) (Fig. 5u and Extended Data Table 1). The electron density shows consecutive carbon atoms (C2 and C3) in the protein backbone at position 150; the meta-bromophenyl substituent is attached to C3 of the β-amino acid, and the stereochemistry at C3 corresponds to the expected (S) stereoisomer. Our structure confirms the site-specific incorporation of the expected (S)β^3^mBrF-monomer in the protein. The introduction of (S)β^3^mBrF leads to a notable kink in the beta strand of GFP, when compared to the wild-type protein (Extended Data Fig. 6). Notably, the hydrogen bonding networks of the residues immediately preceding and following the β-amino acid in the polypeptide chain remain essentially unperturbed; this indicates that this beta strand can accommodate the β^3^-amino acid at this position (Extended Data Fig. 6). To our knowledge, this represents the first solved structure of a protein produced in vivo that contains a genetically encoded β-amino acid.

## Discussion

The in vivo, site-specific incorporation of backbone-modified monomers is a longstanding challenge in expanding the scope of encoded cellular polymer synthesis beyond α-l-amino acids and their close analogues^9^.

tRNA display systematically breaks the deadlock (Extended Data Fig. 1) that has so far limited the range of monomers that can be used to specifically acylate orthogonal tRNAs in vivo. Using tRNA display, we have identified orthogonal synthetase–orthogonal tRNA pairs that are selective for eight new ncMs, including β-amino acids, α,α-disubstituted amino acids and β-hydroxy acids, and thereby directly facilitated the genetic encoding and site-specific incorporation of β-amino acids and α,α-disubstituted amino acids into proteins in a living organism.

tRNA display may be extended to other synthetase and tRNA systems to further expand the range of monomers that can be loaded onto tRNAs. The sequence, activity and selectivity data generated from tRNA display may facilitate the de novo design of active sites that are selective for new ncMs. Extensions of tRNA display may be used to select for genes that direct the biosynthesis of ncMs, or that bind to and protect tRNAs acylated with ncMs (for example, EF-Tu variants for ncMs^38–40^).

In future work we will leverage the cellular acylation of tRNAs with ncMs to enable translation-based selections for orthogonal ribosomes^34,41–43^ that can polymerize ncMs that are poor substrates for natural translation, and facilitate the encoded cellular synthesis of polymers composed of more diverse ncMs. The repertoire of non-canonical polymers may be further enhanced by leveraging post-translational modifications and ligations^44–49^.

The genetic encoding of β-amino acids and α,α-disubstituted amino acids may enable the creation of protease-resistant proteins and new drug-like molecules^50,51^ in living cells. Moreover, it may be possible to encode the biosynthesis of foldamers^52^ entirely composed of β-amino acids and other ncMs^53^ that complement and augment the canonical functions of living organisms.

## Methods

### Buffers

Resuspension buffer: 100 mM sodium acetate, 50 mM NaCl, 0.1 mM EDTA, pH 5.0; deacylation buffer: 50 mM bicine pH 9.6, 1 mM EDTA; Buffer D: 50 mM sodium acetate pH 5, 150 mM NaCl, 10 mM MgCl_2_, 0.1 mM EDTA; hybridization buffer: 10 mM Tris-HCl, 25 mM NaCl, pH 7.4; Klenow fragment exo− master mix with Cy5-11-dCTP (KMM–Cy5): 17 μl water, 5 μl 10x NEBuffer 2.0, 1 μl Klenow fragment exo− (NEB), 1 dNTPS-dCTP (10 mM), 1 μl Cy5-11-dCTP (20 μM); orange loading dye: 8 M urea, orange G; Klenow fragment exo− master mix mini (KMM -mini): 3.35 μl water, 1.2 μl 10x NEBuffer 2.0, 0.1 μl Klenow fragment exo−, 0.25 dNTPS (10 mM); Klenow fragment exo− master mix with Cy5-11-dCTP mini (KMM–Cy5-mini): 2.1 μl water, 1.2 μl 10x NEBuffer 2.0, 0.2 μl Klenow fragment exo−, 0.5 dNTPS (5 mM, without dCTP), 1 μl Cy5-11-dCTP (5 μM); Klenow fragment exo− master mix with Bio-11-dCTP (KMM-Bio): 17 μl water, 5 μl 10x NEBuffer 2.0, 1 μl Klenow fragment exo−, 1 dNTPS-dCTP (10 mM), 1 μl Bio-11-dCTP (20 μM); washing buffer: 10 mM Tris-HCl, 150 mM LiCl, 1 mM EDTA, 0.05% (v/v) Tween-20, pH 7.5; binding buffer: 20 mM Tris-HCl, 1 M LiCl, 2 mM EDTA, pH 7.5; formamide loading buffer (FLB): 90% formamide; SDS lysis buffer (SLB): 100 mM sodium acetate, 50 mM NaCl, 0.1 mM EDTA, pH 5.0, 1% (v/v) sodium dodecyl sulphate (SDS); alkaline washing buffer (AWB): 25 mM NaOH, 4 mM EDTA, 0.05% (v/v) Tween-20; reverse transcription hybridization mix (RHM): 1 μl DNA primer (2 μM), 1 μl 10 mM dNTPs, 1 μl 10x hybridization buffer, 10 μl water; reverse transcription master mix (RMM): 4 μl SSIV buffer, 1 μl RNAse Out, 1 μl SSIV reverse transcriptase, 1 μl 0.1 M dithiothreitol (DTT); acidic washing buffer 1(aWB1): 100 mM sodium acetate pH 5; acidic washing buffer 1 plus Tween-20 (aWB1-T): 100 mM sodium acetate pH 5, 0.01% (v/v) Tween-20; acidic washing buffer 2 (aWB2): 20 mM sodium acetate pH 5.

### Media

SOC: Super Optimal Broth plus 20 mM glucose; 2xYT-s: Yeast Extract Tryptone supplemented with 75 μg ml^−1^ spectinomycin; 2xYT-s-t: Yeast Extract Tryptone supplemented with 75 μg ml^−1^ spectinomycin and 10 μg ml^−1^ tetracycline; 2xYT-s-ap: Yeast Extract Tryptone supplemented with 75 μg ml^−1^ spectinomycin and 50 μg ml^−1^ apramycin; 2xYT-am: Yeast Extract Tryptone supplemented with 75 μg ml^−1^ ampicillin.

### Chemicals

NcAA **1** and **2** were purchased from Bachem. NcAA **4** was purchased from Fluorochem. NcAA **5** was purchased from Ambeed. NcAAs and ncMs **6**, **8**, **14**, **S1**, **S4** and **S5** were purchased from Enamine. NcAA **7** was purchased from aaBlocks. NcAAs and ncMs **9**, **10**, **12** and **S3** were purchased from Merck. NcMs **13**, **15**, **16**, **17**, **18**, **20** **and S7** were purchased from BLD. NcM **S2** was purchased from Advanced ChemBlock. NcM **19** and **S6** were purchased from AstaTech. NcAAs and ncMs **3** and **S8** were synthesized as previously described^3^, and ncAA **11** was custom synthesized as previously described^54^. NcMs **S1** and **S5** were Boc-deprotected in concentrated HCl in dioxane.

### DNA oligonucleotides

See Supplementary Data 2.

### DNA constructs

See Supplementary Data 2 for all constructs.

### DNA construct cloning

Standard cloning was performed by Gibson assembly using NEBuilder HiFi DNA Assembly Master Mix (NEB) according to manufacturer’s guidelines. Libraries were generated by enzymatic inverse PCR, as previously described^55,56^. In brief, a template plasmid was amplified by PCR using two primers (see primer list) containing degenerate codons at desired mutagenesis sites and a BsaI cleavage site. In the case of custom mixes, primers containing different codons were manually mixed and used for PCR reactions. PCR products were gel purified and digested using BsaI and DpnI. Subsequently, samples were purified, ligated using T4 DNA Ligase, and transformed into electrocompetent *E. coli* DH10β cells ensuring a minimal transformation efficiency of 10^9^. Individual colonies (>10) were evaluated using Sanger sequencing for quality control of the library assembly. Total plasmid DNA was prepared from the resulting culture, sequenced as a bulk using Sanger sequencing and used for subsequent experiments.

### General protocols

#### Isolation and oxidation of tRNAs (protocol A)

This protocol was used to isolate tRNAs from 1–10 ml of cell culture. Chemically competent DH10β cells were transformed with a pMB1 plasmid encoding a PylRS variant and a tRNA, or a circularly permutated split tRNA, and rescued in 1 ml of SOC for 1 h at 37 °C, 700–1,000 rpm. Cells were transferred into selective 2xYT-s medium and grown overnight. Overnight cultures were diluted in a ratio between 1:20 and 1:40 and grown to OD_600_ of 0.5–1. Cells were centrifuged at 4,200 rcf at 4 °C for 12 min, taken up in 200 µl resuspension buffer, transferred to a 96-well plate and centrifuged at 4,200 rcf at 4 °C for 12 min. Cells were resuspended in 135 µl resuspension buffer and 15 µl liquid phenol was added. Cells were lysed by shaking at 650 rpm for 20 min, and then centrifuged at 4,200 rcf at 4 °C for 20 min; the cell lysate was added to 40 µl chloroform, and the resulting suspension mixed by pipetting up and down. The mixture was centrifuged at 4,200 rcf at 4 °C for 10 min, and 115 µl of the aqueous layer were transferred into 6 µl 0.1 M NaIO_4_. The isolated RNA was oxidized for 1 h on ice, and the oxidation reaction was quenched by addition of 8 µl of 0.1 M DTT. tRNAs were purified using the ZR-96 Oligo Clean & Concentrator from Zymo Research. In brief, 250 µl oligo binding buffer was added to the oxidation reaction, subsequently 400 µl isopropanol was added, and the mixture transferred to a 96-well silica column plate. The plate was centrifuged for 2 min, 4,200 rcf at room temperature, and 800 µl of oligo wash buffer was added. The plate was centrifuged for 2 min, 4,200 rcf at room temperature, aerated, and centrifuged for another 4 min, 4,200 rcf at room temperature. Finally, the RNA was eluted in either 14 µl water, when the samples were not processed, or in 50 µl water, when the samples were further deacylated, by centrifugation for 4 min, 4,200 rcf at room temperature.

#### Isolation and oxidation of tRNAs (protocol B)

The volumes described in this protocol were used to isolate tRNAs from 5–25 ml of cell culture as previously described^22,57^. In brief, cells were grown as described in protocol A, washed with 800 µl resuspension buffer and transferred to a 1.5 ml Eppendorf tube. Cells were taken up in 225 µl resuspension buffer and 25 µl of liquid phenol was added. Cells were lysed by vortexing for one minute and incubation, with head-over-tail rotation, for 20 min. Lysed cells were centrifuged for 15 min, 20,000 rcf at room temperature, the cell lysate was added to 250 µl chloroform, the samples were vortexed for one minute and then centrifuged, 10 min, 20,000 rcf at room temperature. Two-hundred microlitres of the aqueous layer was transferred into 10 µl 0.1 M NaIO_4_ and the RNA was oxidized for 1 h on ice. Finally, the oxidized RNA was added to 440 µl ethanol and precipitated for at least 20 min at −20 °C. The samples were centrifuged for 25 min, 20,000 rcf at 4 °C and aspirated. RNA pellets were dried for 10 min at room temperature and dissolved in water or buffer D.

#### tRNA deacylation

45 μl of isolated RNA was added to 5 µl 10 x deacylation buffer and tRNAs were deacylated for 36 min at 42 °C. The deacylation reaction was quenched by addition of 6 µl 3 M sodium acetate, and tRNAs were purified using the ZR-96 Oligo Clean & Concentrator from Zymo Research, as described in protocol A for the isolation and oxidation of tRNAs (with the exception of using 100 µl of oligo binding buffer, instead of 250 µl). Deacylated tRNAs were eluted in 14 µl water.

#### Fluoro-tREX

This protocol was used to run the experiments shown in Fig. 2c and Supplementary Fig. 4. RNA concentrations were adjusted to the lowest common denominator and 10 µl of RNA was added to 2.5 µl 10x hybridization buffer, 11.5 µl water and 1 µl Cy3-labelled extension primer (2 µM; note that probes for tREX-based approaches are described as primers throughout the methods even though they template extension and are not themselves extended). The DNA primer was hybridized at 65 °C for 5 min, before addition of 25 µl KMM–Cy5 and extension at 37 °C for 6 min. Samples were purified using the 10 µg NEB Monarch RNA clean-up Kit (NEB) and eluted in 12 µl water. 12 µl of orange loading dye was added, the samples were loaded onto a Novex TBE 6 M urea 10 or 15% PAGE gel (Invitrogen) and run for 36 min in 0.5x Tris-borate-EDTA (TBE) buffer at 270 V. Gels were imaged on an Amersham Typhoon Biomolecular Imager (GE) using the Cy3 and Cy5 emission filters. Then gels were stained with SYBR Gold (Invitrogen) and imaged again using the same filters.

#### Mini-fluoro-tREX

Unless stated otherwise, all fluoro-tREX experiments were run with the mini-fluoro-tREX protocol. Six microlitres of RNA was added to 0.5 µl 10x hybridization buffer, and 0.5 µl Cy3-labelled extension primer (2 µM). The DNA primer was hybridized at 65 °C for 5 min, before addition of 5 µl KMM–Cy5-mini and extension at 37 °C for 6 min. Samples were analysed as described for fluoro-tREX.

#### Mini-tREX

This protocol was adapted from Cervettini et al.^22^. Total tRNA was isolated as described in protocol A and deacylated. One to two micrograms of RNA was diluted into a total volume of 6 µl and added to 0.5 µl 10x hybridization buffer, and 0.5 µl Cy5-labelled extension primer (2 µM). The DNA primer was hybridized at 65 °C for 5 min, before addition of 5 µl KMM-mini and extension at 37 °C for 6 min. Samples were analysed by running a 15% acrylamide 1x TBE gel (running 200 V, 40–80 min).

#### Bio–tREX

RNA concentrations were adjusted to match the lowest concentration in the samples being compared. Ten microlitres of RNA was added to 2.5 µl 10x hybridization buffer, 11.5 µl water and 1 µl extension primer (2 µM). The DNA probe was hybridized at 65 °C for 5 min, before addition of 25 µl KMM-bio and extension at 37 °C for 6 min. 10 µl of Dynabeads MyOne Streptavidin C1 magnetic beads (Invitrogen) per reaction were washed 3 times with 200 µl washing buffer, resuspended in 50 µl binding buffer; the beads were added to the extension reaction and binding was performed for at least 30 min at 4 °C, with head-over-tail rotation. The supernatant was removed, and the beads were washed 4 times with 200 µl washing buffer. The washed beads were resuspended in 10 µl FLB and heated to 98 °C for 3 min to release the tRNAs. Beads were removed and the supernatant was directly loaded onto a Novex TBE 6 M urea, 10% or 15% PAGE gel (Invitrogen) and run for 36 min in 0.5x TBE at 270 V. Gels were stained with SYBR Gold (Invitrogen) and imaged on an Amersham Typhoon Biomolecular Imager (GE) using the Cy2 emission filter.

#### Northern blotting

tRNAs were isolated following the general protocol A or B, omitting the oxidation by NaIO_4_. Two to three micrograms of RNA was loaded onto an acidic urea PAGE gel (9% acrylamide (19:1), 100 mM sodium acetate pH 5, 8 M urea) and the gel was run for 12–16 hours, using 100 mM sodium acetate as running buffer, at 6 W constant power. The gel was stained with SYBR Gold (Invitrogen) to identify the tRNAs and an appropriate section of the gel was cut and blotted using iBlot DNA Transfer Stack (Invitrogen) with the iBlot Dry Blotting System. The tRNAs were cross-linked to the membrane (Stratalinker UV Crosslinker 2400), which was subsequently blocked in Ambion ULTRAhyb-Oligo buffer (Invitrogen) for 30 min. The biotinylated DNA probe was added to a final concentration of 0.2 µg ml^−1^ and hybridized overnight at 37 °C and 160 rpm. The membrane was washed 3 times with 20 ml 0.5x TBE buffer and transferred into 15 ml Odyssey blocking buffer for 20 min before addition of IRDye 800CW Streptavidin (LI-COR) to a final concentration of 0.2 µg ml^−1^. Finally, the membrane was washed 3 times with 20 ml 0.5x TBE and imaged on an Amersham Typhoon Biomolecular Imager (GE) using the IR long range emission filter.

#### mRNA extraction and oxidation (A)

The volumes given are suited for 2 to 3 ml of cell culture and were adjusted proportionally when required. Chemically competent BL21 cells were transformed with a pColE1 plasmid encoding the stmRNA construct, which was under the control of a T7 promoter and T7 terminator, rescued in SOC, shaken at 220 rpm, 37 °C for 1 h, diluted into 2xYT-am and grown overnight. The overnight cultures were diluted in a ratio of 1:20 into 2xYT-am in absence or presence of the ncM and grown at 37 °C, 220 rpm to an OD_600_ of 0.5–0.8. PylRS production was induced by addition of Isopropyl β-d-1-thiogalactopyranoside (IPTG) to a final concentration of 1 mM and cells were grown for 20 min, 220 rpm at 37 °C.

Cells were centrifuged at 4,200 rcf at 4 °C for 12 min, resuspended in 800 µl resuspension buffer, transferred to a 96-well plate and centrifuged at 4,200 rcf, 4 °C for 12 min. Subsequently, the procedure outlined in the user manual of the Agencourt RNAdvance Cell v2 RNA isolation kit (Beckman) was followed. In brief, 200 µl LBE containing 10 µl proteinase K were used to lyse cells at room temperature for 1 h. 244 μl BBC beads were mixed with 266 µl isopropanol, added to the lysate, and the RNA was bound to the beads for 10 min at room temperature. Beads were washed 3 times with 200 µl 80% ethanol, after the final wash the beads were carefully dried, and the RNA was eluted in 80 µl water.

70 μl of the RNA solution was added to 40 µl resuspension buffer and 7.5 µl of 0.1 M NaIO_4_. The oxidation was run on ice for 1 h and quenched with 10.5 µl 100 mM DTT. To the oxidized RNA, a mix of 1.5 µl of 1.6 M Na_2_CO_3_ and 16.5 µl DNAse I buffer (Ambion DNAse I, Thermofisher) was added and the samples were resuspended. Subsequently, 18 µl of DNAse I was added, and the RNA was incubated at 37 °C for 30 min. The digestion was cooled on ice and 300 µl Agencourt RNAClean XP beads (Beckman) were added and the RNA bound for 10 min at room temperature. The beads were washed 3 times with 80% ethanol. After the final wash the beads were carefully dried, and the RNA was eluted in 25 µl water.

#### mRNA extraction and oxidation (B)

A similar protocol to the one outlined in procedure A was followed, but the RNA was isolated using acid phenol/chloroform extraction. In brief, BL21 *E. coli* harbouring stmRNAs were grown in 5 ml of 2xYT-am in the presence or absence of the ncM until an OD_600_ 0.5–0.9. At this point stmRNA expression was induced by addition of 1 M IPTG to a final concentration of 1 mM. After 20 min, cells were collected by centrifugation at 4,200 rcf for 12 min at 4 °C. Cell pellets were resuspended in 800 µl resuspension buffer, transferred into 1.5 ml Eppendorf tubes, and centrifuged for 3 min at 4,200 rcf at room temperature. The supernatant was removed, and the pellets were resuspended in 500 µl SLB. 500 µl of acid-phenol was rapidly added and the tubes were vortexed for 1 min and centrifuged for 6 min, 21,000 rcf at room temperature. 450 μl of the aqueous layer was recovered and 50 µl of 2.5 M KCl was added. Acid-phenol chloroform extraction was repeated, retrieving 400 µl of the aqueous layer. 400 μl of chloroform was added and the samples were vortexed for 1 min followed by centrifugation at 21,000 rcf 6 min at room temperature. 300 μl of the aqueous layer was recovered, and chloroform extraction was repeated with 300 µl chloroform. 200 µl of the aqueous phase were transferred to new 1.5 ml Eppendorf tubes and 10 µl of 0.1 M NaIO_4_ was added. The oxidation reaction was run for 1 h on ice. 440 μl of ethanol was added and RNA was precipitated at −20 °C for at least 20 min. The RNA was pelleted by centrifugation at 21,000 rcf at 4 °C for 30 min. The supernatant was removed, and the pellets air-dried for 10 min. The RNA was resuspended in 50 µl water. 30 μl of each RNA sample was digested in 120 µl 1x Ambion DNAse I buffer including 12 µl Ambion DNAse I enzyme. Samples were purified with 50 µg Monarch RNA clean-up Kit (NEB) and eluted into 20 µl water. We note, that when the RNA is isolated by acid-phenol chloroform extraction, an additional deacylation step in deacylation buffer is required, to measure acylation of stmRNAs with non-α amino acid monomers.

#### Fluoro-mREX

RNA concentration for all samples were adjusted to match the lowest concentration in the samples being compared. Six to twelve micrograms of RNA was added to a mixture of 1 µl DNA probe (2 µM), 2.5 µl 10x hybridization buffer and water (added to a final volume of 25 µl). The primer was annealed at 65 °C for 5 min. 25 µl KMM–Cy5 was added. The primer was extended for 6 min, 37 °C. Samples were purified using the 10 µg NEB Monarch RNA clean-up Kit (*NEB*) and eluted in 12 µl water. 12 μl of orange loading dye was added to each sample. Gel electrophoresis was conducted using 1% agarose gels (cast using NorthernMax MOPS running buffer) in NorthernMax MOPS running buffer at 135 V, 42 min. Gels were stained with SYBR Gold (Invitrogen) and imaged on an Amersham Typhoon Biomolecular Imager (GE) using the Cy2 and Cy5 emission filter.

#### Bio–mREX

The RNA concentration of all samples, after RNA extraction and oxidation (Methods), was adjusted to match the lowest concentration in the samples being compared.

To perform the pulldown, 6–12 µg of RNA was added to a mixture of 1 µl DNA probe (2 µM), 2.5 µl 10x hybridization buffer and water (added to a final volume of 25 µl). The primer was annealed at 65 °C for 5 min. 25 µl KMM-bio were added. The primer was extended for 6 min, 37 °C. Ten microlitres of Dynabeads MyOne *C1* streptavidin beads (Invitrogen) were washed 2 times with washing buffer, and resuspended in 50 µl binding buffer. Resuspended beads were added to the extension reaction, and the biotinylated stmRNAs were bound to the beads for 1 h, 4 °C, with head-over-tail rotation. The beads were washed on a magnetic stand with 3 × 200 µl washing buffer, two times 200 µl AWB, one time 200 µl washing buffer, one time 200 µl water and were finally resuspended in 13 µl of RHM. After the AWB wash and after the final wash with washing buffer, the beads were transferred into new plastic tubes. The primer was annealed at 65 °C for 5 min. 7 µl of RMM was added and the RNA reverse transcribed at 50 °C for 10 min. One microlitre of RNAse H was added and the mixture heated to 37 °C for 15 min and 98 °C for 3 min to release the cDNA from the beads. Finally, the cDNA was separated from the beads and either used for quantification by qPCR, NGS, or as a template for further cloning.

To determine the number of molecules in the input for the stmRNA pulldown, extracted and oxidized RNA samples, in 13 µl of RHM, were reverse transcribed using the same procedure as for the pulled-down stmRNA. The percentage of the input stmRNA molecules recovered in the pulldown was determined from the number of molecules before and after the pulldown, as determined by qPCR ((number of molecules after pulldown/number of molecules in input) × 100).

#### qPCR of cDNA from bio–mREX

qPCR reactions were run in triplicate for each bio–mREX sample and were composed of 2 µl of cDNA, 10 µl PowerUp SYBR Green Master Mix (Applied Biosystems), 0.4 µl of each primer and 7.2 µl water. A standard was generated by PCR of the *Mm*PylRS gene and quantified using a Qubit 2 Fluorometer (Life Technologies) and the Qubit 1x dsDNA HS Assay Kit (Invitrogen). A five-step fivefold serial dilution was used to generate a qPCR standard curve. This allowed calculation of qPCR efficiency and the number of molecules in each sample. qPCR was run on a ViiA 7 Real-Time PCR System (Applied Biosystems) using the standard supplier protocol for SYBR Green (Invitrogen).

#### Preparation of cDNA from bio–tREX for NGS

Half of the cDNA from the 20 µl reverse transcription reaction from bio–tREX was added into a PCR mix containing 25 µl Q5 High-Fidelity 2x Master Mix, 12 µl water and 2 µl of a 10 µM predefined mix of indexing primers (see Supplementary Data 2). A standard PCR program with 29 amplification cycles and an annealing temperature of 60 °C was used. Extension times were adapted to the amplicon length according to the manufacturer’s guidelines. DNA was bound to 100 µl of Agencourt AMPure XP (Beckman) for 10 min and the beads were washed 3 times with 200 µl 80% ethanol. Beads were dried and DNA was eluted in 25 µl water. DNA concentrations were measured using Qubit 2 Fluorometer (Life Technologies) and the Qubit 1x dsDNA HS Assay Kit (Invitrogen) and 80 ng of each amplicon were combined into the NGS library. The combined library was diluted in HT1 Hybridization Buffer (Illumina) to a concentration of 2 nM. PhiX (Illumina) was added to increase the diversity of the library at a 20% molar ratio. 12 µl of the library was added to 18 µl HT1 Hybridization Buffer (Illumina) and 20 µl of the diluted mixture was used for NGS analysis.

#### Cloning of cDNA from bio–tREX for further evolution

Half of the cDNA from the 20-µl reverse transcription reaction, from bio–tREX, was added into a PCR mix containing 25 µl Q5 High-Fidelity 2x Master Mix, 12 µl water and 2 µl of a 10 µM predefined mix of golden gate assembly primers (see Supplementary Data 2). A touchdown PCR program was used. The initial annealing temperature of 65 °C was decreased over 10 cycles by 0.5 °C per cycle. Subsequently, 20 regular cycles using an annealing temperature of 58 °C were performed. Extension times were adapted to the amplicon length according to the manufacturer’s guidelines. DNA was bound to 100 µl of Agencourt AMPure XP (Beckman) for 10 min and the beads were washed 3 times with 200 µl 80% ethanol. Beads were dried and DNA was eluted in 25 µl water. The amplicon was then cloned into a new pColE1 backbone, which was previously amplified by Golden Gate primers (Supplementary Data 2), by two-piece Golden Gate assembly according to New England Biolabs’ guidelines.

#### NGS data analysis

NGS was performed on a MiSeq system (in the case of the test evolution with library 1 and substrate **2**) or a NextSeq2000 system (in all other cases). The resulting cDNA from tRNA display was amplified using oligos NGS A(1-8) and NGS_B(1-8) (Supplementary Data 2) containing different combination of Nextera sequencing barcodes via PCR. Samples were purified, quantified, and combined in equimolar amounts. Paired end reads were first paired using PEAR^58^, and aligned to a reference sequence of *Mm*PylRS using Bowtie2^59^. The relevant library positions were extracted and translated to amino acids, and resulting variants were counted using R script. Subsequent operations were performed using the frequency of each variant in each library which was computed as the count value divided by the total number of counts of that library. Using R script, enrichment and selectivity scores were calculated for all variants as follows. First, variants that were only present in all positive replicates were considered (tables were merged using AND operator). Assuming that highly enriched sequences could potentially not be covered in the negative and the input samples but may still be of interest, the negative and the naïve replicates were merged to the positive table using an OR operator. A placeholder value of 0.95 counts was adopted in cases where a replicate did not cover a specific variant. The resulting dataset was used to calculate mean enrichments in the presence and in the absence of the ncAA or ncM, computed as the quotient of the mean frequencies in one condition and the input condition. The resulting positive and negative enrichments were used to calculate the selectivity value for each variant (equivalent to the quotient of positive and negative frequencies). For further analysis, variants were filtered using an empirically determined threshold value for the normalized standard deviation of the positive frequency (dispersion error in the plus substrate condition).

#### tRNA pulldown and ncM identification by LC–MS

tRNAs were isolated from 8 ml of cells following the general protocol B omitting the oxidation by NaIO_4_. The RNA pellet was resuspended in 90 µl buffer D and RNA concentrations adjusted to match the lowest concentration in the samples being compared. A volume of 0.5 µl of biotinylated DNA probe (100 µM) was added to the RNA and the DNA probe was hybridized at 65 °C for 5 min. 40 μl of Streptavidin Dynabeads MyOne C1 (Invitrogen), were washed twice with buffer D-T (buffer D with 0.05% (v/v) Tween), and added into 10 µl buffer D. The beads were added to hybridization reaction and the probe was bound to the beads for minimally 30 min at 4 °C with head-over-tail rotation. The samples were washed 3 times with 200 µl of acW1-T, twice with 200 µl of acW1, 3 times with 200 µl of acW2 and once with 200 µl water, all on a magnetic stand. 24 µl of deacylation buffer was added and the beads, which were incubated at 42 °C for one hour. 12 µl of the deacylation mix was added to 3 µl 6-aminoquinolyl-*N*-hydroxysuccinimidyl carbamate (AQC, 3 mg ml^−1^ in acetonitrile) and the reaction incubated at 55 °C for 15 min. Samples were analysed on an Agilent Technologies 6130 Quadrupole LC–MS using single ion monitoring.

#### GFP(150X)–His_6_ and GFP(3X)–His_6_ fluorescence assay

Chemically competent DH10β cells harbouring a p15A plasmid encoding GFP(150TAG)His_6_ or GFP(3TAG)His_6_ (two versions of the p15A plasmid with either a tetracycline or apramycin resistance cassette, which led to similar results, were used interchangeably) were transformed with a pMB1 plasmid encoding *Mm*PylRS, or a mutant thereof, and *M. mazei* ${{\rm{tRNA}}}_{{\rm{C}}{\rm{U}}{\rm{A}}}^{{\rm{P}}{\rm{y}}{\rm{l}}}$, rescued in SOC and grown overnight in 2xYTs-t or 2xYTs-ap. 20 μl of the overnight culture was diluted into 480 µl 2xYTs-t or 2xYTs-ap containing 0.2 l-arabinose in presence and absence of 2 to 4 mM of the respective ncM in a 96-well-plate format. Cells were grown for 16–20 h at 37 °C at 700 rpm The plates were centrifuged for 12 min, 4,200 rcf at 4 °C and the cells resuspended in 150 µl PBS. One-hundred microlitres of the resuspended cells were transferred into a Costar 96-well flat bottom plate and the OD_600_, and GFP fluorescence was measured using a PHERAstar FS plate reader.

#### GFP(150X)_His6_ isolation for mass spectrometry analysis

Three replicates of the protein produced as described above were combined in a 1.5 ml Eppendorf tube, centrifuged at 4,200 rcf for 3 min, frozen at −20 °C, thawed and resuspended in 150 µl BugBuster (Millipore). Cells were lysed for 1 h with head-over-tail rotation. Lysed cells were centrifuged for 20 min, 20 000 rcf, at 4 °C and the lysate was added to 20 µl of Ni-NTA beads. GFP(150X)–His_6_ was bound to the beads for 20 min at room temperature with head-over-tail rotation. The beads were washed 6 times with 60 µl 30 mM imidazole in PBS, and the protein was eluted with 5 times 30 µl 300 mM imidazole in PBS.

For lower-activity mutants for ncM **13**, 5–15 ml of cell culture was used for protein production. The volumes of BugBuster were adjusted proportionally, all other volumes were kept the same.

#### Mass spectroscopy

ESI–MS and MS/MS were performed as previously described^29,33^.

#### Protein expression, purification and crystallization

Chemically competent DH10β cells harbouring a p15A plasmid encoding GFP(150TAG)His_6_ and a pMB1 plasmid encoding *Mm*PylRS(**13**_1) and *Mm*tRNA^Pyl^ were transformed, rescued in SOC and grown overnight in 2xYTs-ap. 10 ml of the overnight culture was diluted into 1 l 2xYTs-ap containing 0.2% l-arabinose in presence of 5 mM **13**.

Bacterial pellet of 1 l expression culture of GFP(150(S)β^3^mBrF)–His_6_ was lysed by sonication, centrifuged at 142,000 rcf for 30 min and supernatant bound to Ni-NTA beads (Qiagen). Beads were washed three times before protein was eluted and further purified by gel filtration using a Superdex 75 HiLoad 26/60 pg column (GE Healthcare) in 25 mM Tris pH 7.4, 200 mM NaCl and 0.06% NaN_3_. The purified protein was concentrated using Vivaspin 20, 10,000 MWCO (Sartorius) to 6 mg ml^−1^. Sample was Trypsin digested with Sequencing Grade Modified Trypsin (Promega) in a 50:1 ratio. Sample was incubated for 1 h at 37 °C, centrifuged at 21,000 rcf for 10 min before plating in crystal trays. Crystallization trials with multiple commercial crystallization kits were performed in 96-well sitting-drop vapour diffusion plates (Molecular Dimensions) at 18 °C and set up with a Mosquito HTS robot (TTP Labtech). Drop ratios of 0.2 μl protein solution plus 0.2 μl reservoir solution were used for screening. The only useful dataset was collected from a crystal collected from the Fusion screen (Molecular Dimensions)^60^ with following composition: 37.5% PEG 3350/PEG 1 K/MPD (1:1:1), 0.1 M Bicine/Trizma pH 8.5, 0.8% (w/v) Morpheus III Alkaloids and 0.12 M Morpheus Alcohols. Crystals were collected and flash frozen in liquid nitrogen.

#### Diffraction data collection, processing and structure solution

Diffraction data were collected at the ESRF on beamline ID23-2 at an energy of 14.2 keV. Data were processed with XDS^61^ via the pipeline autoProc (Global Phasing)^62^. The structure was solved by molecular replacement with MolRep^63^ using the homologue model PDB 2B3P. Iterative building was performed with Coot^64^, refinement with REFMAC5^65^, and validation with Molprobity^66^. Figures of the structure were prepared with PyMOL (PyMOL Molecular Graphics System, Schrödinger).

#### Selection for ncAAs

The selection was performed as described in Supplementary Fig. 11. RNA was isolated and oxidized as described in general procedure A. Bio–mREX was performed as specified in the general procedure. After the first round of selection, the new libraries were assembled from the amplified cDNA as described above. After the second round of selection the NGS samples were prepared from the isolated cDNA as described above, the NGS was run using a P2 600 cycles cartridge, and the data were analysed as specified above.

#### Selection for ncMs

The selections were performed as described in Supplementary Figs. 22 and 35. RNA was isolated and oxidized as described in general procedure A. Bio–mREX was performed as specified in the general procedure. The NGS samples were prepared from the isolated cDNA as described above, the NGS run using a P1 600 cycles cartridge, and the data were analysed as specified above.

#### Selection for substrate 13 using a random mutagenesis library

The concentrations of the pMB1 plasmids encoding PylRS hits **13_1** and **13_2** were measured by Qubit 2 Fluorometer (Life Technologies) and the Qubit 1x dsDNA HS Assay Kit (Invitrogen) and the plasmids combined in equimolar amounts. The combined plasmids were used for an error prone PCR of the active site of PylRS using golden gate primers (Supplementary Data 2) and the GeneMorph II kit (Agilent) at conditions leading to the maximal number of random mutations. The amplicons were cloned into a new pColE1 backbone by two-piece Golden Gate assembly according to NEB (New England Biolabs) guidelines. The selection was performed as outlined in Supplementary Fig. 33. RNA was isolated and oxidized as described in general procedure A. Bio–mREX was performed as specified in the general procedure. The NGS samples were prepared from the isolated cDNA as described above, the NGS was run using a P2 600 cycles cartridge, and the data were analysed as specified above.

#### Statistics

Graphpad Prism version 9 was used to generate all bar graphs in this study. For sequence alignments and further processing of the NGS data custom R scripts were used.

### Reporting summary

Further information on research design is available in the Nature Portfolio Reporting Summary linked to this article.

## Online content

Any methods, additional references, Nature Portfolio reporting summaries, source data, extended data, supplementary information, acknowledgements, peer review information; details of author contributions and competing interests; and statements of data and code availability are available at 10.1038/s41586-023-06897-6.

### Supplementary information

Supplementary Information This file contains Supplementary Note 1, Supplementary Figs. 1–45 and references.

Reporting Summary

Supplementary Data 1 Source data for Figs 4 and 5 and Supplementary Figs 2, 6, 14–22 and 45.

Supplementary Data 2 List of plasmids and oligonucleotides used in this study.

## Figures and Tables

**Extended Data Table 1:**
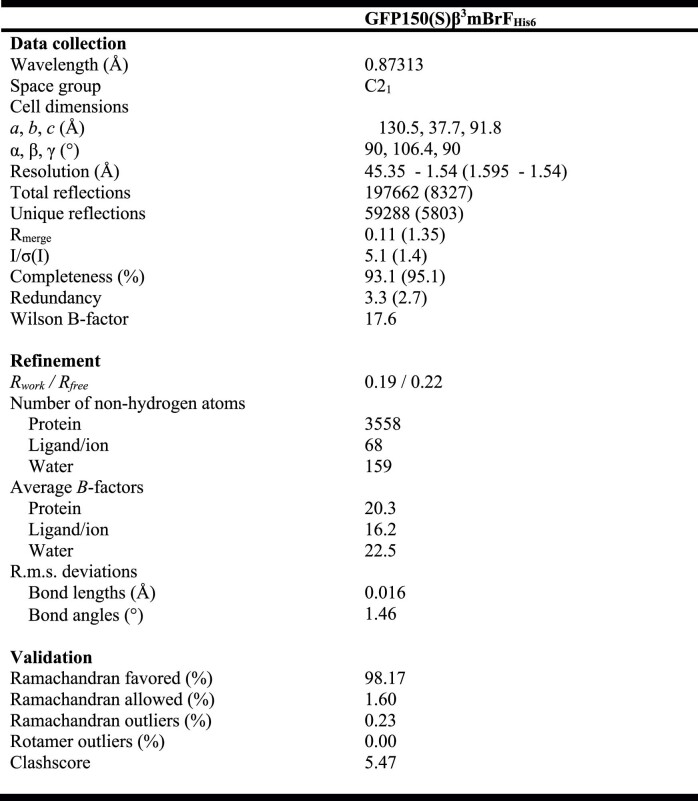
Data collection and validation statistics (Molecular Replacement) for the crystal structure of GFP(150(S)β^3^mBrF)–His_6_

The experiment was executed on a single crystal. Statistics for the highest-resolution shell are shown in parentheses.

## Data Availability

Primary data for main and supplementary figures are available in Supplementary Data 1. A list of plasmids and oligonucleotides used in this study is available in Supplementary Data 2. Sequencing data generated in this study were deposited at the Sequencing Read Archive under the BioProject ID PRJNA1014447. The structure of GFP(150(S)β3mBrF)–His6 is available in the Protein Data Bank under accession code 8OVY. All other datasets generated and/or analysed in this study are available from the corresponding author on reasonable request. All materials (Supplementary Data 2) from this study are available from the corresponding author on reasonable request.

## Code availability

All code used for analysis of tRNA display data is available at GitHub (https://github.com/JWChin-Lab/tRNA-display).
