# Supplementary material for: Adding α,α-disubstituted and β-linked monomers to the genetic code of an organism
Source: Nature. 2024 Jan 10;625(7995):603–10. doi: 10.1038/s41586-023-06897-6 (PMC10794150; doi:10.1038/s41586-023-06897-6)
Supplement: Supplementary file 1 — This file contains Supplementary Note 1, Supplementary Figs. 1–45 and references. [file 41586_2023_6897_MOESM1_ESM.pdf]

nature portfolio

https://doi.org/10.1038/s41586-023-06897-6

Supplementary information

Adding α,α-disubstituted and β-linked monomers to the genetic code of an organism

In the format provided by the authors and unedited

Adding α,α-disubstituted & β-linked monomers to the genetic code of an organism

Daniel L. Dunkelmann[1,2], Carlos Piedrafita[1,2], Alexandre Dickson[1], Kim C. Liu[1], Thomas S. Elliott[1], Marc Fiedler[1], Dom Bellini[1], Andrew Zhou[1], Daniele Cervettini[1], Jason W. Chin[1*]

[1]Medical Research Council Laboratory of Molecular Biology, Francis Crick Avenue, Cambridge, England, UK,

[2] These authors contributed equally

[*]Correspondence: chin@mrc-lmb.cam.ac.uk

Table of Contents

Supplementary Note 1 3

Supplementary Figures 1-45 4

Supplementary Note 1. Optimizing the dynamic range of bio-mREX

To test the dynamic range of bio-mREX, we created stmRNA constructs for a set of four PylRS (CbzK) variants (PylRS mutants that direct the incorporation of CbzK, **2**). When paired with $tRNA^{Pyl}_{CUA}$ and **2**, these PylRS variants lead to GFP fluorescence from *GFP(150TAG)$_{His6}$*, and the level of GFP fluorescence generated by the PylRS variants spans two orders of magnitude (**Fig. 3e**). Assuming that the GFP signal primarily reflects the efficiency with which the PylRS(CbzK) variants aminoacylate their cognate $tRNA^{Pyl}_{CUA}$, we would ideally see a correlation between GFP fluorescence and the number of molecules recovered in bio-mREX (**Extended Data Fig. 3**). However, when assayed *via* bio-mREX, we observed a similarly high recovery of cDNA for the three most active PylRS variants; this suggested that above a threshold activity the first generation of bio-mREX could not effectively differentiate between different acylation activities (**Supplementary Fig. 9, Fig. 3e, Supplementary Data 1**).

The maximum potential dynamic range of bio-mREX will be defined by the number of stmRNAs in a cell, as this defines the number of split tRNA substrates and therefore the number of acylation events that can occur before all stmRNAs in a cell are acylated. We expressed the stmRNAs from a strong *T7* promoter to maximize the stmRNA transcription and therefore the potential dynamic range of bio-mREX. However, in stmRNAs the PylRS mRNA is on the same transcript as the split tRNA substrate; therefore, increasing the transcription of the stmRNA is likely to increase the concentration of the PylRS enzyme. At high PylRS enzyme concentrations, intrinsically less active enzymes may acylate the split tRNA substrate to completion (through mass action) leading to a compression in the dynamic range of bio-mREX. These considerations suggested that high stmRNA levels and low PylRS enzyme levels would maximize the experimental dynamic range of bio-mREX.

The production of PylRS is a function of the abundance of the stmRNA and the efficiency with which the PylRS mRNA, within the stmRNA, is translated. Since translational efficiencies in *E. coli* can be controlled by ribosome binding site (RBS) strength we set out to selectively tune the concentration of the PylRS enzyme through RBS engineering. We designed 5'UTR sequences with attenuated translation rates using a *de novo DNA* RBS calculator and introduced them in to the mRNAs for the PylRS(CbzK) variants within stmRNAs.

Figure 1

Figure 1b

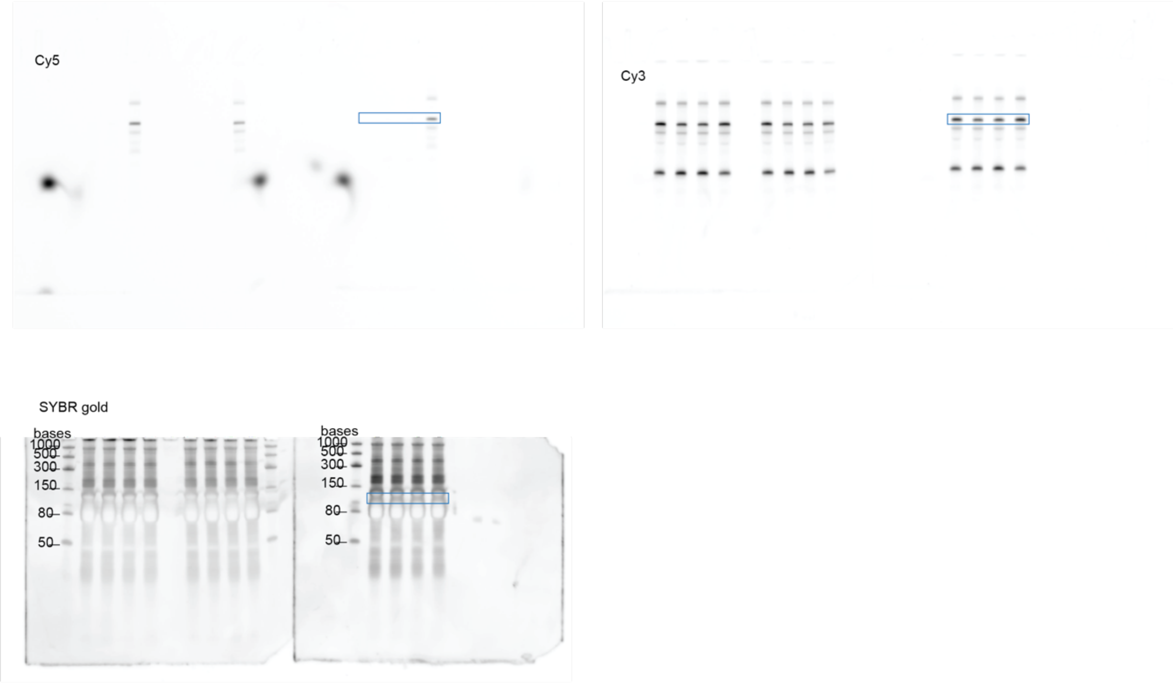

Figure 1c

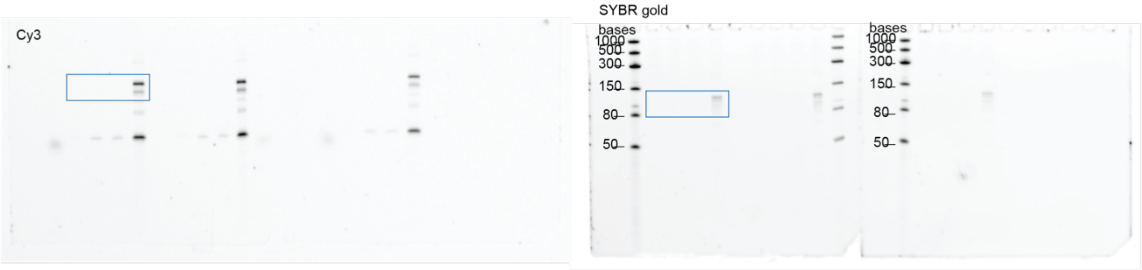

Figure 2

Figure 2c

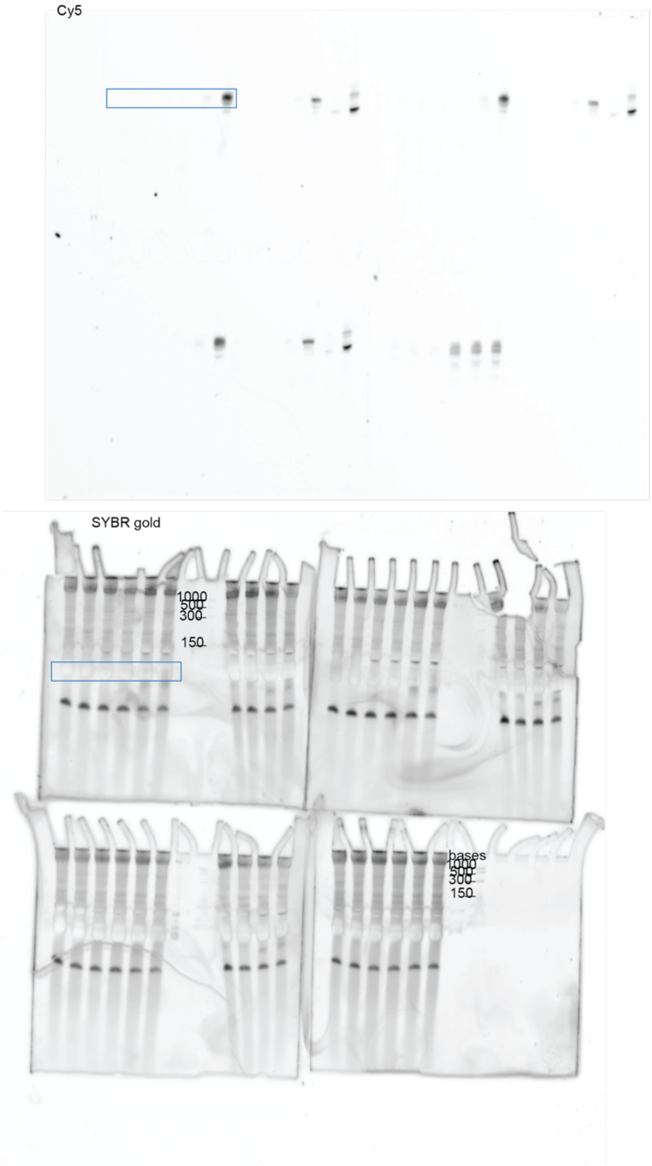

Figure 2d

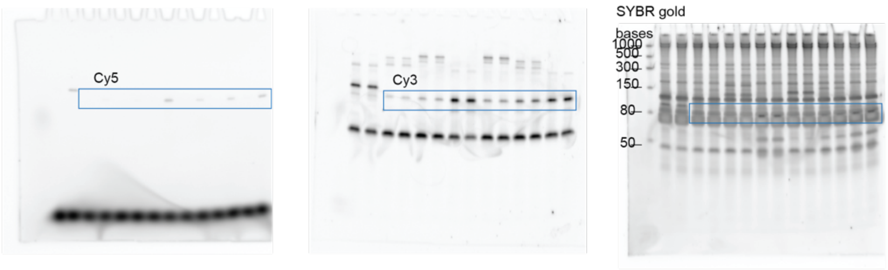

Figure 3

Figure 3b

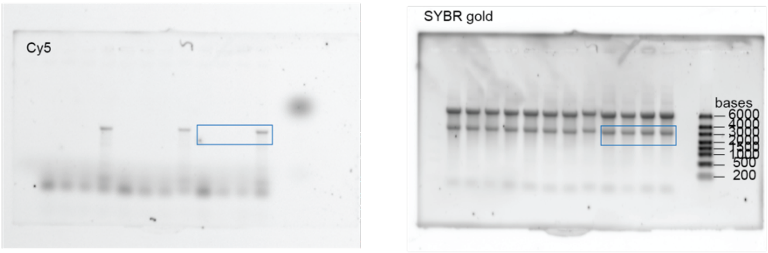

Figure 5

Figure 5b 13_1

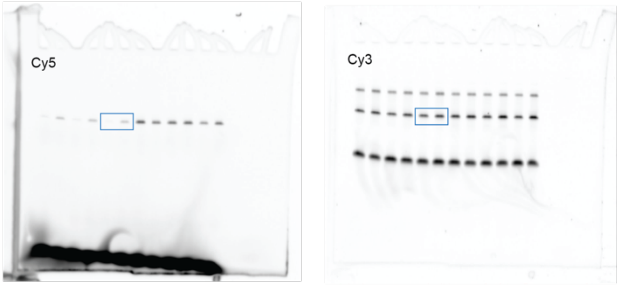

Figure 5b 13_1^{evol1}

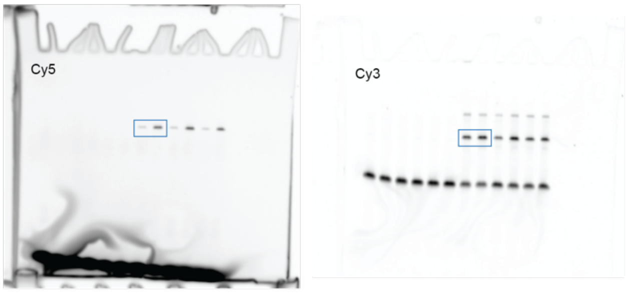

Figure 5d

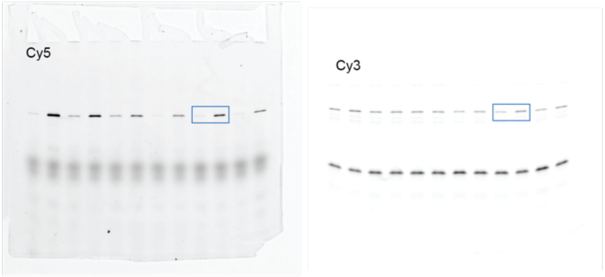

Figure 5f

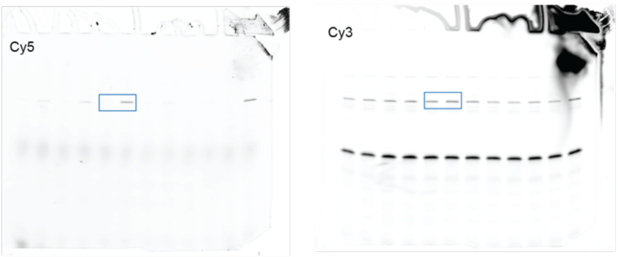

Figure 5h

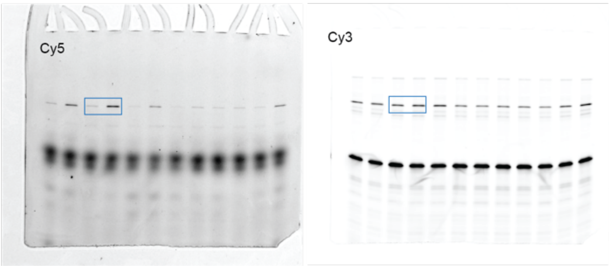

Figure 5j

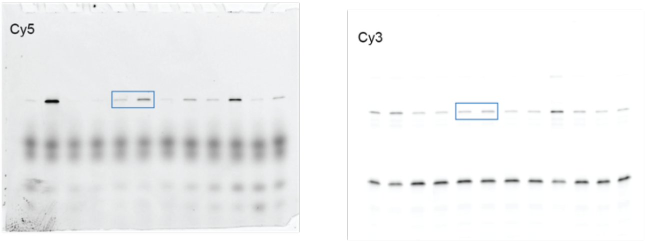

Figure 5l

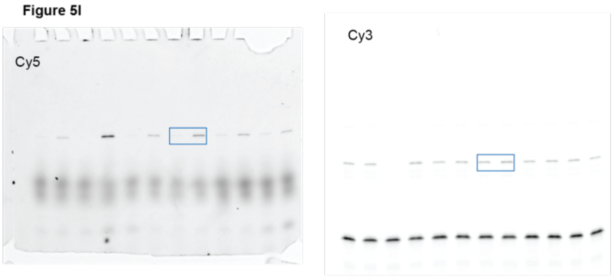

Figure 5n

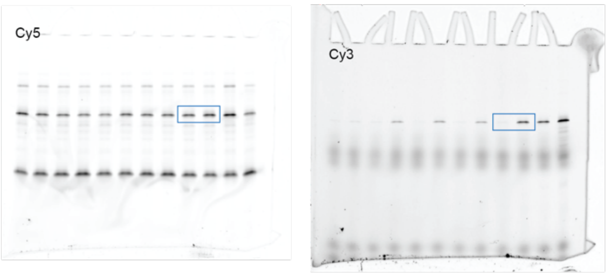

Figure 5p

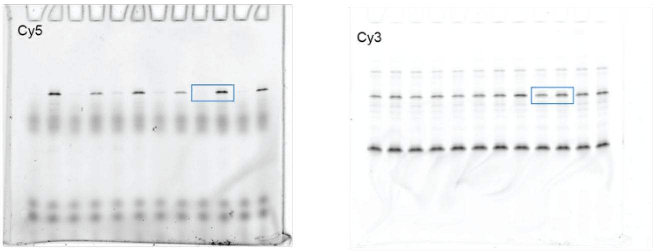

Supplementary Information

SI Figure 2c

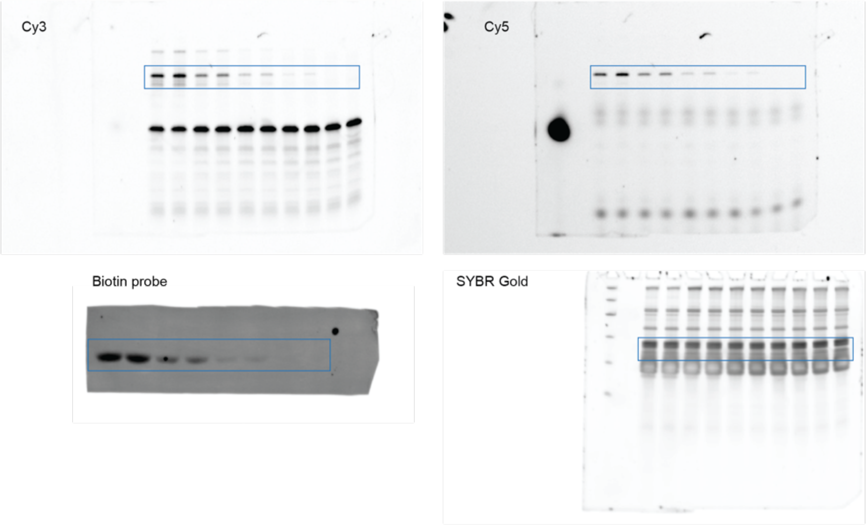

SI Figure 2d

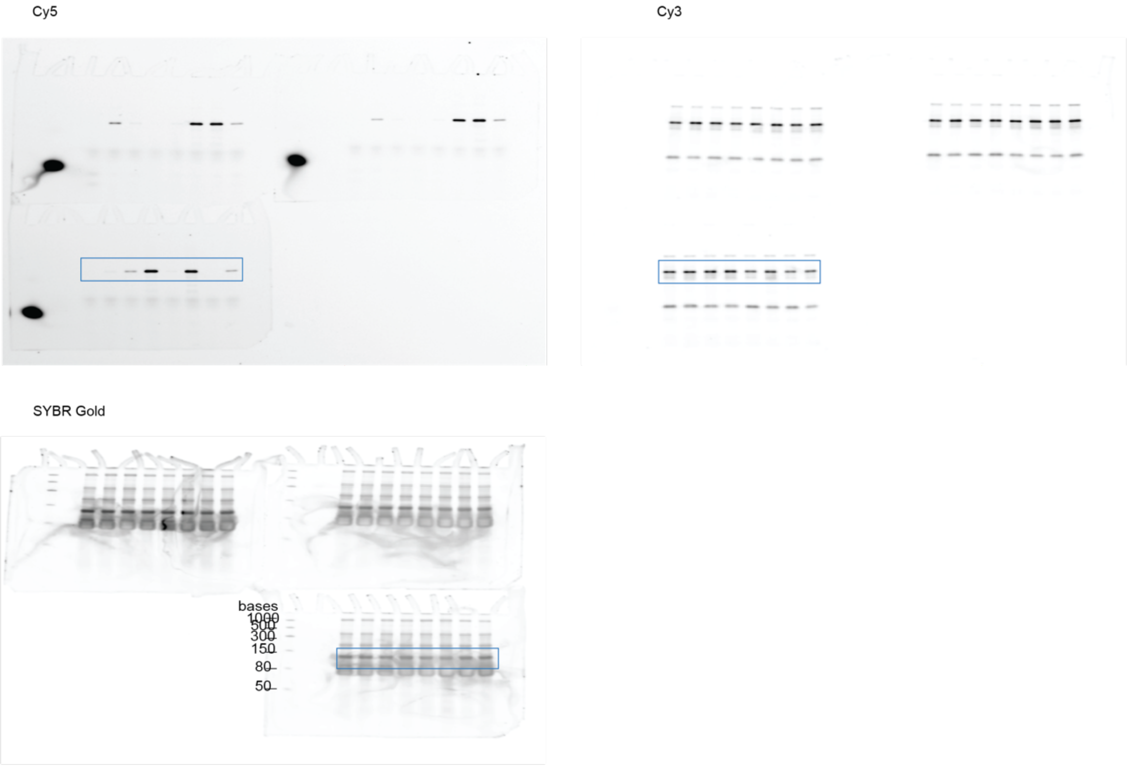

SI Figure 3

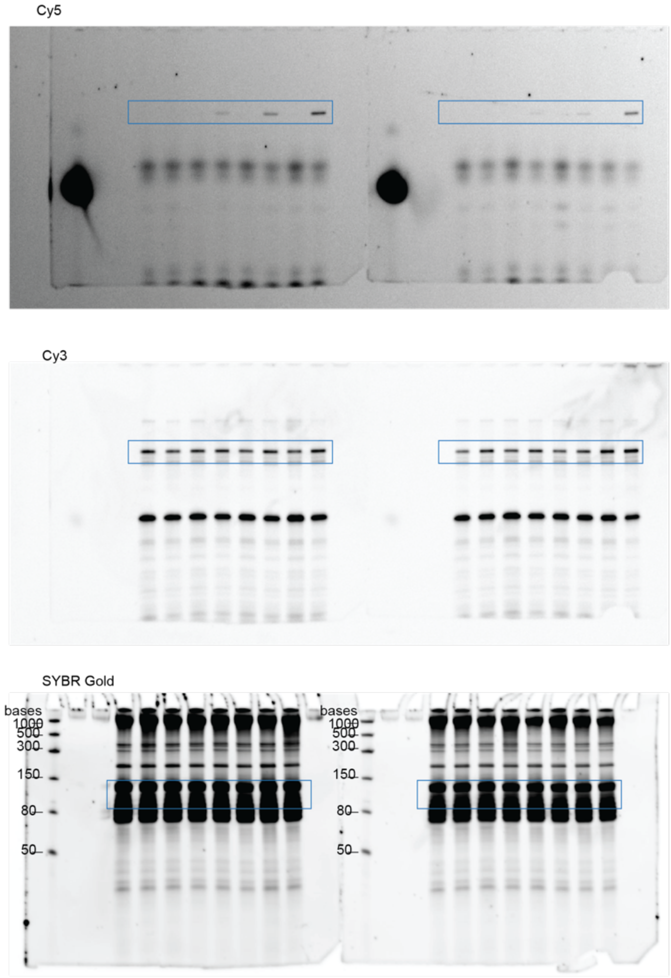

SI Figure 4

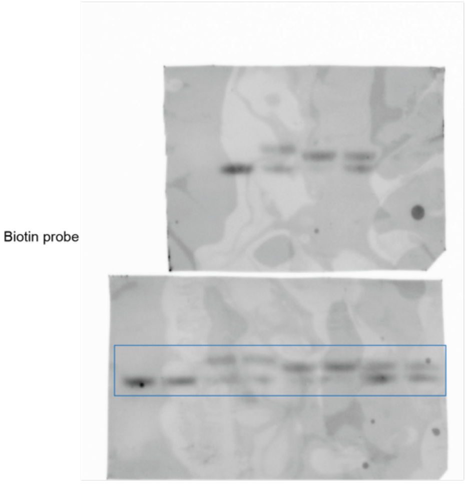

SI Figure 5

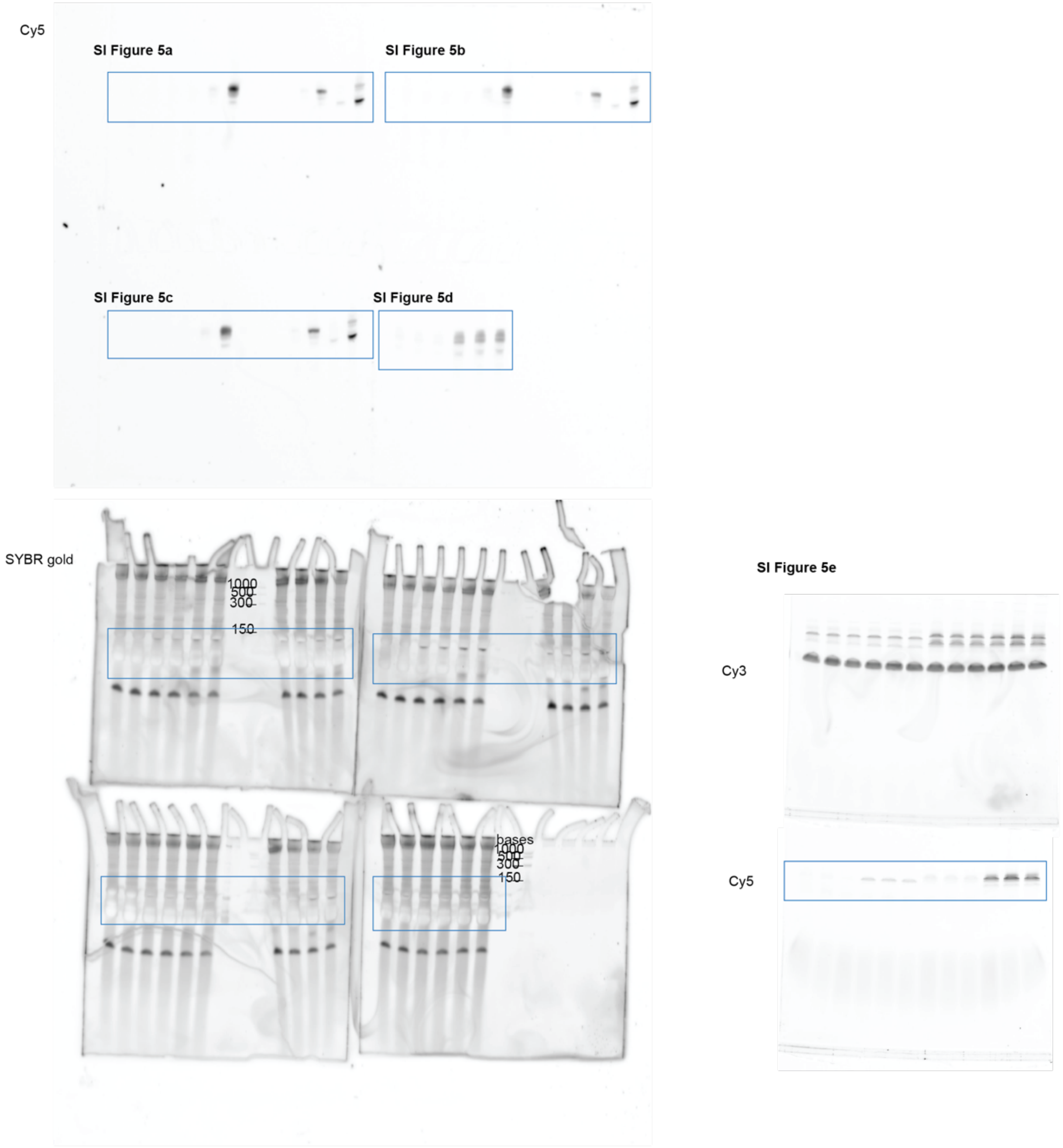
Cy5
SI Figure 5a
SI Figure 5b
SI Figure 5c
SI Figure 5d
SYBR gold
1000
500
300
150
bases
1000
500
300
150
SI Figure 5e
Cy3
Cy5

SI Figure 6

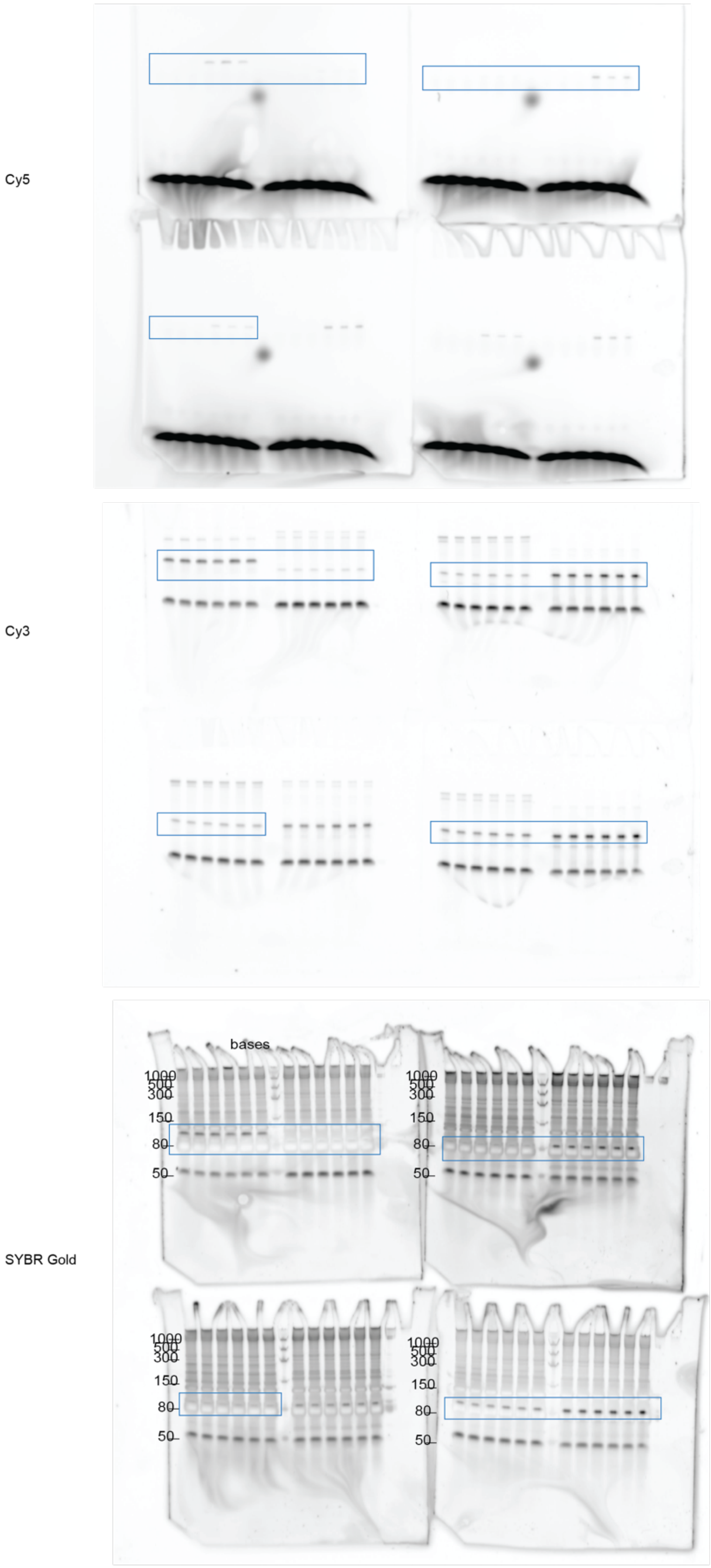

SI Figures 23, 24, 25, 27, 29, 34

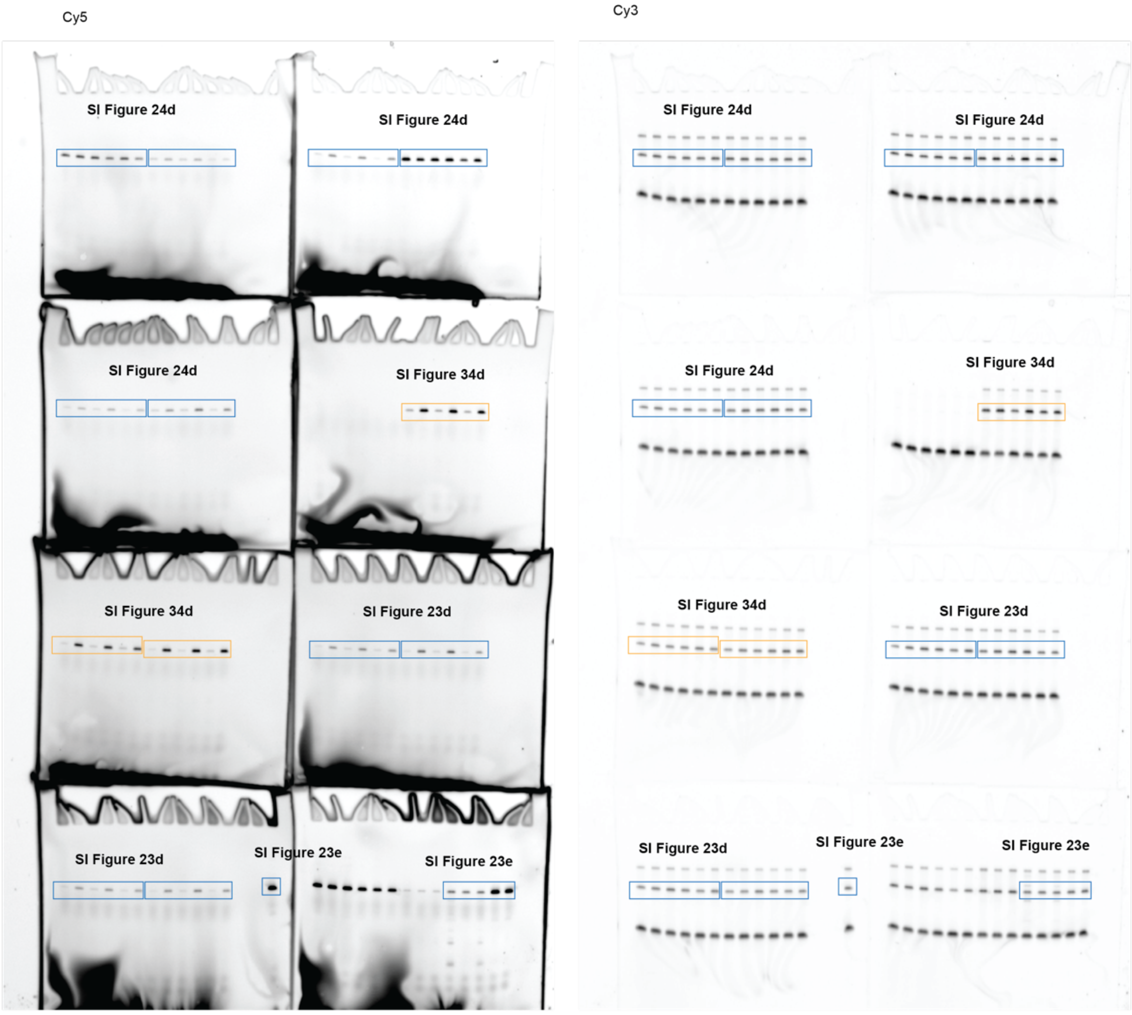

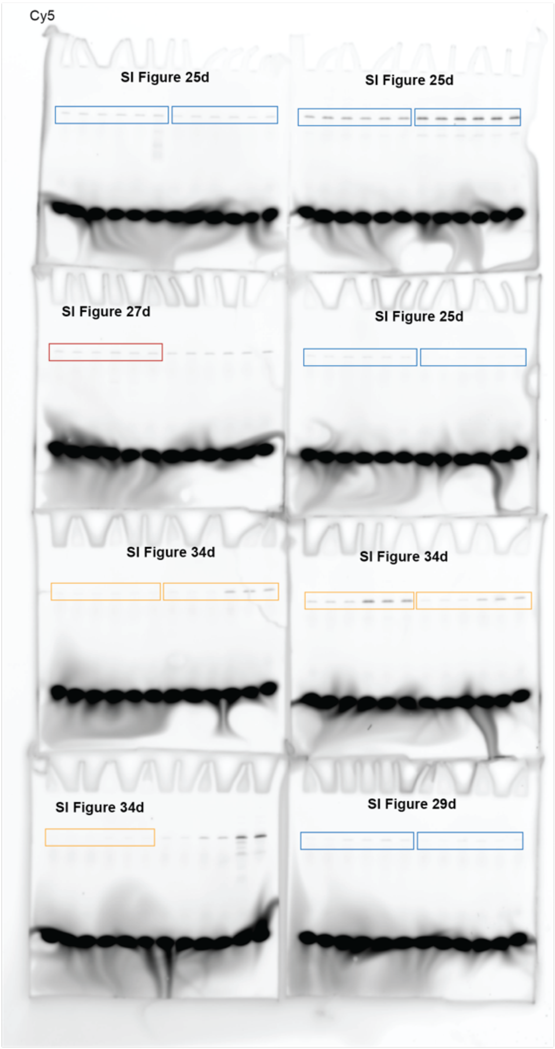
Cy5
SI Figure 25d
SI Figure 25d
SI Figure 27d
SI Figure 25d
SI Figure 34d
SI Figure 34d
SI Figure 34d
SI Figure 29d

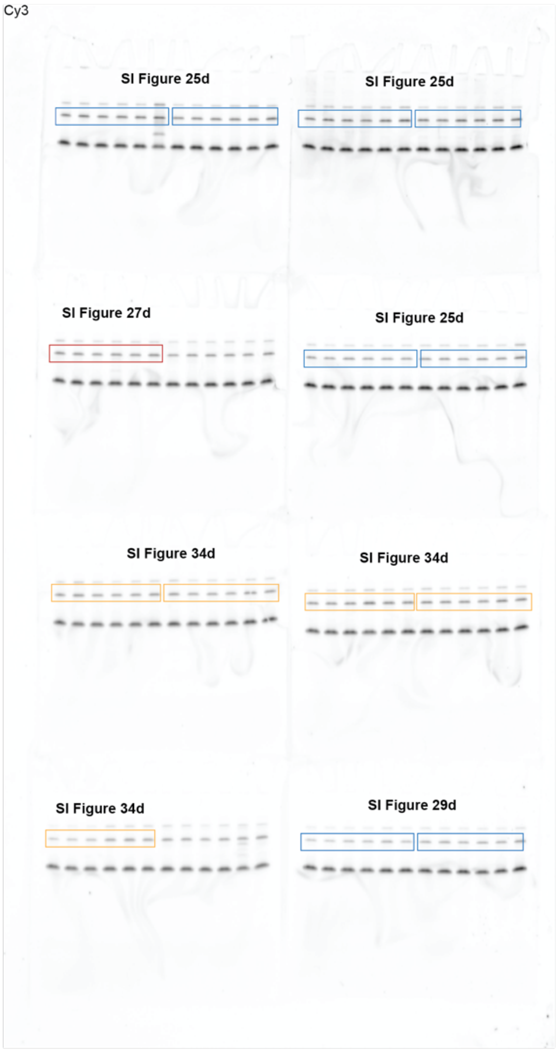
Cy3
SI Figure 25d
SI Figure 25d
SI Figure 27d
SI Figure 25d
SI Figure 34d
SI Figure 34d
SI Figure 34d
SI Figure 29d

SI Figures 36, 37, 38, 39, 40, 41, 42

Cy5 Cy3

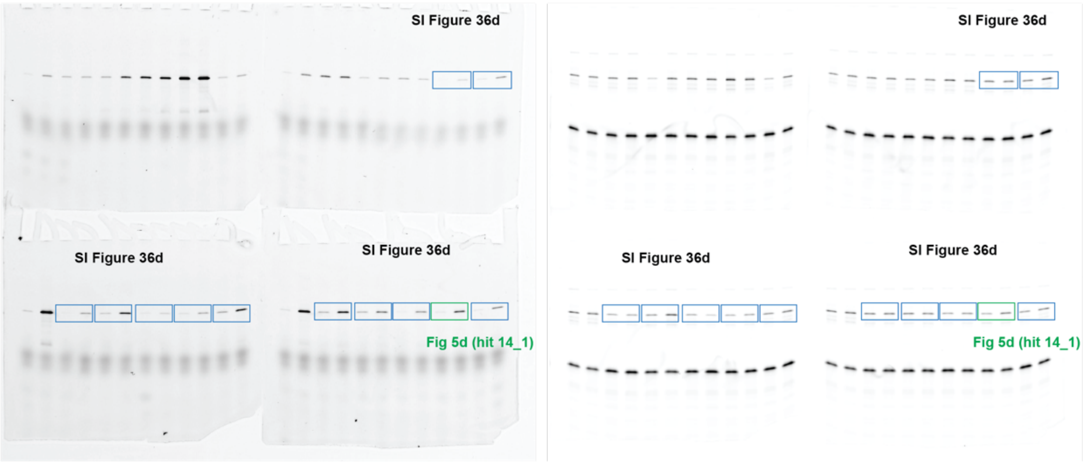

Cy5 Cy3

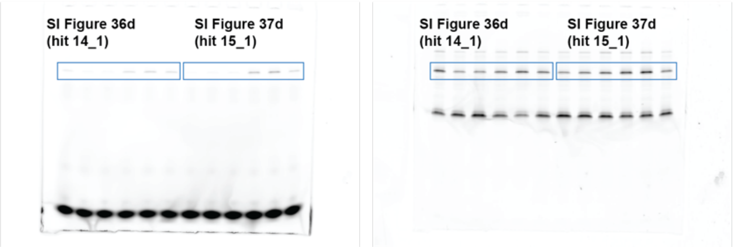

Cy5 Cy3

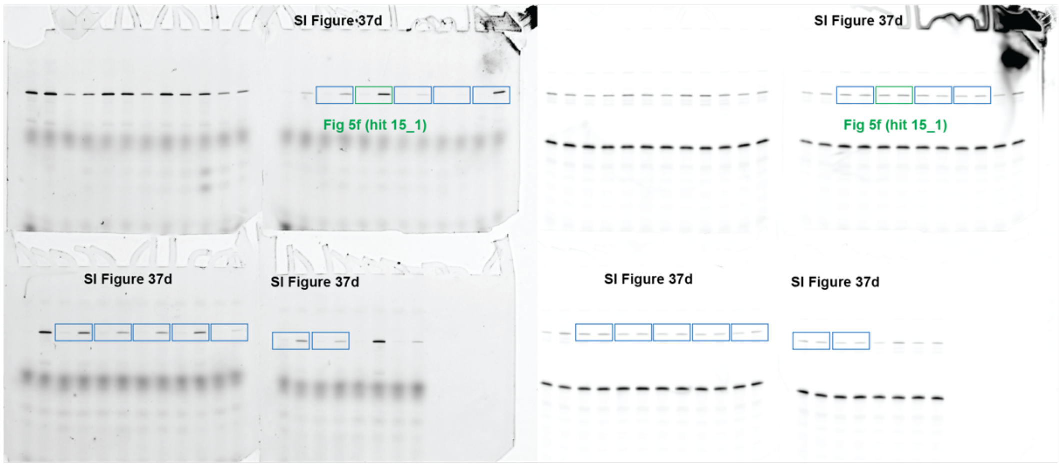

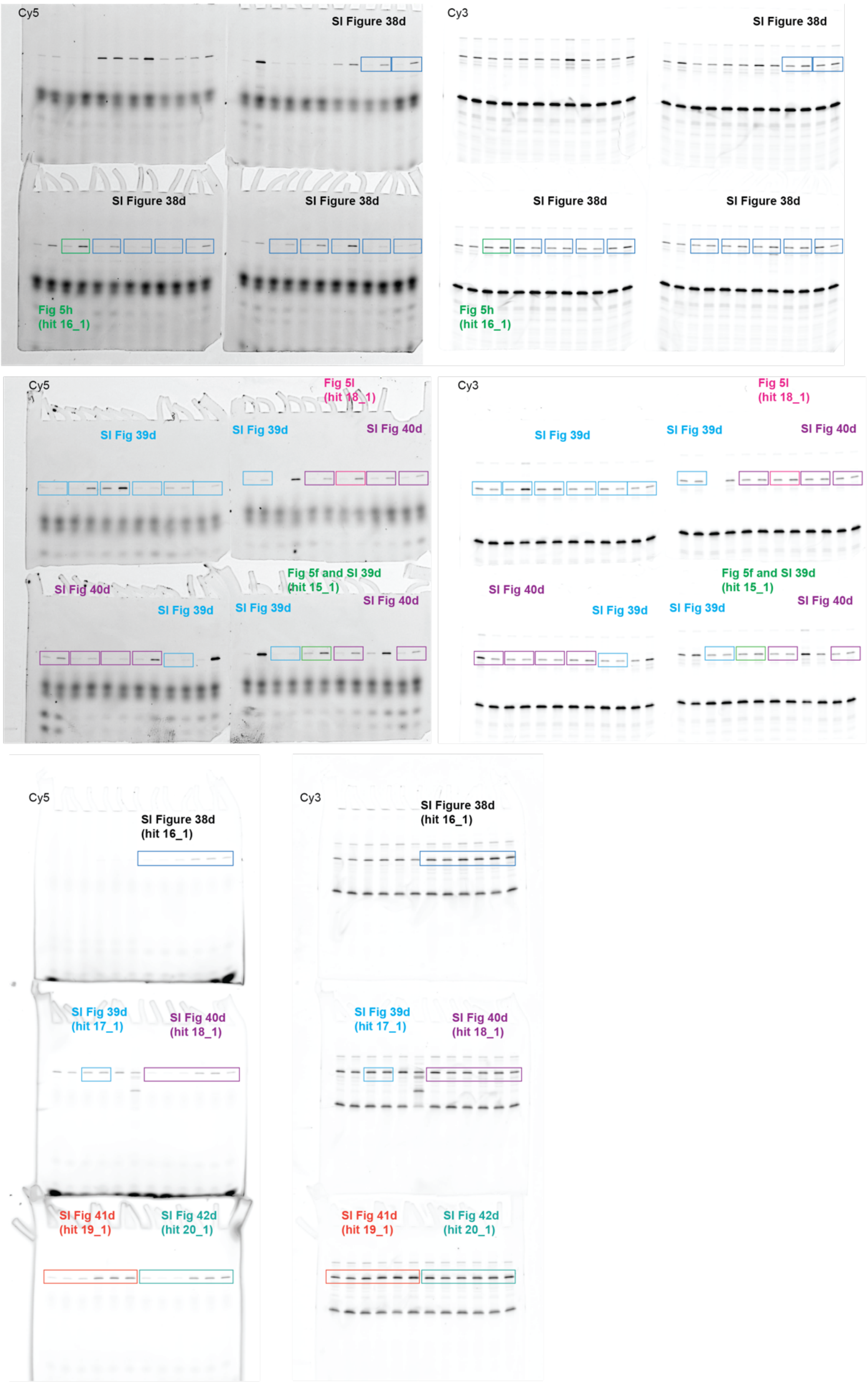

Cy5
SI Figure 38d
Cy3
SI Figure 38d
SI Figure 38d
SI Figure 38d
SI Figure 38d
SI Figure 38d
Fig 5h
(hit 16_1)
Fig 5h
(hit 16_1)
Cy5
Fig 5l
(hit 18_1)
SI Fig 39d
SI Fig 39d
SI Fig 40d
Cy3
Fig 5l
(hit 18_1)
SI Fig 39d
SI Fig 39d
SI Fig 40d
SI Fig 40d
Fig 5f and SI 39d
(hit 15_1)
SI Fig 39d
SI Fig 39d
SI Fig 40d
SI Fig 40d
Fig 5f and SI 39d
(hit 15_1)
SI Fig 39d
SI Fig 39d
SI Fig 40d
Cy5
SI Figure 38d
(hit 16_1)
Cy3
SI Figure 38d
(hit 16_1)
SI Fig 39d
(hit 17_1)
SI Fig 40d
(hit 18_1)
SI Fig 39d
(hit 17_1)
SI Fig 40d
(hit 18_1)
SI Fig 41d
(hit 19_1)
SI Fig 42d
(hit 20_1)
SI Fig 41d
(hit 19_1)
SI Fig 42d
(hit 20_1)

Cy5

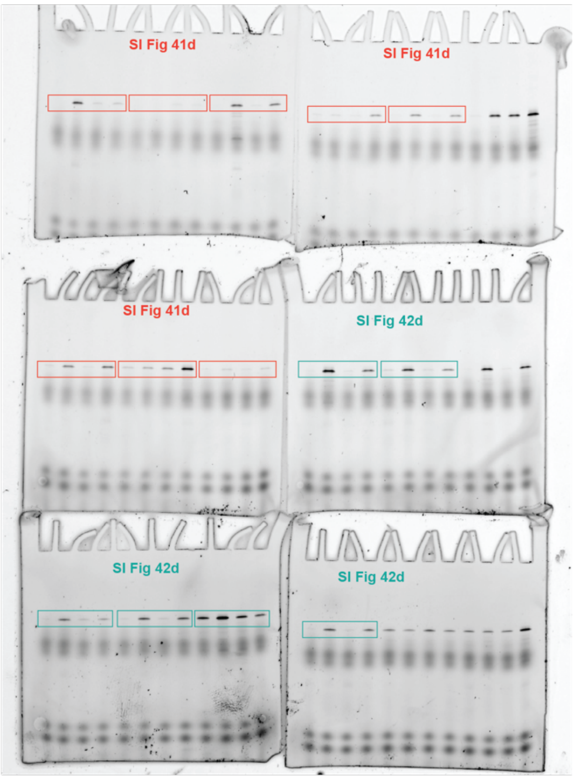
SI Fig 41d
SI Fig 41d
SI Fig 41d
SI Fig 42d
SI Fig 42d
SI Fig 42d

Cy3

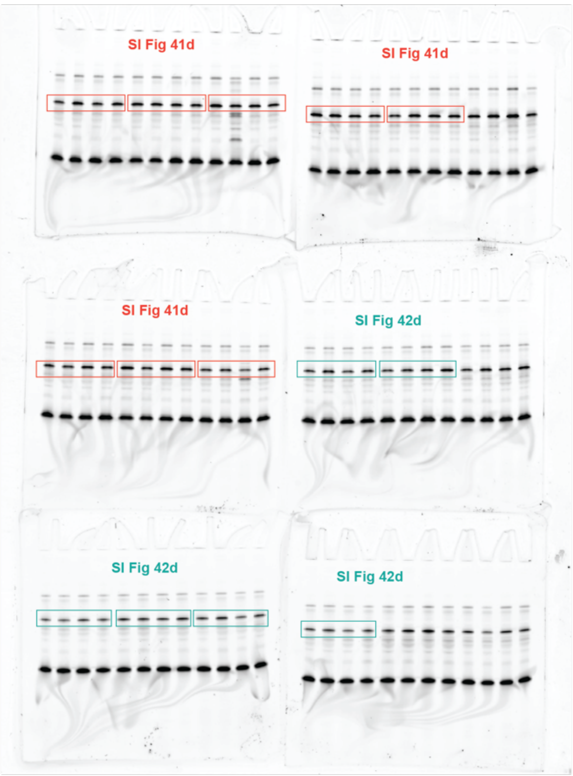
SI Fig 41d
SI Fig 41d
SI Fig 41d
SI Fig 42d
SI Fig 42d
SI Fig 42d

SI Figure 43a

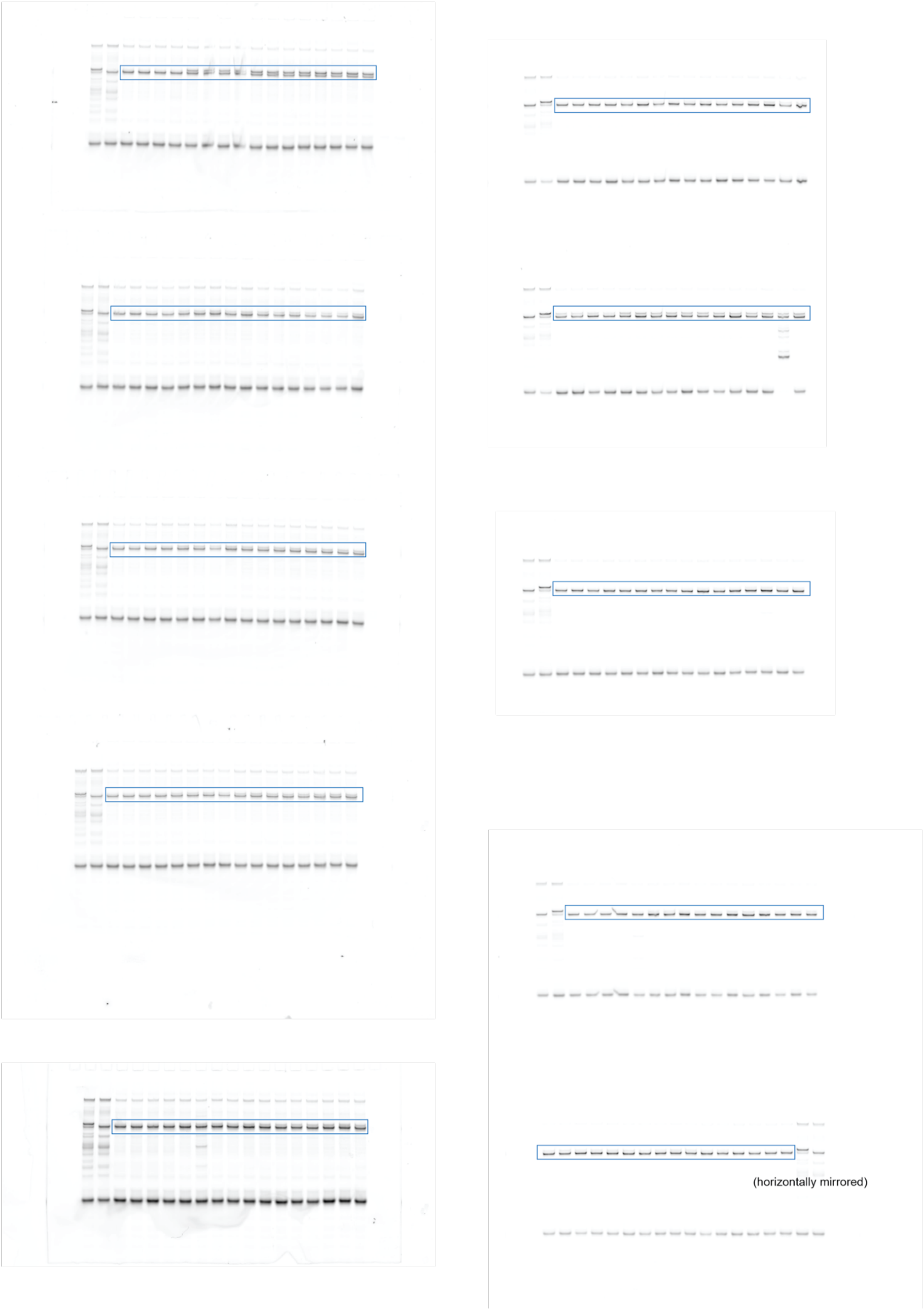

Supplementary Figure 1. Source data of all gels run in this work.

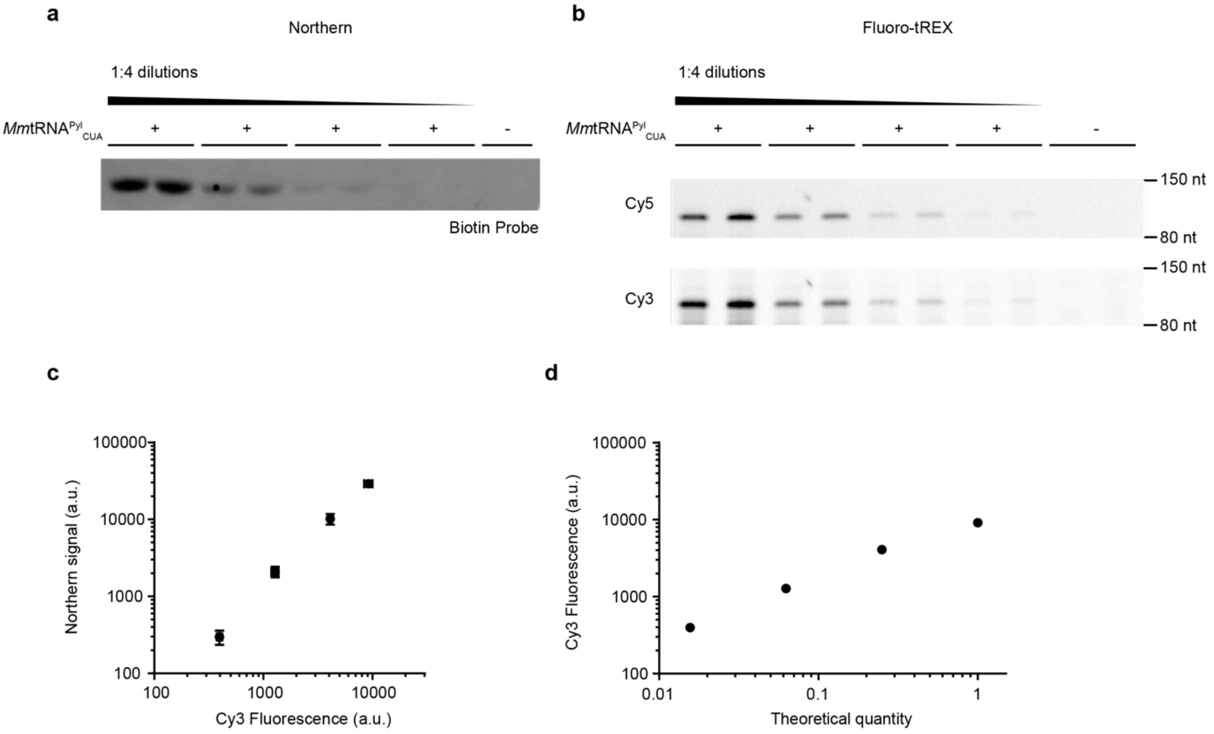

Supplementary Figure 2. The DNA probe used throughout this work is specific for *Mm*tRNA$^{Pyl}_{CUA}$ and, when fluorescently labeled, provides a loading control for the relative concentration of *Mm*tRNA$^{Pyl}_{CUA}$ in fluoro-tREX experiments.

a, Northern blot showing the relative amount of *Mm*tRNA$^{Pyl}_{CUA}$ used in the fluoro-tREX experiments shown in panel **b**. We isolated tRNAs from DH10β cells harboring a pMB1 plasmid encoding *Mm*tRNA$^{Pyl}_{CUA}$ or no cells harboring no plasmid. tRNA samples isolated from cells containing *Mm*tRNA$^{Pyl}_{CUA}$ were used undiluted and diluted into tRNAs isolated from cells without *Mm*tRNA$^{Pyl}_{CUA}$ at ratios of 1:4, 1:16, 1:64 and northern blots were run on the tRNA samples, using the previously verified[1] *Mm*tRNA$^{Pyl}_{CUA}$ specific probe 5'(Btn)-TGGCGGAAACCCCGGGAATCTAACCCGGCT-3'. The data shows a dilution series of *Mm*tRNA$^{Pyl}_{CUA}$ and confirms that the northern blot signal is dependent on the presence of *Mm*tRNA$^{Pyl}_{CUA}$ in the tRNA sample. We carried out the experiments in biological duplicates, with similar results. **b,** The Cy3 signal in fluoro-tREX decreases with dilutions of *Mm*tRNA$^{Pyl}_{CUA}$. Fluoro-tREX was run on the tRNA samples characterized in **a**. In brief, the fluorescently labeled DNA probe 5'GGGCCCATTAACATCACCTGGCGGAAACCCCGGGAATCTAACCCGGCT-3'Cy3 was annealed to *Mm*tRNA$^{Pyl}_{CUA}$ and the probe was extended by Klenow (exo-) in the presence of Cy5-dCTPs.

We ran a urea PAGE gel and visualized Cy3 as well as Cy5 fluorescence. **c**, correlation of Cy3 signal and fluorescence signal determined by northern blot. Band intensities were determined by densitometry using ImageJ. The two signals showed a strong and significant correlation (R^2 value=0.9935, p value=0.0032). **d**, correlation between the observed Cy3 signal and the theoretical quantity of *Mm*$tRNA^{Pyl}_{CUA}$; this is the relative amount of *Mm*$tRNA^{Pyl}_{CUA}$ predicted to be in each sample on the basis of the dilution series (1, 1:4, 1:16, 1:64) . Cy3 fluorescence robustly reflects the amount of loaded tRNA (R^2 value=0.9657, p value=0.0173). Experiments were carried out in two biological replicates producing similar results.

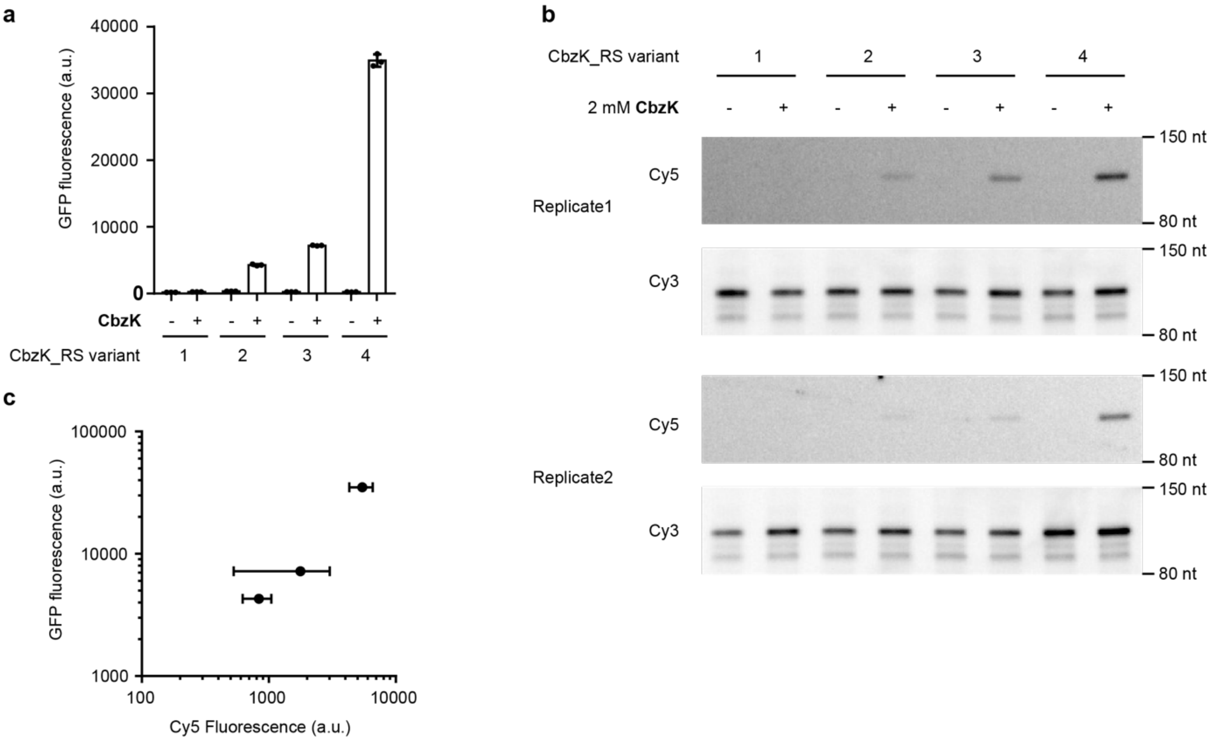

Supplementary Figure 3. Characterizing the acylation activity of PylRS variants which support amber suppression activity over two orders of magnitude, by fluoro-tREX.

a, Production of GFP150CbzK$_{His6}$ from *GFP150TAG$_{His6}$* from cells harboring a pMB1 plasmid encoding one of four different N^6-((*benzyloxy*)carbonyl)-*L*-lysine (CbzK) **2** variants of PylRS (RS1: Y306G, L305G; RS2: Y306G, N346G; RS3: Y306S; RS4: Y306G) and *Mm*tRNA$^{Pyl}_{CUA}$ and a p15A plasmid encoding *GFP150TAG$_{His6}$* in the presence and absence of 2 mM CbzK **2**. PylRS variants 1-4 lead to amber suppression activity over two orders of magnitude, when measured by fluorescence of GFP150CbzK$_{His6}$. Dots represent the mean of three biological replicates, error bars show ± s.d.. All numerical values are provided (**Supplementary Data 1**). **b,** The signal of fluoro-tREX can resolve the aminoacylation activity of all CbzK PylRS variants characterized in panel **a**. We isolated total tRNA from DH10β cells harboring a pMB1 plasmid encoding one of the four PylRS variants (RS1, RS2, RS3, or RS4) and *Mm*tRNA$^{Pyl}_{CUA}$ and performed fluoro-tREX. **c**, Cy5 signal vs GFP fluorescence plot for CbzK-RS 2, 3, and 4. The Cy5 fluorescent signal for these three variants corresponded well to the observed levels of GFP expression, permitting the identification and distinction of low as well as high

activity PylRS variants via fluoro-tREX. Experiments were carried out in two biological replicates producing similar results.

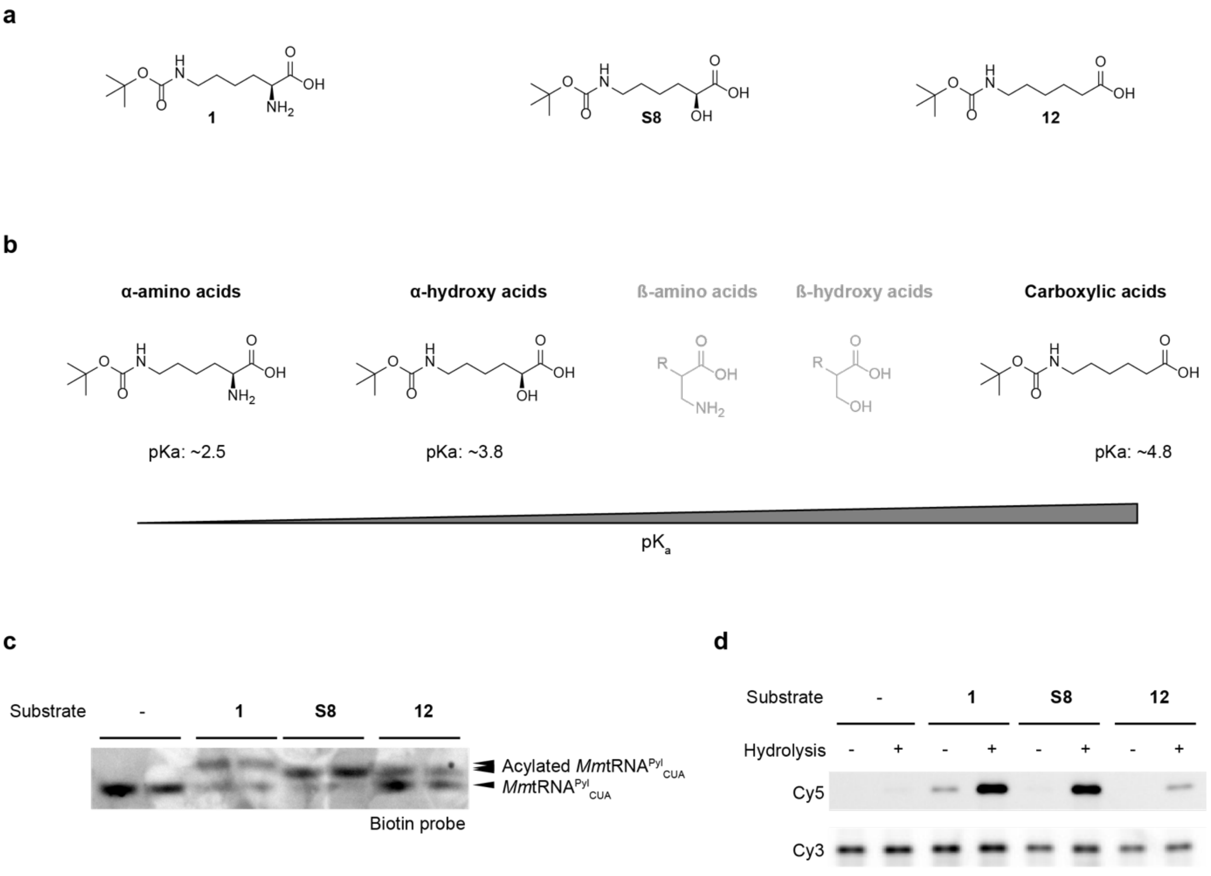

Supplementary Figure 4. Following oxidation, the deacylation of tRNAs under alkaline conditions increases the acylation signal in fluro-tREX and thereby permits the robust detection of acylation by hydroxy acids, and carboxylic acids.

a, Chemical structure of N^6-(*tert*-butoxycarbonyl)-*L*-lysine (BocK) **1**, (*S*)-*6*-((tert-butoxycarbonyl)amino)-2-hydroxyhexanoic acid (OH-BocK) **S8** as well as 6-((*tert*-butoxycarbonyl)amino)hexanoic acid (BocAhx) **12**. *Mm*PylRS is highly active with BocK **1**, OH-BocK[2] **21** and, and shows acylation activity with BocAhx **12**[3]. **b**, The free acid of **1**, **S8** and **12** span a range of the estimated pK_as. The rate constant for the alkaline hydrolysis of esters, to give a fixed alcohol and a variable carboxylic acid, increases as the pK_a of the resulting carboxylic acid decreases. We therefore expect the rate of hydrolysis for acylated tRNAs to be slower when the acylating monomers are α-hydroxy acids, simple carboxylic acids (and β-amino acids), than when the acylating monomers are α-amino acids. The pK_a values given are predicted with *MolGpka*.[4] **c**, *Mm*PylRS acylates *Mm*tRNA^{Pyl} in cells with BocK **1**, OH-BocK **S8**, and BocAhx **12** respectively. Northern blot of

*Mm*tRNA^{Pyl} from tRNAs isolated from cells harboring a pMB1 plasmid encoding the *Mm*PylRS/*Mm*tRNA^{Pyl} pair in presence and absence of BocK, OH-BocK, or BocAhx. The experiments were carried out in three biological replicates producing similar results. **d**, After oxidation, a deacylation step under alkaline conditions is necessary to robustly detect acylation activity of *Mm*PylRS by fluoro-tREX with non-alpha amino acid substrates. Fluoro-tREX was performed with the tRNA samples described in panel **c** with and without an incubation of the tRNAs for 45 minutes with 50 mM bicine at pH 9.6 post oxidation. The acylation signals from OH-BocK as well as BocAhx were dependent on the deacylation of the tRNAs before the Klenow (exo-) extension step of the protocol. The experiments were carried out in three biological replicates producing similar results.

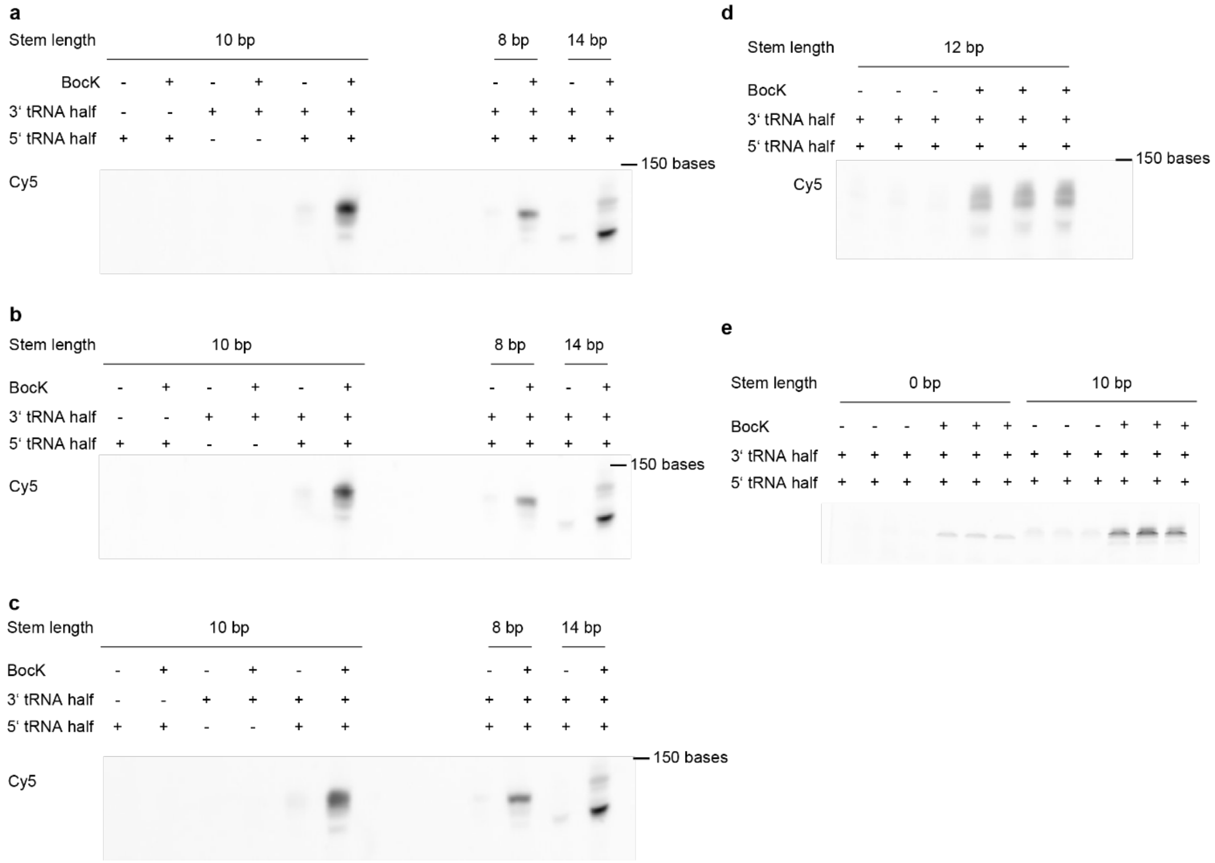

Supplementary Figure 5. Split *Mm*tRNA^{Pyl}s require a base-pairing stem region of ten bases to be efficiently acylated by *Mm*PylRS in cells when both tRNA halves are expressed *in trans*.

a-c, Acylation of split *Mm*tRNA^{Pyl} in cells was dependent on the presence of both tRNA halves and a base-pairing region of ten base-pairs. tRNAs were isolated from DH10β cells harboring split tRNA constructs with base-pairing stems of eight, ten or fourteen base-pairs where the 3'*Mm*tRNA^{Pyl} half was encoded on a pMB1 plasmid, and the 5' *Mm*tRNA^{Pyl} half on a p15A plasmid and the cells were grown in presence and absence of BocK. For the split tRNA construct with a ten base-pair long stem, cells were also grown with pMB1, or p15A plasmids lacking either the 3', or 5' tRNA half respectively. Fluoro-tREX was performed with isolated tRNAs. For stems of eight base-pairs length, a weak acylation signal was observed by fluoro-tREX, and for stems which were fourteen base-pair long, cleavage products were predominantly observed by fluoro-tREX. The experiments were carried out in three biological replicates producing similar results. **d**, The same as for **a-c** but with a split tRNA construct with a twelve base-pair long stem region, leading to a weaker acylation signal when compared to a ten base-pair long stem and to the observations of multiple bands, which are likely to result from

stem cleavage. For split tRNAs produced in *trans* we purified and concentrated the extension reaction before loading, following the general procedure for fluro-tREX B. Under these conditions we do not observe the Cy3 signal for the probe associated with the extension product. The experiments were carried out in three biological replicates producing similar results. **e**, Comparison of the ten base-pair long stem to the zero stem. Experiments were performed as described for **a-c.** The experiments were carried out in three biological replicates producing similar results.

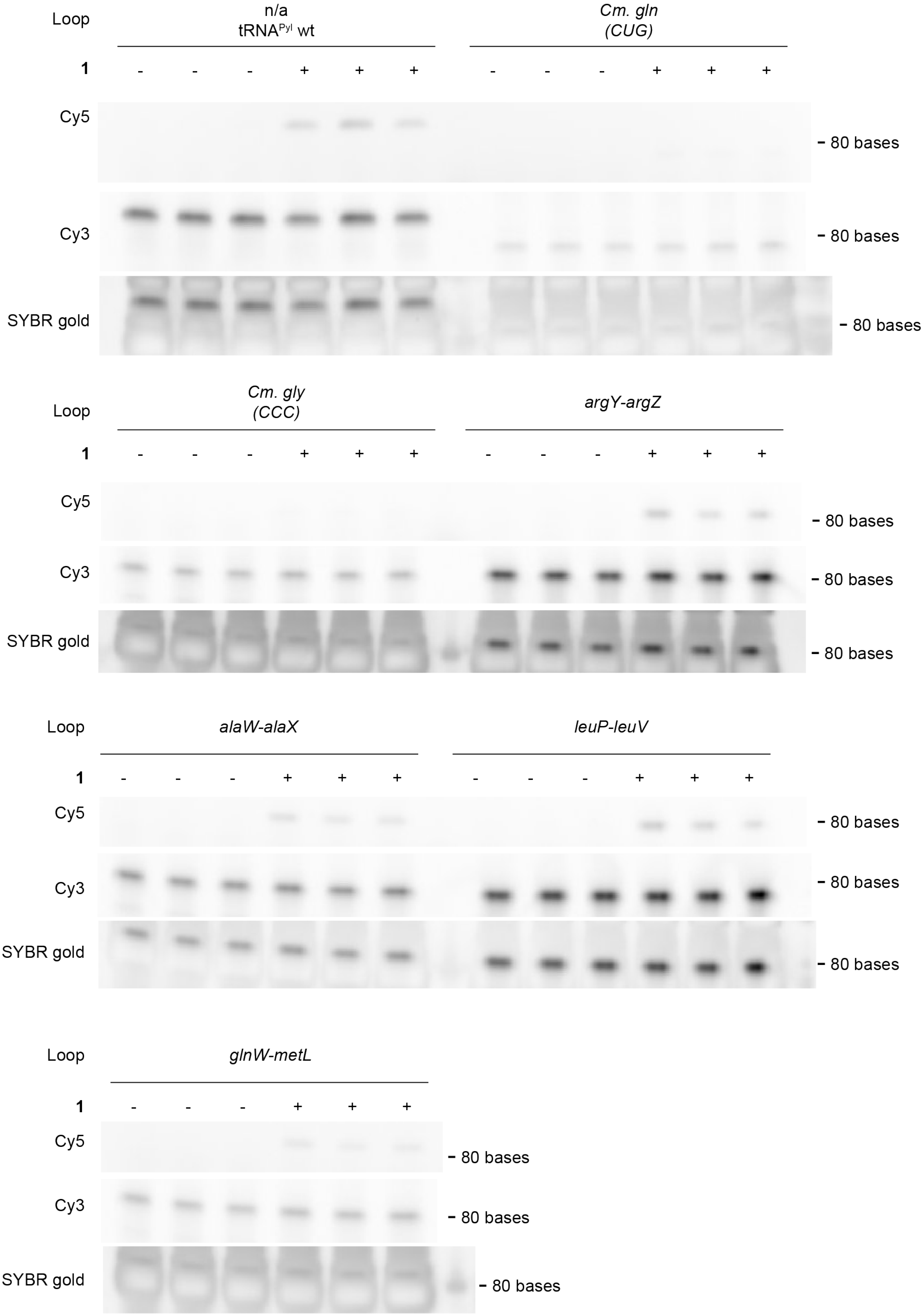
Loop
n/a
tRNAPyl wt
Cm. gln
(CUG)
1
- - - + + +
- - - + + +
Cy5
Cy3
SYBR gold
- 80 bases
- 80 bases
- 80 bases
Loop
Cm. gly
(CCC)
argY-argZ
1
- - - + + +
- - - + + +
Cy5
Cy3
SYBR gold
- 80 bases
- 80 bases
- 80 bases
Loop
alaW-alaX
leuP-leuV
1
- - - + + +
- - - + + +
Cy5
Cy3
SYBR gold
- 80 bases
- 80 bases
- 80 bases
Loop
glnW-metL
1
- - - + + +
Cy5
Cy3
SYBR gold
- 80 bases
- 80 bases
- 80 bases

Supplementary Figure 6. The sequence of the loop region of circularly permutated split tRNAs is crucial for the robust expression and acylation of split tRNAs in cells.

Split tRNAs were isolated from DH10β cell harboring a pMB1 plasmid encoding one of seven circularly permutated *Mm*$tRNA^{Pyl}$ constructs, each with a different loop region (*Cm. gln*, *Cm. gly*, *E coli* (*Ec.) argY-argZ*, *Ec. alaW-alaX*, *Ec. leuP-leuV*, *Ec. glnW-metL*) in presence and absence of 4 mM BocK **1**. Intact *Mm*$tRNA^{Pyl}_{CUA}$ was produced as a control. Split tRNAs were isolated and fluoro-tREX was performed. Split *Mm*$tRNA^{Pyl}$ constructs with loops constituting of the *E. coli* intergenic regions *argY-argZ*, or *leuP-leuV* led to high acylation and split tRNA expression levels as judged by Cy5, or Cy3 fluorescence respectively, which were comparable to the signals observed for intact *Mm*$tRNA^{Pyl}_{CUA}$. Mean Cy3 signal for *Mm*$tRNA^{Pyl}$ 10185 ± 1102; for *argZ-argY* 12063 ± 630; for *leuP-leuV* 13467 ± 465. Mean Cy5 signal for *Mm*$tRNA^{Pyl}$ 1656 ± 62; for *argZ-argY* 1397 ± 66; for *leuP-leuV* 1442 ± 67 Cy3 and Cy5 signals were determined by densionmetry. The experiments were carried out in three biological replicates producing similar results.

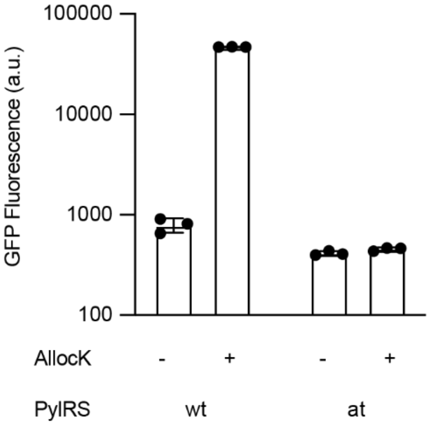

Supplementary Figure 7. Protein production, via read through of an amber stop codon, by wild type (wt) and attenuated (at) PylRS/tRNA$^{Pyl}_{CUA}$ pairs.

Production of GFPAllocK$_{His6}$ from *GFP150TAG*$_{His6}$ from cells harboring a pMB1 plasmid encoding either wild type (wt) *Mm*PylRS or an attenuated (at) mutant (H338A, F342A, M344A, E396A, S399A) and *Mm*tRNA$^{Pyl}_{CUA}$ and a p15A plasmid encoding *GFP150TAG*$_{His6}$ in the presence and absence of 2 mM *N*6-((allyloxy)carbonyl)-L-lysine (AllocK). Dots represent the mean of three biological replicates, error bars show ± s.d.. All numerical values are provided (**Supplementary Data 1**).

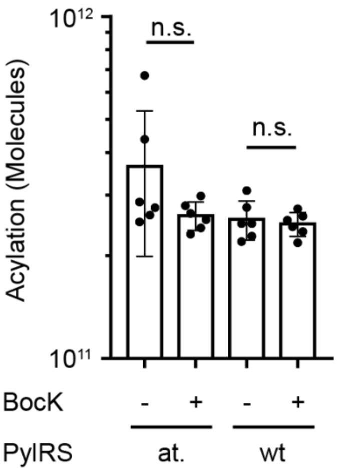

Supplementary Figure 8. Input samples for bio-mREX of wt and attenuated stmRNA.

stmRNAs were extracted and oxidized, using method A (**Methods**). A fraction of resulting stmRNA was reverse transcribed, and then quantified via qPCR in the same manner as the samples subjected to extension with biotinylated nucleotides and pull down. Addition of BocK did not lead to a significant difference in isolated stmRNA levels (attenuated PylRS: p value= 0.1648, wt PylRS: p value= 0.6390).

a

	Sequence	Predicted translational rate
RBS_1	TTGTCCACCCACTCAAGGC	1
RBS_2	CTCTGTCTGCCCCCCTACCGAAG	5
RBS_3	GTCAGAGTATAACAATCATACCCCGAG	10

b

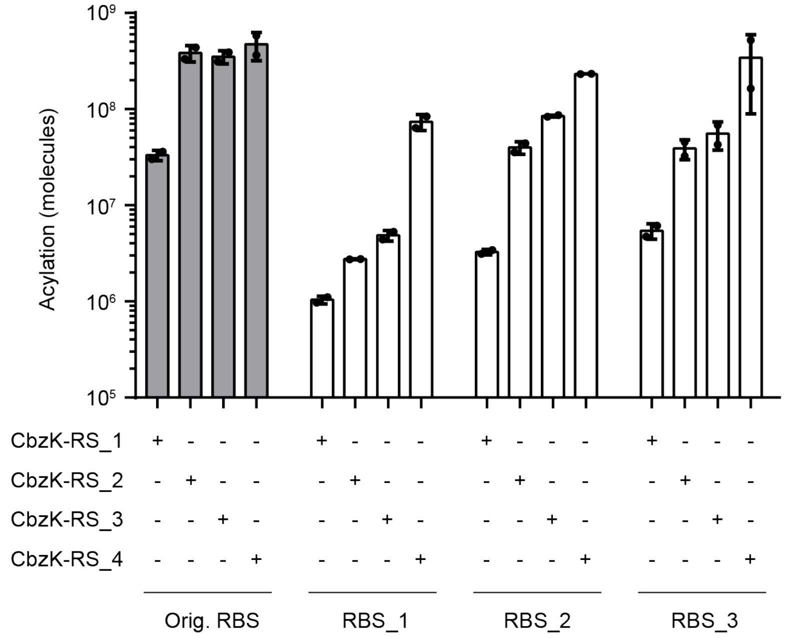

Supplementary Figure 9. Resolving the activity of PylRS variants in bio-mREX via 5'UTR tuning. **a**, Nucleotide sequences of 5'UTR sequences RBS1-3 with translation initiation rates given. Sequences were generated and initiation rates predicted by *DeNovo DNA*. **b**, Cells harboring a plasmid encoding stmRNAs bearing a PylRS CbzK mutant (see **Supplementary Figure 3**) under the control of either the initial 5'UTR, or one of three designed 5'UTR sequences were grown in presence of 2 mM CbzK **2**, stmRNA transcription was induced for 20 minutes, total RNA isolated by phenol chloroform extraction, and bio-mREX was performed. For the stmRNA construct with the initial 5'UTR region (stmRNA^{vol1}) low activity PylRS mutants (e.g. CbzK-RS2) led to a saturation of the acylation signal. For all designed 5'UTR sequences, which were designed to lead to low translation levels, low activity PylRS variants could be resolved by bio-mREX. The stmRNA under the control of RBS2 (stmRNA^{vol2}) led to a good correlation of PylRS activity as measured by amber suppression of *GFP150TAG$_{His6}$* and the acylation data measured by bio-mREX (see **Fig. 3e**) and was used for all future experiments. Dots represent the

mean of three biological replicates, error bars show ± s.d.. All numerical values are provided (**Supplementary Data 1**).

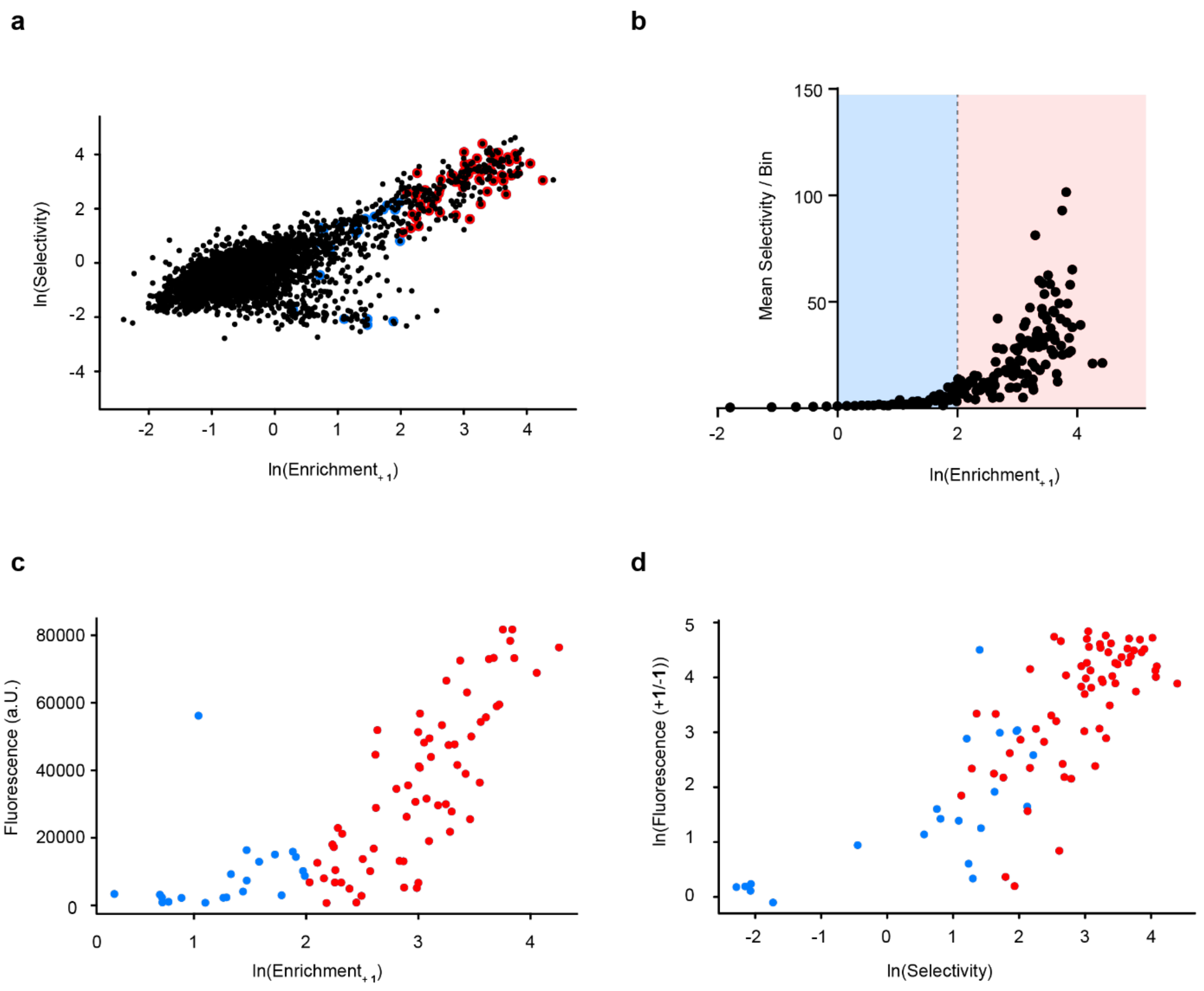

Supplementary Figure 10. tRNA display identifies active and selective orthogonal aaRS variants from an stmRNA library.

a, Spindle plot from the tRNA display selection using stmRNA library 1 and ncAA **1. b**, Identifying the region of the spindle plot enriched in active and selective clones. We expect the top right quadrant of the spindle plot to be enriched in active and selective clones. Since selectivity is derived from the ratio of sequence counts + ncAA and -ncAA, enriched clones with negative selectivity values would correspond to specific enrichment of a clone in the -ncAA condition with respect to the +ncAA condition. We postulated that most apparent enrichments of this type were spurious, and therefore that regions of the spindle plot where positive selectivity values were mirrored by negative selectivity values of the same magnitude may contain substantial noise. Based on this postulate we expected the active and ncAA selective clones to be most enriched in the region of the plot where, for a given positive

enrichment value, the selectivity becomes asymmetric. To enrich for this asymmetric population we binned mean selectivity values: 5350 points in the spindle plot (**Fig. 4c**) were divided into 500 equal bins along the enrichment$_{+1}$ dimension (163 bins contained data). The mean selectivity of each bin was plotted against the natural logarithm of enrichment$_{+1}$. From this, a threshold of ca 7.4 (corresponding to a logarithmic score value of 2) was used to define the enrichment value at which the spindle plot is asymmetric along the selectivity axis. **c**, Experimental GFP fluorescence values for 100 clones plotted against the natural logarithm of enrichment score in the presence of **1**. GFP was expressed from *GFP150TAG$_{His6}$* in the presence of the *Mm*PylRS variant clone, the cognate *Mm*tRNA$^{Pyl}_{CUA}$ and the ncAA (**1**). Points above the symmetry threshold are colored in red (65 points), points below are colored in blue (21 points). There is a strong positive and significant correlation between the tRNA display sequence data and experimental expression data for the red points (R-square value=0.6611, $p<0.0001$ value), but no significant correlation for the blue points (R^2 value=0.0392, p value=0.397), this is consistent with our postulate. The red points are also shown in **Fig. 4c**. In subsequent selections, on the basis of this analysis, we primarily focused on identifying clones on the right-hand side of the spindle plot where, for a given enrichment value, the magnitude of the positive selectivity value for a clone is of greater magnitude than negative selectivity values for clones with the same enrichment value. **d**, tRNA display identifies ncAA specific PylRS variants. Plot shows the experimental selectivity vs selectivity from the spindle plot for the clones show in panel **b**, color coding as in panel **b**. The experimental selectivity is derived from GFP expression experiments, as in panel **b**, but +/- ncAA.

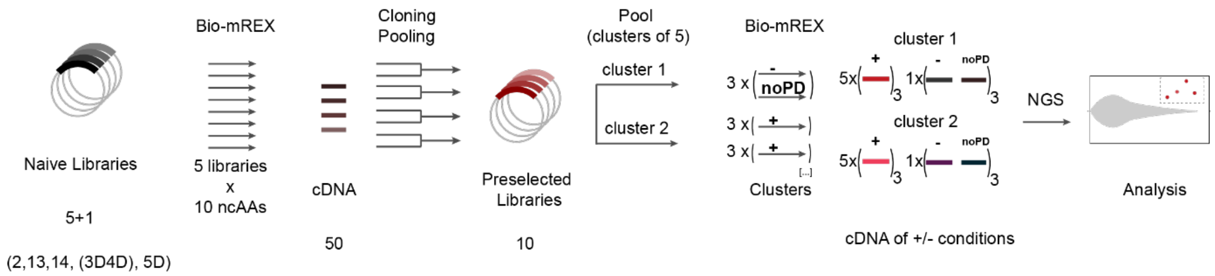

Supplementary Figure 11. Schematic representation of tRNA display based strategy for selecting PylRS variants that direct the incorporation of ncAAs into proteins.

In the first round naïve stmRNA^{vol2} libraries 2, 13, 14, 3D, 4D, and 5D were transformed into BL21 cells and grown overnight. Libraries 3D and 4D were combined to generate library 3D4D. The five libraries were grown to OD_{600} of 0.3-0.4 and 2.6 mL of the cell culture from each library was added to a stock solution of each ncAA; this resulted in 50 samples (five libraries x ten ncAAs). Cells were grown for 40 min, stmRNAs induced, and cells grown for another 20 min. Bio-mREX was performed on the isolated RNA for each of the 50 samples. For each reaction, cDNA was amplified with primers suitable for Golden Gate assembly. Then all amplicons of the libraries selected for the same ncAA were combined at equimolar ratios (resulting in ten combined libraries in total) and cloned into a fresh ColE1vector backbone. This created ten pre-selected libraries. The ten pre-selected libraries were transformed into BL21 cells and grown over night. The preselected libraries for ncAAs **2** - **6** were combined to create a single cluster library. Similarly, the preselected libraries for ncAAs **7** – **11** were combined to create a second cluster library. 2.6 mL of the first cluster library was added to solutions of ncAAs **2** – **6.** 2.6 mL of the first cluster library was also added to a sample without ncAA, as a control. Cells were grown for 40 min, stmRNAs induced and cells grown for another 20 min, and the RNA isolated. Three RNA samples were converted to cDNA as bio-mREX input control. Bio-mREX was performed on the isolated RNA. This generated seven samples (bio-mREX input, -ncAA control for bio-mREX, and five bio-mREX samples for ncAAs **2** – **6**. The experiment was performed in triplicates, generating 21 samples. The cDNA of each sample was sequenced by NGS and analyzed to generate spindle plots and sequence tables. The second cluster was treated analogously to the first cluster, unsing ncAAs **7** – **11** in place of **2** – **6**.

a

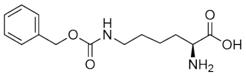

2

b

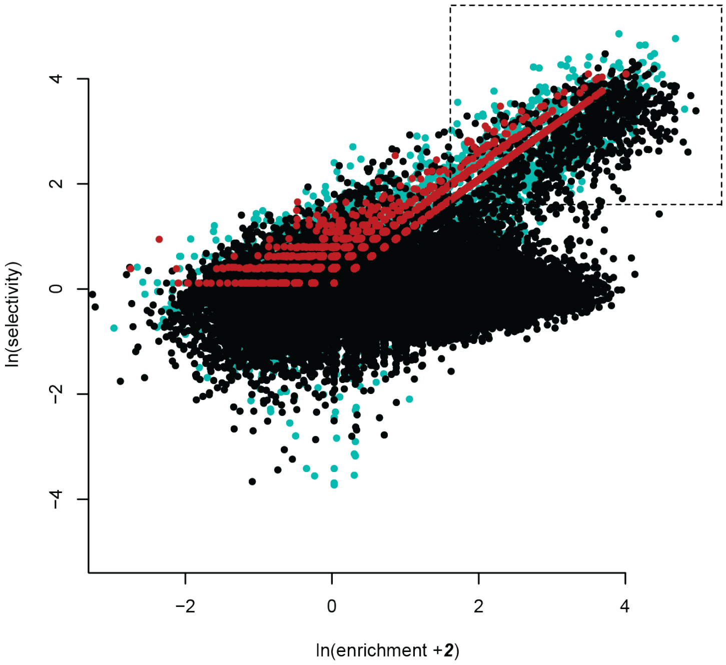

c

M300	A302	L305	Y306	L309	M344	N346	C348	Y384	S399	V401	selectivity	enrichment
M	A	L	A	G	Q	N	F	Y	S	V	128.5	50.0
M	A	L	A	L	Q	Q	N	Y	T	V	117.3	108.0
M	A	L	Y	V	M	N	G	Y	S	K	103.8	71.5
V	D	L	R	C	M	N	C	Y	S	V	103.1	66.4
M	A	L	A	A	Q	H	V	Y	S	V	88.1	81.1
M	S	L	G	L	M	N	C	Y	S	V	87.8	41.4
M	A	V	A	L	M	N	V	F	T	V	83.6	77.0
M	A	L	Y	S	Q	N	G	Y	T	V	82.7	33.3
D	E	V	R	V	M	N	C	Y	S	V	79.4	25.0
F	S	L	A	L	M	N	C	Y	S	V	78.7	40.5
M	A	L	F	L	Q	N	G	Y	T	V	75.5	60.1
M	A	L	I	S	M	N	A	Y	S	V	74.3	69.4
M	A	L	A	L	Q	H	L	Y	S	V	74.1	68.2
L	A	L	M	S	M	N	C	Y	S	V	73.7	50.0
M	A	L	Y	V	Q	N	G	Y	T	V	73.6	49.9
M	A	L	M	G	M	N	A	Y	S	V	71.4	65.7
M	A	L	A	L	Q	Q	N	Y	S	K	71.2	22.7
M	A	V	A	L	Q	Q	N	Y	S	V	70.9	65.3
M	A	V	Y	V	M	N	G	F	T	V	70.9	32.6
M	A	L	Y	S	Q	N	N	Y	S	V	70.6	65.2
M	A	L	V	L	Q	E	N	Y	T	V	70.6	81.4
F	A	L	A	S	M	N	C	Y	S	V	70.6	65.0
M	A	L	A	A	M	H	L	Y	S	V	69.8	49.2
M	A	L	A	G	Q	N	A	Y	S	V	69.5	24.4
M	A	V	A	L	Q	Q	N	Y	S	C	68.9	84.1

Supplementary Figure 12. Selection of PylRS variants for CbzK 2 by tRNA display.

a, Chemical structure of CbzK **2**. **b,** Spindle plot resulting from the tRNA display selection as described in **Supplementary Figure 11**. The selectivity is defined as the ratio of the relative abundance of a particular sequence in the positive samples (+ **2**), divided by the relative abundance in the control sample (-ncAA). The enrichment is defined as the ratio of the same sequence in the positive samples, divided by the relative abundance in the input library. The boxed region indicates selectivities of ≥ 5, and enrichments of ≥ 5. Black dots are sequences observed in all positive samples and all control samples.

Green dots are sequences observed in all positive samples, and minimally one control sample. Red dots are sequences only observed in the positive samples. **c**, Top 25 sequences ordered by selectivity resulting from the selection for substrate **2** with selectivities of ≥ 5, and enrichments of ≥ 5.

a

3

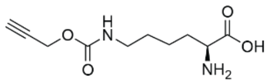

b

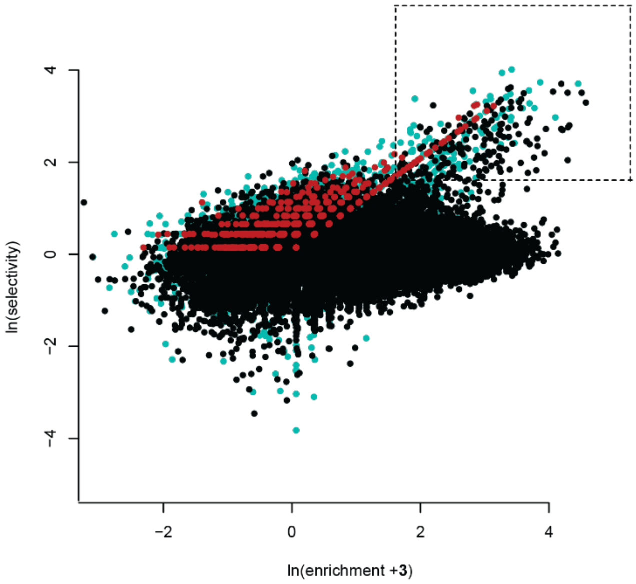

c

hit name	M300	A302	L305	Y306	L309	M344	N346	C348	Y384	S399	V401	selectivity	enrichment
3_1	M	A	L	Y	L	Q	Q	V	Y	T	V	55.3	30.6
3_2	M	A	L	Y	L	Q	H	V	Y	S	S	51.5	26.3
3_3	M	A	L	Y	L	Q	H	L	Y	S	V	41.9	47.7
	M	A	L	Y	F	M	Q	T	Y	T	V	40.6	86.1
	M	A	L	Y	L	Q	H	V	Y	S	V	40.5	66.2
	M	A	L	Y	F	Q	Q	S	Y	T	V	37.5	30.1
	M	A	L	F	L	Q	N	G	Y	T	V	36.4	28.9
	M	A	L	F	L	Q	N	S	Y	T	V	35.1	40.1
	M	A	L	Y	L	M	H	I	Y	S	V	34.8	16.7
	M	A	L	Y	L	Q	Q	T	Y	T	V	34.2	58.2
	M	A	L	Y	L	Q	N	V	Y	T	V	33.4	73.1
	M	A	L	Y	L	Q	N	T	Y	T	V	33.4	90.9
	M	A	L	Y	L	Q	N	A	Y	T	V	33.0	36.9
	M	A	L	Y	L	Q	Q	T	Y	T	A	32.5	29.9
	M	A	L	Y	L	Q	Q	T	Y	S	A	31.1	21.9
	M	A	L	Y	F	Q	N	C	Y	T	V	30.5	28.1
	M	A	L	L	L	Q	N	S	Y	T	V	30.3	27.8
	M	A	L	L	L	Q	N	S	Y	S	V	29.3	6.8
	M	A	L	Y	L	Q	Q	N	Y	S	A	29.2	22.1
	M	A	L	Y	L	Q	Q	T	Y	S	C	29.1	35.5
	M	A	L	Y	A	Q	Q	T	Y	T	V	28.7	25.8
	M	A	L	Y	L	Q	H	A	Y	S	V	27.9	34.1
	M	A	L	Y	L	Q	Q	N	Y	T	C	27.5	20.5
	M	A	L	Y	C	Q	N	T	Y	T	V	27.3	30.2
	M	A	L	Y	F	Q	N	A	Y	T	V	27.2	34.4

d

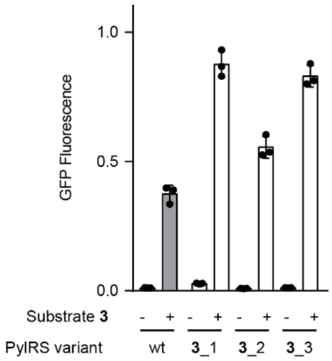

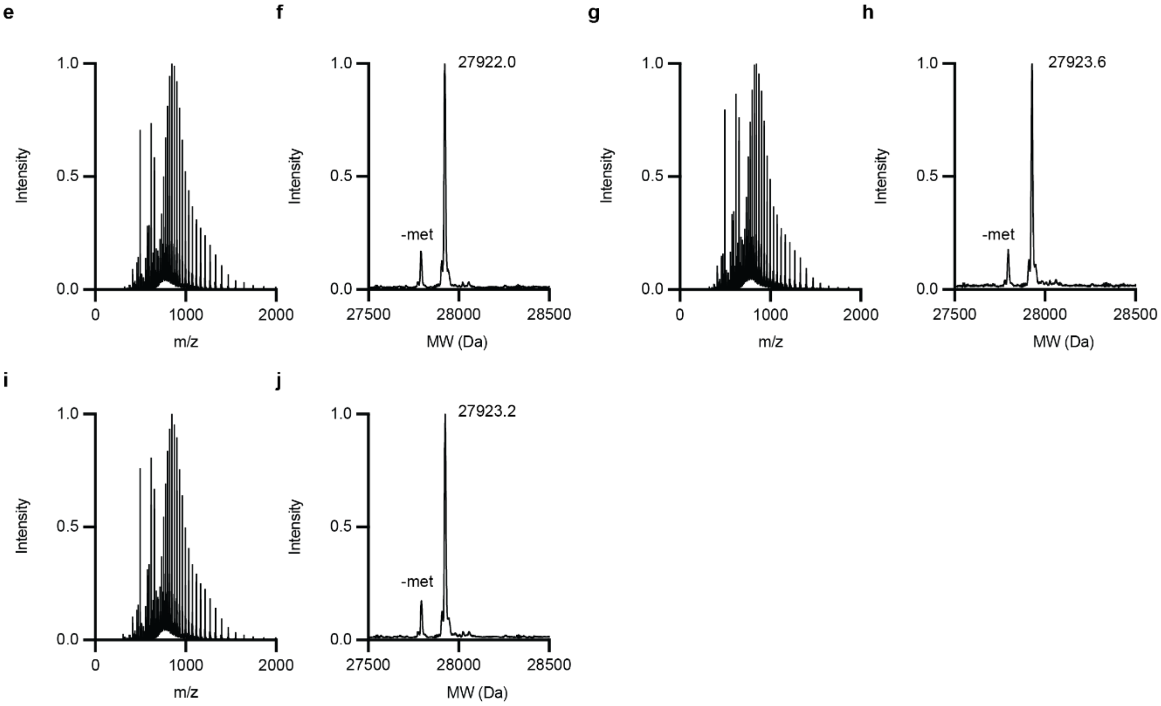

Supplementary Figure 13. Selection of PylRS variants for *N*[6]-((prop-2-yn-1-yloxy)carbonyl)-*L*-lysine (AlkyneK) 3 by tRNA display.

a, Chemical structure of AlkyneK **3**. **b,** Spindle plot resulting from the tRNA display selection as described in **Supplementary Figure 11**. The selectivity is defined as the ratio of the relative abundance of a particular sequence in the positive samples (+ **3**), divided by the relative abundance in the control sample (-ncAA). The enrichment is defined as the ratio of the same sequence in the positive samples, divided by the relative abundance in the input library. The boxed region indicates sequences with selectivities of ≥ 5, and enrichments of ≥ 5. Black dots are sequences observed in all positive samples and all control samples. Green dots are sequences observed in all positive samples, and at least one control sample. Red dots are sequences only observed in the positive samples. **c**, Top 25 sequences, ordered by selectivity, resulting from the selection for substrate **3** with selectivities of ≥ 5, and enrichments of ≥ 5. **d**, Amber suppressor activity data for selected PylRS variants measured by the production of GFP150AlkyneK$_{\mathrm{His6}}$ from *GFP150TAG*$_{His6}$ from cells harboring a pMB1 plasmid encoding either wild type (wt) *Mm*PylRS or the indicated *Mm*PylRS variants, and a p15A plasmid encoding *GFP150TAG*$_{His6}$ in the presence and absence of 4 mM **3**. The fluorescence is shown relative to cells harboring wt *Mm*PylRS, *Mm*tRNA$^{\mathrm{Pyl}}_{\mathrm{CUA}}$ expressing GFP from *GFP150TAG*$_{His6}$ in the presence of 2 mM substrate **1**. **e,** Raw mass spectrum for purified GFP150AlkyneK$_{\mathrm{His6}}$ produced with PylRS **3**_1. **f,** Deconvoluted mass spectrum resulting from the primary spectrum shown in panel **e**. Mass predicted 27923.3 Da, mass found 27922.0 Da. The minor peak labeled -met corresponds to cleavage of the N-terminal methionine residue. **g-h**, same as **e** and **f** but for GFP150AlkyneK$_{\mathrm{His6}}$ produced with PylRS **3**_2. Mass predicted 27923.3 Da, mass found 27923.6 Da. **i-j**, same as **e** and **f** but for GFP150AlkyneK$_{\mathrm{His6}}$ produced with PylRS **3**_3. Mass predicted 27923.3 Da, mass found 27923.2 Da.

a

4

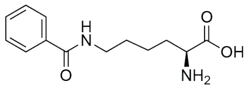

b

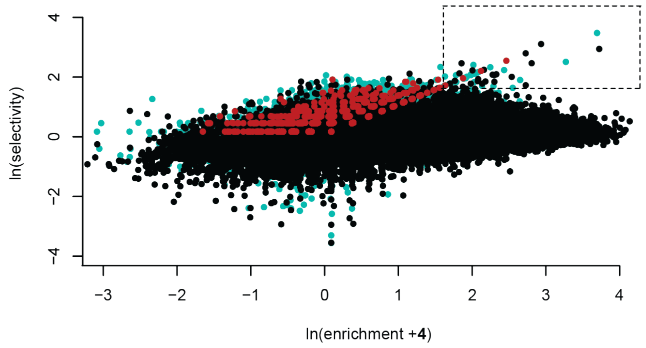

c

hit name	M300	A302	L305	Y306	L309	M344	N346	C348	Y384	S399	V401	selectivity	enrichment
4_1	M	A	F	Y	L	Q	Q	T	Y	T	V	32.3	40.2
4_2	M	A	L	Y	L	Q	N	V	Y	S	A	22.4	18.8
4_3	M	A	L	Y	L	Q	N	V	Y	T	V	18.9	41.4
4_4	M	A	L	Y	L	M	N	C	W	S	A	16.4	15.3
	M	A	F	Y	L	Q	Q	S	Y	T	V	12.8	11.7
	M	A	L	Y	L	M	N	C	W	S	G	12.3	26.3
	M	A	L	Y	L	Q	N	V	Y	S	C	11.7	16.6
	M	A	L	Y	M	M	N	T	F	S	A	11.7	6.4
	M	A	V	Y	L	Q	Q	T	Y	T	V	11.1	7.5
	C	H	F	Y	L	Q	V	V	Y	S	V	10.3	9.5
	D	E	L	R	L	M	D	C	Y	S	V	9.4	8.7
	M	A	L	Y	L	M	N	C	W	S	K	9.3	11.4
	M	A	L	Y	F	Q	Q	S	Y	T	V	9.2	7.4
	M	A	L	Y	L	Q	N	V	Y	T	C	9.1	8.3
	M	A	L	Y	A	M	A	L	F	S	V	8.8	8.1
	M	A	L	Y	M	M	N	V	F	S	A	8.6	7.9
	M	A	L	Y	L	Q	N	V	Y	S	K	8.4	6.2
	M	A	L	Y	L	M	N	V	F	S	A	8.3	7.7
	M	A	C	Y	L	M	T	M	F	S	C	8.3	7.7
	D	S	L	Y	L	M	G	F	Y	S	V	8.3	7.6
	M	A	L	Y	F	Q	N	A	Y	T	V	8.1	10.3
	M	A	F	Y	L	M	Q	T	Y	S	V	8.0	7.3
	M	A	I	Y	M	Q	V	W	F	T	V	8.0	9.8
	M	A	L	Y	L	Q	N	V	X	S	A	7.9	7.3
	M	A	L	Y	L	Q	N	V	Y	S	V	7.9	10.0

d

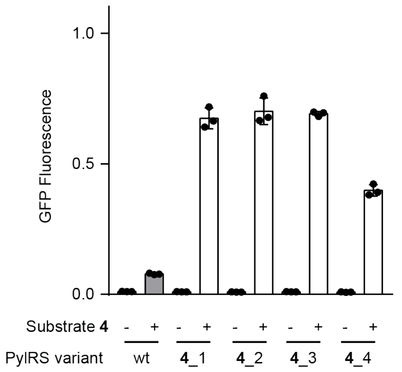

e **f** **g** **h** **i** **j** **k** **l**

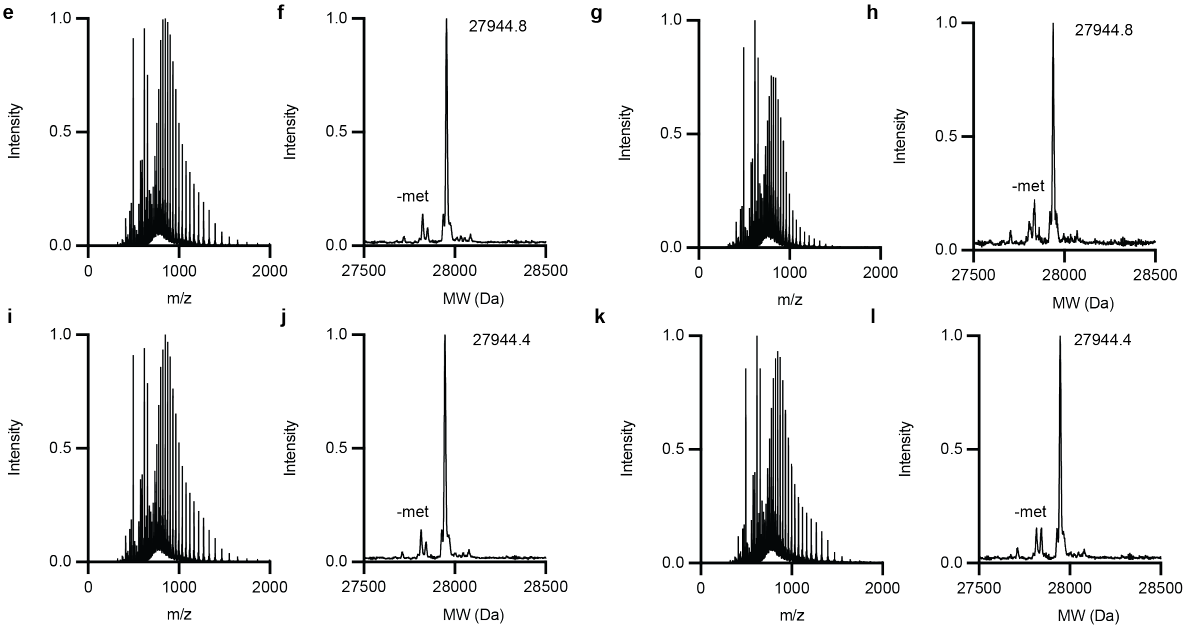

Supplementary Figure 14. Selection of PylRS variants for *N*⁶-benzoyl-*L*-lysine (BenzK) 4 by tRNA display.

a, Chemical structure of BenzK **4**. **b,** Spindle plot resulting from the tRNA display selection as described in **Supplementary Figure 11**. The selectivity is defined as the ratio of the relative abundance of a particular sequence in the positive samples (+ **4**), divided by the relative abundance in the control sample (-ncAA). The enrichment is defined as the ratio of the same sequence in the positive samples, divided by the relative abundance in the input library. The boxed region indicates sequences with selectivities of ≥ 5, and enrichments of ≥ 5. Black dots are sequences observed in all positive samples and all control samples. Green dots are sequences observed in all positive samples, and at least one control sample. Red dots are sequences only observed in the positive samples. **c**, Top 25 sequences, ordered by selectivity, resulting from the selection for substrate **4** with selectivities of ≥ 5, and enrichments of ≥ 5. **d**, Amber suppressor activity data for selected PylRS variants measured by the production of $GFP150BenzK_{His6}$ from $GFP150TAG_{His6}$ from cells harboring a pMB1 plasmid encoding either wild type (wt) *Mm*PylRS or the indicated *Mm*PylRS variants, and a p15A plasmid encoding $GFP150TAG_{His6}$ in the presence and absence of 4 mM **4**. The fluorescence is shown relative to cells harboring wt *Mm*PylRS, *Mm*$tRNA^{Pyl}_{CUA}$ expressing GFP from $GFP150TAG_{His6}$ in the presence of 2 mM substrate **1**. **e,** Raw mass spectrum for purified $GFP150BenzK_{His6}$ produced with PylRS **4**_1. **f,** Deconvoluted mass spectrum resulting from the primary spectrum shown in panel **e**. Mass predicted 27945.5 Da, mass found 27944.8 Da. The minor peak labeled -met corresponds to cleavage of the N-terminal methionine residue. **g-h**, same as **e** and **f** but for $GFP150BenzK_{His6}$ produced with PylRS **4**_2. Mass predicted 27945.5 Da, mass found 27944.8 Da. **i-j**, same as **e** and **f** but for $GFP150BenzK_{His6}$ produced with PylRS **4**_3. Mass predicted 27945.5 Da, mass found 27944.4 Da. **k-l**, same as **e** and **f** but for $GFP150BenzK_{His6}$ produced with PylRS **4**_4. Mass predicted 27945.5 Da, mass found 27944.4 Da.

a

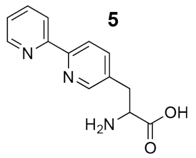

b

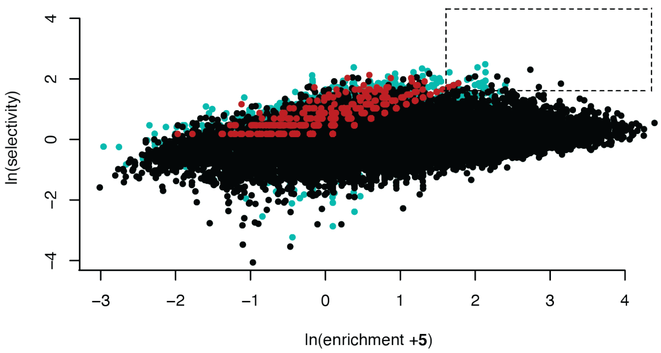

c

hit name	M300	A302	L305	Y306	L309	M344	N346	C348	Y384	S399	V401	selectivity	enrichment
5_1	M	A	P	Y	S	Q	C	P	F	S	T	12.0	8.4
	S	S	V	G	D	M	A	G	Y	S	F	10.8	7.6
5_2	M	A	M	Y	L	M	C	Y	F	S	V	10.0	15.4
5_3	D	H	G	Y	L	M	A	L	Y	S	T	9.2	8.5
5_4	K	R	G	V	T	M	N	C	Y	S	V	9.2	5.2
5_5	M	A	I	F	L	Q	G	V	L	T	A	8.0	7.4
5_6	M	H	L	S	L	M	C	L	Y	S	K	8.0	7.4
	C	H	I	Y	L	M	T	L	Y	S	K	8.0	7.4
	M	A	L	F	L	M	A	L	L	S	C	8.0	9.9
	N	N	L	L	L	M	F	C	Y	S	V	7.6	7.0
	H	P	P	P	A	M	N	C	Y	S	V	7.6	7.0
	M	A	L	H	M	M	T	F	Y	S	V	7.6	6.8
	S	H	I	Y	L	M	G	S	Y	S	V	7.2	6.6
	D	H	C	Y	L	M	G	I	Y	S	A	7.2	6.6
	D	H	C	F	L	M	A	F	Y	S	C	7.0	11.3
	S	H	H	Y	L	M	G	N	Y	S	V	7.0	8.5
	D	H	C	F	L	M	G	C	Y	S	A	6.9	8.5
	T	H	L	I	L	M	S	C	Y	S	V	6.5	8.1
	M	H	I	I	L	M	T	C	Y	S	C	6.5	8.1
	D	H	I	Y	L	M	T	S	Y	S	A	6.4	5.9
	M	A	L	K	V	M	R	M	Y	S	T	6.4	5.9
	M	A	T	Y	C	Q	T	P	Y	T	F	6.4	5.9
	D	S	C	F	G	M	A	F	Y	S	V	6.4	5.9
	M	H	L	Y	L	M	A	V	Y	S	T	6.3	7.7
	M	A	L	S	L	M	T	Y	Y	S	V	6.3	7.7

d

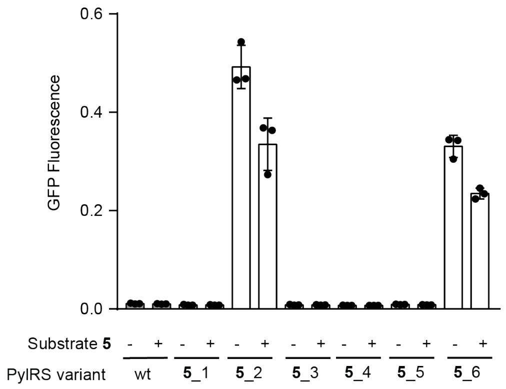

Supplementary Figure 15. Selection of PylRS variants for 3-([2,2'-bipyridin]-5-yl)-2-aminopropanoic acid (BiPyA) 5 by tRNA display.

a, Chemical structure of BiPyA **5**. **b,** Spindle plot resulting from the tRNA display selection as described in **Supplementary Figure 11**. The selectivity is defined as the ratio of the relative abundance of a particular sequence in the positive samples (+ **5**), divided by the relative abundance in the control sample (-ncAA). The enrichment is defined as the ratio of the same sequence in the positive samples, divided by the relative abundance in the input library. The boxed region indicates sequences with selectivities of ≥ 5, and enrichments of ≥ 5. Black dots are sequences observed in all positive samples and all control samples. Green dots are sequences observed in all positive samples, and at least one control sample. Red dots are sequences only observed in the positive samples. **c**, Top 25 sequences, ordered by selectivity, resulting from the selection for substrate **5** with selectivities of ≥ 5, and enrichments of ≥ 5. **d**, Amber suppressor activity data for selected PylRS variants measured by the

production of GFP150BiPyA$_{His6}$ from *GFP150TAG*$_{His6}$ from cells harboring a pMB1 plasmid encoding either wild type (wt) *Mm*PylRS or the indicated *Mm*PylRS variants, and a p15A plasmid encoding *GFP150TAG*$_{His6}$ in the presence and absence of 4 mM **5**. The fluorescence is shown relative to cells harboring wt *Mm*PylRS, *Mm*tRNA$^{Pyl}_{CUA}$ expressing GFP from *GFP150TAG*$_{His6}$ in the presence of 2 mM substrate **1**.

a

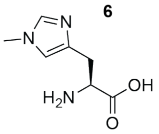

b

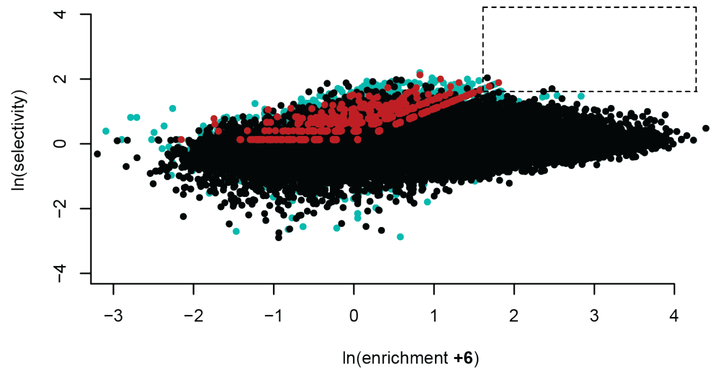

c

M300	A302	L305	Y306	L309	M344	N346	C348	Y384	S399	V401	selectivity	enrichment
M	A	L	K	K	Q	P	A	Y	S	W	7.7	5.3
R	V	S	V	P	M	N	C	Y	S	V	6.6	6.1
D	Q	L	M	L	M	G	C	Y	S	T	6.5	6.0
M	A	L	Y	Q	M	S	W	F	S	V	6.3	5.8
T	Y	L	T	L	M	G	C	Y	S	V	6.2	5.7
D	H	L	Y	L	M	A	L	Y	S	L	6.0	5.5
D	Q	L	F	V	M	G	C	X	S	V	5.9	5.4
T	F	L	H	F	M	V	C	Y	S	V	5.9	5.4
D	Q	L	S	L	M	G	C	Y	S	V	5.6	5.2
N	R	L	R	N	M	G	C	Y	S	V	5.6	5.2
D	Y	V	F	C	M	G	I	Y	S	V	5.5	11.9
M	A	L	R	R	Q	A	I	Y	S	F	5.3	5.0
D	N	V	G	Y	M	V	L	Y	S	K	5.3	5.1
M	A	L	P	R	M	T	E	Y	S	T	5.1	6.2
M	A	H	L	L	M	T	F	F	S	V	5.1	6.3
T	H	L	Y	L	M	E	Y	Y	S	V	5.0	10.7

Supplementary Figure 16. Selection of PylRS variants for *N*$^{\tau}$-methyl-*L*-histidine (N$^{\tau}$mH) 6 by tRNA display.

a, Chemical structure of N$^{\tau}$mH **6**. **b,** Spindle plot resulting from the tRNA display selection as described in **Supplementary Figure 11**. The selectivity is defined as the ratio of the relative abundance of a particular sequence in the positive samples (+ **6**), divided by the relative abundance in the control sample (-ncAA). The enrichment is defined as the ratio of the same sequence in the positive samples, divided by the relative abundance in the input library. The boxed region indicates sequences with selectivities of ≥ 5, and enrichments of ≥ 5. Black dots are sequences observed in all positive samples and all control samples. Green dots are sequences observed in all positive samples, and at least one control sample. Red dots are sequences only observed in the positive samples. **c**, The 16 sequences, ordered by selectivity, resulting from the selection for substrate **6** with selectivities of ≥ 5, and enrichments of ≥ 5.

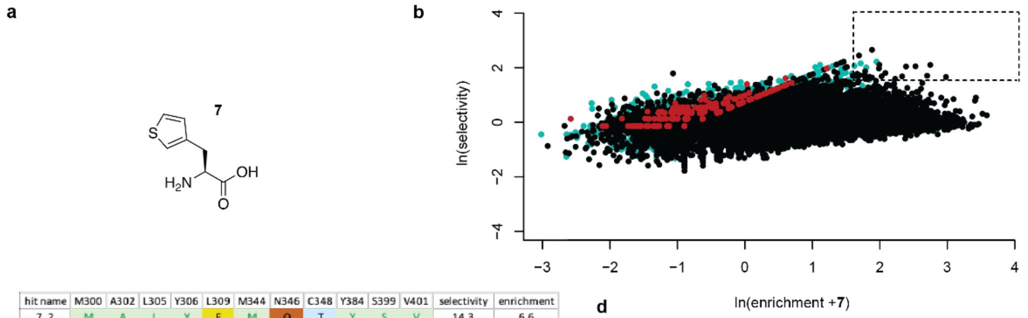

c

hit name	M300	A302	L305	Y306	L309	M344	N346	C348	Y384	S399	V401	selectivity	enrichment
7_2	M	A	L	Y	F	M	Q	T	Y	S	V	14.3	6.6
7_3	M	A	L	Y	L	M	Q	T	F	T	V	11.6	5.4
7_4	M	A	L	Y	S	M	Q	T	F	S	V	9.2	7.0
7_5	M	A	L	Y	I	M	Q	T	Y	S	V	8.7	5.6
7_1	M	A	V	Y	L	M	Q	T	Y	S	V	8.2	7.3
7_6	M	A	L	Y	F	M	Q	S	F	S	V	8.2	15.6
	M	D	L	R	M	M	Q	C	Y	S	V	7.9	5.1
7_7	M	A	L	Y	L	M	Q	T	F	S	V	7.8	12.5
7_8	M	A	L	Y	F	M	Q	T	F	S	V	7.7	10.9
	M	A	L	Y	V	M	Q	K	Y	S	V	7.4	6.0
	M	A	L	Y	F	M	Q	S	F	T	V	7.2	7.3
	M	A	L	Y	L	M	Q	T	Y	S	V	6.5	5.9
	M	A	L	Y	L	M	T	E	Y	S	C	6.1	8.0
	T	Q	L	Y	M	M	A	C	Y	S	V	5.9	5.3
	M	A	C	Y	L	M	T	K	Y	T	V	5.8	5.9
	M	A	L	Y	F	Q	V	K	Y	T	V	5.6	5.5
	M	A	L	Y	F	M	T	K	Y	S	V	5.5	14.9
	M	A	L	Y	V	M	Q	T	Y	S	V	5.5	5.2
	M	A	L	Y	L	M	Q	S	F	S	V	5.4	5.9
	M	H	L	Y	N	M	T	C	Y	S	V	5.4	7.6
	M	A	L	Y	C	M	Q	S	Y	S	V	5.4	10.3
	M	A	L	Y	L	M	Q	S	Y	S	V	5.3	6.3
	M	A	L	Y	L	M	T	K	Y	T	V	5.3	19.7
	M	A	L	Y	V	M	T	K	Y	S	V	5.2	6.6
	M	A	L	Y	C	M	Q	T	Y	S	V	5.1	6.4

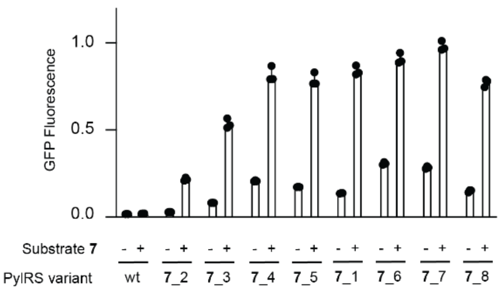

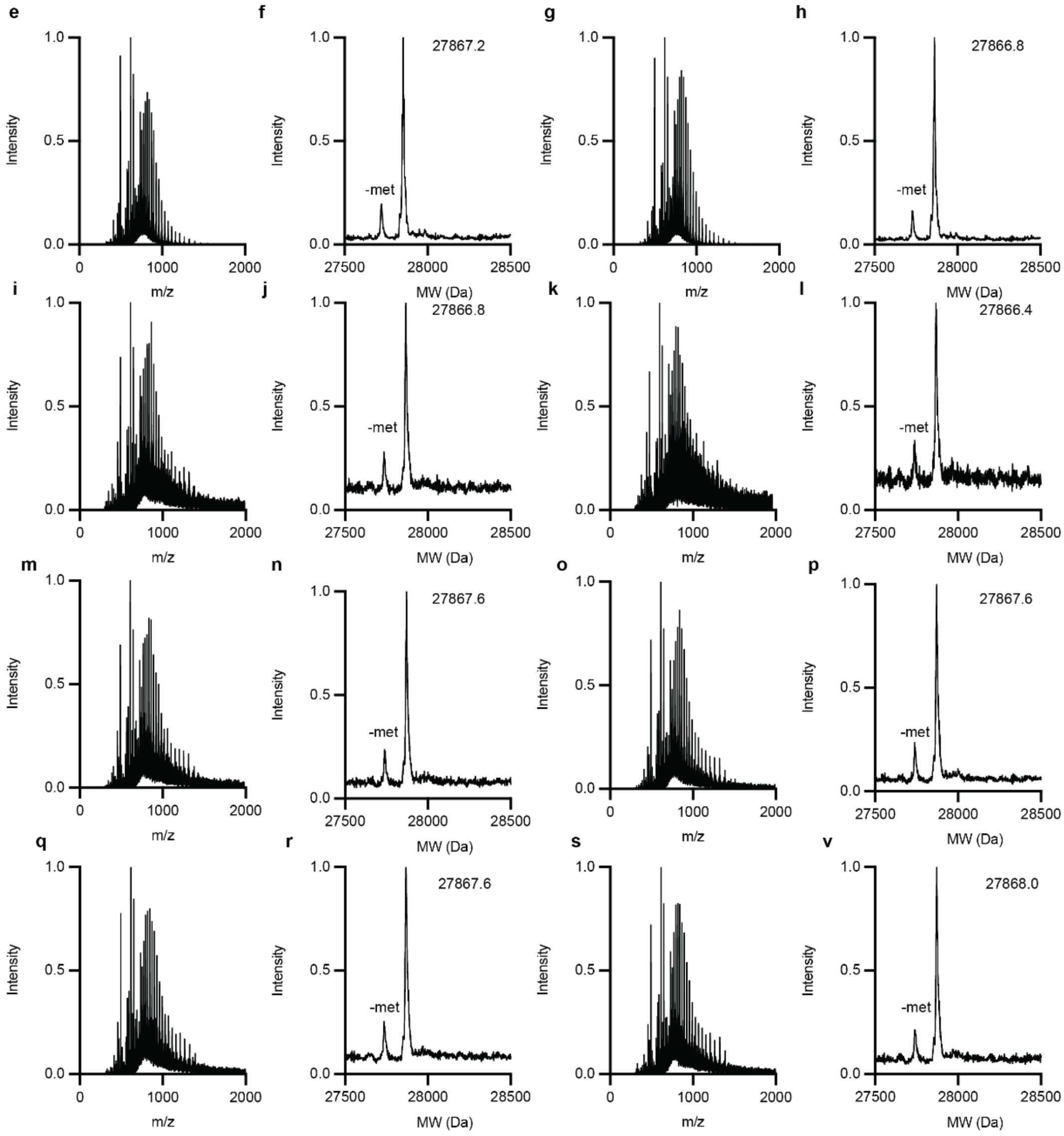

Supplementary Figure 17. Selection of PylRS variants for (*S*)-2-amino-3-(thiophen-3-yl)propanoic acid (3-ThiA) 7 by tRNA display.

a, Chemical structure of 3-ThiA **7**. **b,** Spindle plot resulting from the tRNA display selection as described in **Supplementary Figure 11**. The selectivity is defined as the ratio of the relative abundance of a particular sequence in the positive samples (+ **7**), divided by the relative abundance in the control sample (-ncAA). The enrichment is defined as the ratio of the same sequence in the positive samples, divided by the relative abundance in the input library. The boxed region indicates sequences with selectivities of ≥ 5, and enrichments of ≥ 5. Black dots are sequences observed in all positive samples and all control samples. Green dots are sequences observed in all positive samples, and at least one control sample. Red dots are sequences only observed in the positive samples. **c**, Top 25 sequences, ordered by selectivity, resulting from the selection for substrate **7** with selectivities of ≥ 5, and enrichments of ≥ 5. **d**, Amber suppressor activity data for selected PylRS variants measured by the production of GFP150-3-ThiA $_{\mathrm{His6}}$ from *GFP150TAG$_{His6}$* from cells harboring a pMB1 plasmid encoding either wild type (wt) *Mm*PylRS or the indicated *Mm*PylRS variants, and a p15A plasmid encoding *GFP150TAG$_{His6}$* in the presence and absence of 4 mM **7**. The fluorescence is shown relative to cells harboring wt *Mm*PylRS, *Mm*tRNA$^{\mathrm{Pyl}}_{\mathrm{CUA}}$ expressing GFP from *GFP150TAG$_{His6}$* in the presence of 2 mM substrate **1**. **e,** Raw mass spectrum for purified GFP150-3-ThiA K$_{\mathrm{His6}}$ produced with PylRS **7**_2. **f,** Deconvoluted mass spectrum resulting from the primary spectrum shown in panel **e**. Mass predicted 27866.4 Da, mass found 27867.2 Da. The minor peak labeled -met corresponds to cleavage of the N-terminal methionine residue. **g-h**, same as **e** and **f** but for GFP150-3-ThiA$_{\mathrm{His6}}$ produced with PylRS **7**_3. Mass predicted 27866.4 Da, mass found 27866.8 Da. **i-j**, same as **e** and **f** but for GFP150-3-ThiA$_{\mathrm{His6}}$ produced with PylRS **7**_4. Mass predicted 27866.4 Da, mass found 27866.8 Da. **k-l**, same as **e** and **f** but for GFP150-3-ThiA$_{\mathrm{His6}}$ produced with PylRS **7**_5. Mass predicted 27866.4 Da, mass found 27866.4 Da. **m-n**, same as **e** and **f** but for GFP150-3-ThiA$_{\mathrm{His6}}$ produced with PylRS **7**_1. Mass predicted 27866.4 Da, mass found 27867.6 Da. **o-p**, same as **e** and **f** but for GFP150-3-ThiA$_{\mathrm{His6}}$ produced with PylRS **7**_6. Mass predicted 27866.4 Da, mass found 27867.6 Da. **q-r**, same as **e** and **f** but for GFP150-3-ThiA$_{\mathrm{His6}}$ produced with PylRS **7**_7. Mass predicted 27866.4 Da, mass found 27867.6Da. **s-t**, same as **e** and **f** but for GFP150-3-ThiA $_{\mathrm{His6}}$ produced with PylRS **7**_8. Mass predicted 27866.4 Da, mass found 27868.0 Da.

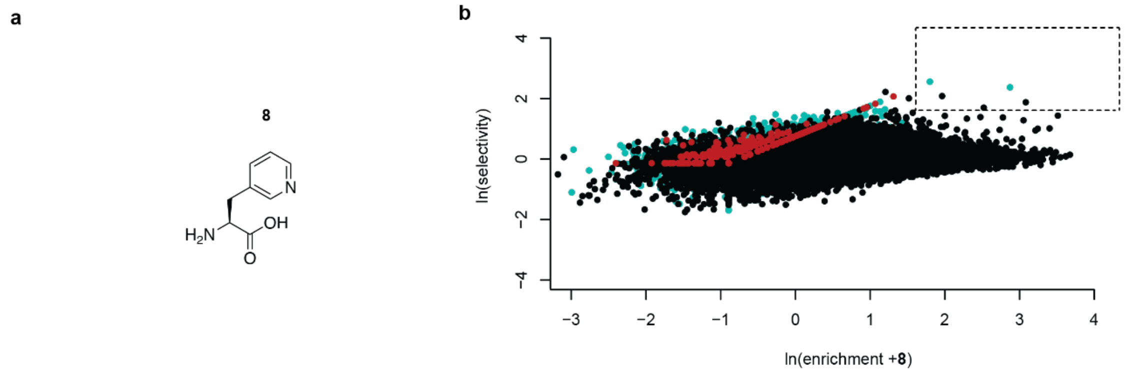

c

hit name	M300	A302	L305	Y306	L309	M344	N346	C348	Y384	S399	V401	selectivity	enrichment
8_2	M	A	L	M	L	M	S	E	Y	S	V	12.9	0.5
8_1	S	H	L	Y	L	M	S	E	Y	S	V	10.7	1.7
8_3	A	H	L	Y	L	M	A	E	Y	S	V	8.0	0.9
	L	H	L	Y	L	M	A	E	Y	S	V	6.5	3.3
8_5	T	H	L	Y	L	Q	A	E	Y	S	V	5.5	2.3

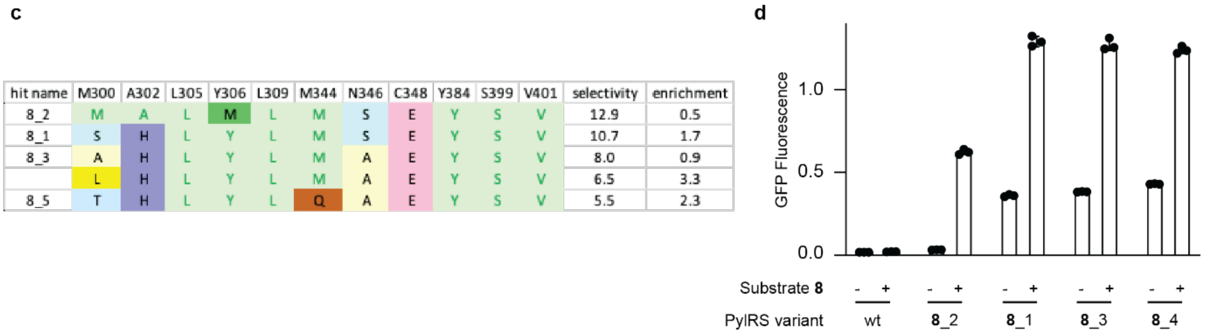

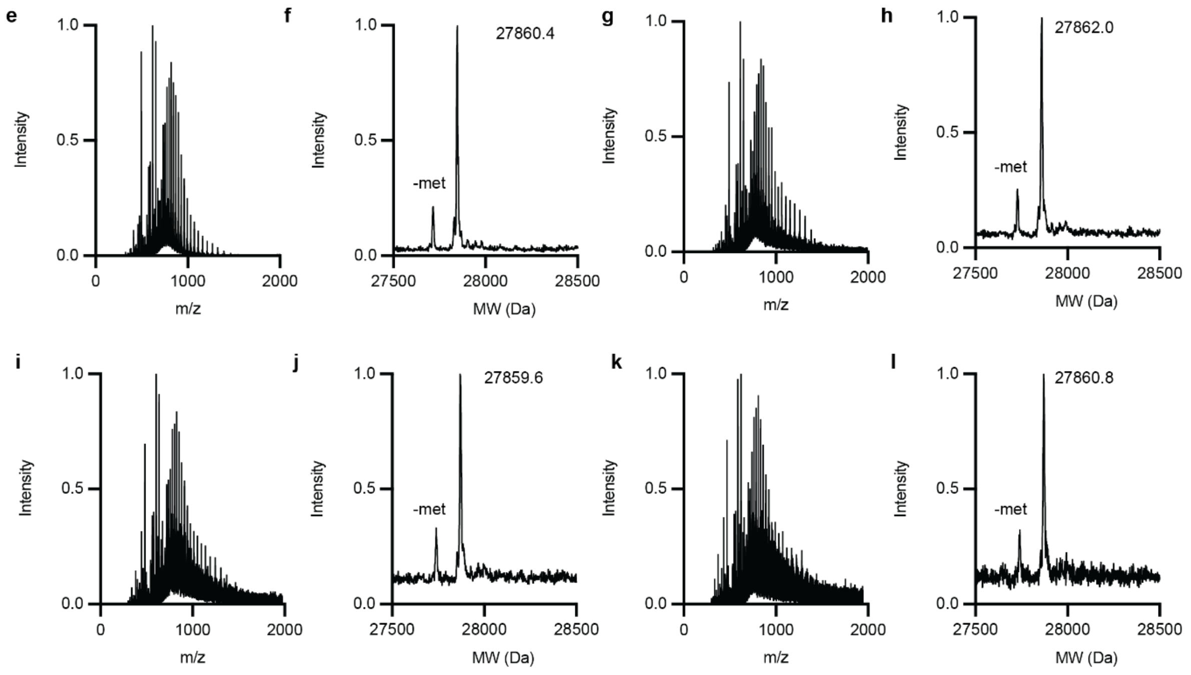

Supplementary Figure 18. Selection of PylRS variants for (*S*)-2-amino-3-(pyridin-3-yl)propanoic acid (PyA) 8 by tRNA display.

a, Chemical structure of PyA **8**. **b,** Spindle plot resulting from the tRNA display selection as described in **Supplementary Figure 11**. The selectivity is defined as the ratio of the relative abundance of a particular sequence in the positive samples (+ **8**), divided by the relative abundance in the control sample

(-ncAA). The enrichment is defined as the ratio of the same sequence in the positive samples, divided by the relative abundance in the input library. The boxed region indicates sequences with selectivities of ≥ 5, and enrichments of ≥ 5. Black dots are sequences observed in all positive samples and all control samples. Green dots are sequences observed in all positive samples, and at least one control sample. Red dots are sequences only observed in the positive samples. **c**, The five sequences, ordered by selectivity, resulting from the selection for substrate **8** with selectivities of ≥ 5, and enrichments of ≥ 5. **d**, Amber suppressor activity data for selected PylRS variants measured by the production of GFP150PyA$_{\text{His6}}$ from *GFP150TAG*$_{His6}$ from cells harboring a pMB1 plasmid encoding either wild type (wt) *Mm*PylRS or the indicated *Mm*PylRS variants, and a p15A plasmid encoding *GFP150TAG*$_{His6}$ in the presence and absence of 4 mM **8**. The fluorescence is shown relative to cells harboring wt *Mm*PylRS, *Mm*tRNA$^{\text{Pyl}}_{\text{CUA}}$ expressing GFP from *GFP150TAG*$_{His6}$ in the presence of 2 mM substrate **1**. **e,** Raw mass spectrum for purified GFP150PyA$_{\text{His6}}$ produced with PylRS **8**_2. **f,** Deconvoluted mass spectrum resulting from the primary spectrum shown in panel **e**. Mass predicted 27861.4 Da, mass found 27860.4 Da. The minor peak labeled -met corresponds to cleavage of the N-terminal methionine residue. **g-h**, same as **e** and **f** but for GFP150PyA$_{\text{His6}}$ produced with PylRS **8**_1. Mass predicted 27861.4 Da, mass found 27862.0 Da. **i-j**, same as **e** and **f** but for GFP150PyA$_{\text{His6}}$ produced with PylRS **8**_3. Mass predicted 27861.4 Da, mass found 27859.6 Da. **k-l**, same as **e** and **f** but for GFP150PyA$_{\text{His6}}$ produced with PylRS **8**_4. Mass predicted 27861.4 Da, mass found 27860.8 Da.

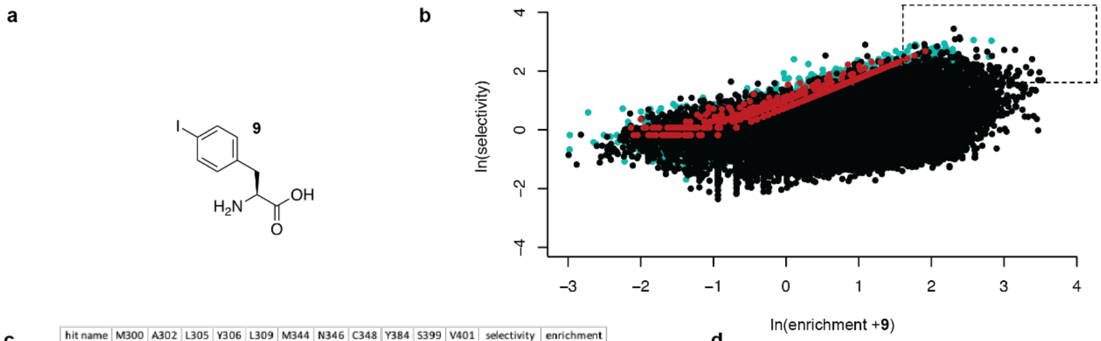

c

hit name	M300	A302	L305	Y306	L309	M344	N346	C348	Y384	S399	V401	selectivity	enrichment
9_2	M	H	L	L	L	M	G	L	Y	S	K	31.1	10.0
9_3	M	A	C	Y	V	M	G	L	Y	S	V	23.2	10.9
9_1	S	H	H	L	L	M	G	I	Y	S	V	22.0	10.9
9_4	M	H	L	S	F	M	G	C	Y	S	V	21.8	10.2
9_5	M	H	H	L	L	M	G	V	Y	S	V	21.2	13.2
9_6	M	A	L	F	S	Q	S	F	Y	S	V	20.7	16.9
	M	A	L	F	S	Q	S	H	Y	S	V	18.8	8.8
	M	A	L	L	A	M	S	F	Y	S	K	18.5	6.7
	L	H	L	F	L	M	C	C	Y	S	V	18.3	5.9
	L	Q	L	F	F	M	G	C	Y	S	V	17.4	6.7
	M	A	L	L	G	M	S	F	Y	S	V	17.4	6.0
	M	A	F	Y	L	M	C	Q	Y	S	V	17.2	5.9
	M	A	V	F	L	Q	G	V	F	T	V	17.1	8.0
	M	A	L	N	A	M	S	F	Y	S	V	17.1	5.5
	T	W	L	F	N	M	G	C	Y	S	V	17.0	8.0
	M	A	L	Y	G	M	S	F	Y	S	V	16.7	9.0
	S	H	H	L	L	M	G	N	Y	S	V	16.5	7.7
	M	A	V	Y	G	M	G	F	Y	S	V	16.3	7.7
	M	A	L	L	G	M	C	F	Y	S	V	16.3	6.8
	M	A	L	V	A	M	S	F	Y	S	V	16.3	6.8
	M	A	L	T	A	M	S	F	Y	S	V	16.3	8.1
	M	A	L	V	G	M	A	Y	Y	S	V	15.9	8.9
	T	M	L	L	L	M	C	C	Y	S	V	15.9	7.9
	D	C	C	F	V	M	G	L	Y	S	A	15.8	7.4
	M	A	L	N	A	M	C	F	Y	S	V	15.5	9.9

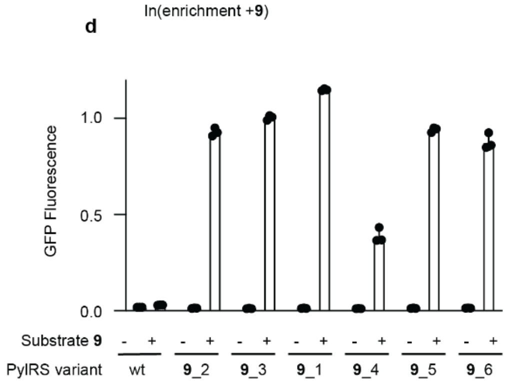

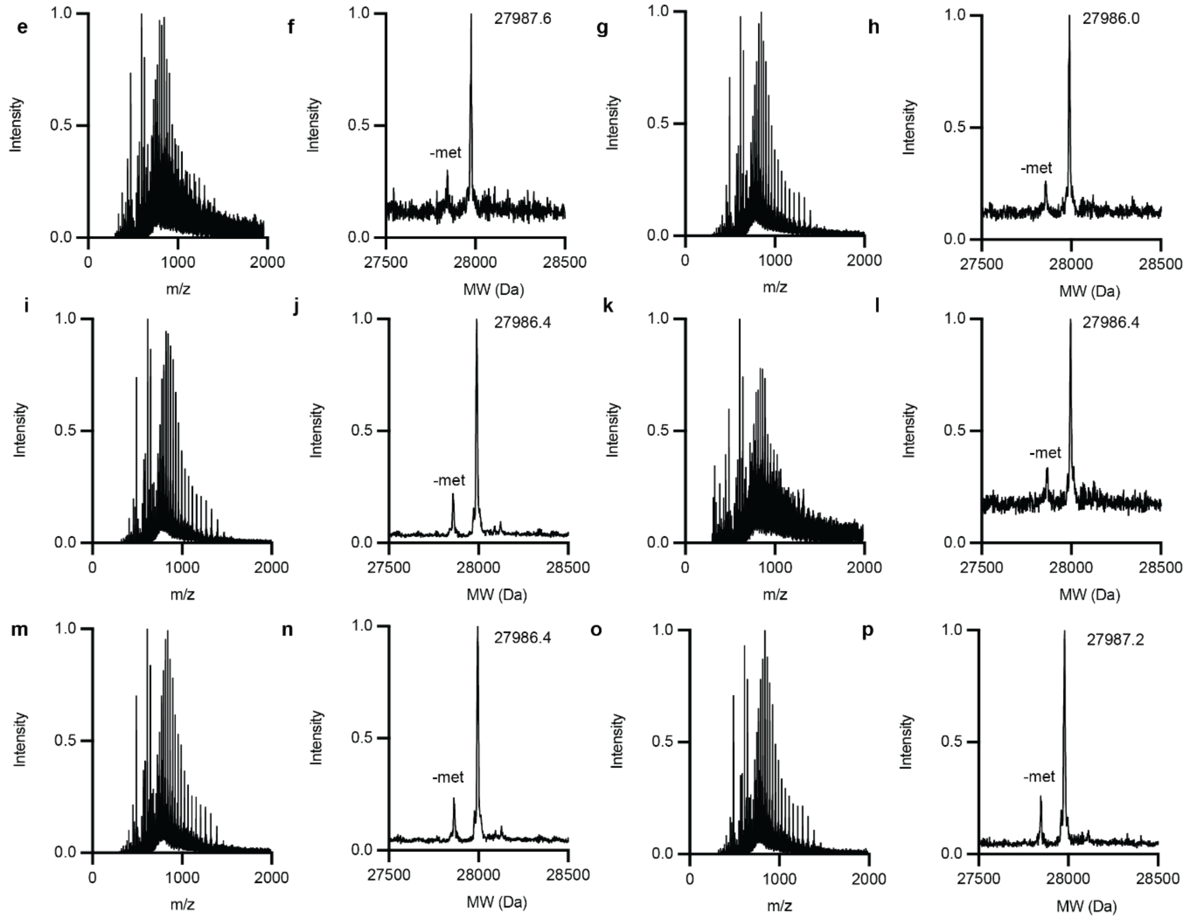

Supplementary Figure 19. Selection of PylRS variants for (*S*)-2-amino-3-(4-iodophenyl)propanoic acid (pIF) 9 by tRNA display.

a, Chemical structure of pIF **9**. **b,** Spindle plot resulting from the tRNA display selection as described in **Supplementary Figure 11**. The selectivity is defined as the ratio of the relative abundance of a particular sequence in the positive samples (+ **9**), divided by the relative abundance in the control sample (-ncAA). The enrichment is defined as the ratio of the same sequence in the positive samples, divided by the relative abundance in the input library. The boxed region indicates sequences with selectivities of ≥ 5, and enrichments of ≥ 5. Black dots are sequences observed in all positive samples and all control samples. Green dots are sequences observed in all positive samples, and at least one control sample. Red dots are sequences only observed in the positive samples. **c**, Top 25 sequences, ordered by selectivity, resulting from the selection for substrate **9** with selectivities of ≥ 5, and enrichments of ≥ 5. **d**, Amber suppressor activity data for selected PylRS variants measured by the production of $GFP150pIF_{His6}$ from $\mathit{GFP150TAG}_{His6}$ from cells harboring a pMB1 plasmid encoding either wild type (wt) *Mm*PylRS or the indicated *Mm*PylRS variants, and a p15A plasmid encoding $\mathit{GFP150TAG}_{His6}$ in the presence and absence of 4 mM **9**. The fluorescence is shown relative to cells harboring wt *Mm*PylRS, *Mm*$tRNA^{Pyl}_{CUA}$ expressing GFP from $\mathit{GFP150TAG}_{His6}$ in the presence of 2 mM substrate **1**. **e,** Raw mass spectrum for purified $GFP150pIFK_{His6}$ produced with PylRS **9**_2. **f,** Deconvoluted mass spectrum resulting from the primary spectrum shown in panel **e**. Mass predicted 27986.2 Da, mass found 27987.6 Da. The minor peak labeled -met corresponds to cleavage of the N-terminal methionine residue. **g-h**, same as **e** and **f** but for $GFP150pIFK_{His6}$ produced with PylRS **9**_3. Mass predicted 27986.2 Da, mass found 27986.0 Da. **i-j**, same as **e** and **f** but for $GFP150pIFK_{His6}$ produced with PylRS **9**_1. Mass predicted 27986.2 Da, mass found 27986.4 Da. **k-l**, same as **e** and **f** but for $GFP150pIFK_{His6}$ produced with PylRS **9**_4. Mass predicted 27986.2 Da, mass found 27986.4 Da. **m-n**, same as **e** and **f** but for $GFP150pIFK_{His6}$ produced with PylRS **9**_5. Mass predicted 27986.2 Da, mass found 27986.4 Da. **o-p**, same as **e** and **f** but for $GFP150pIFK_{His6}$ produced with PylRS **9**_6. Mass predicted 27986.2 Da, mass found 27987.2 Da.

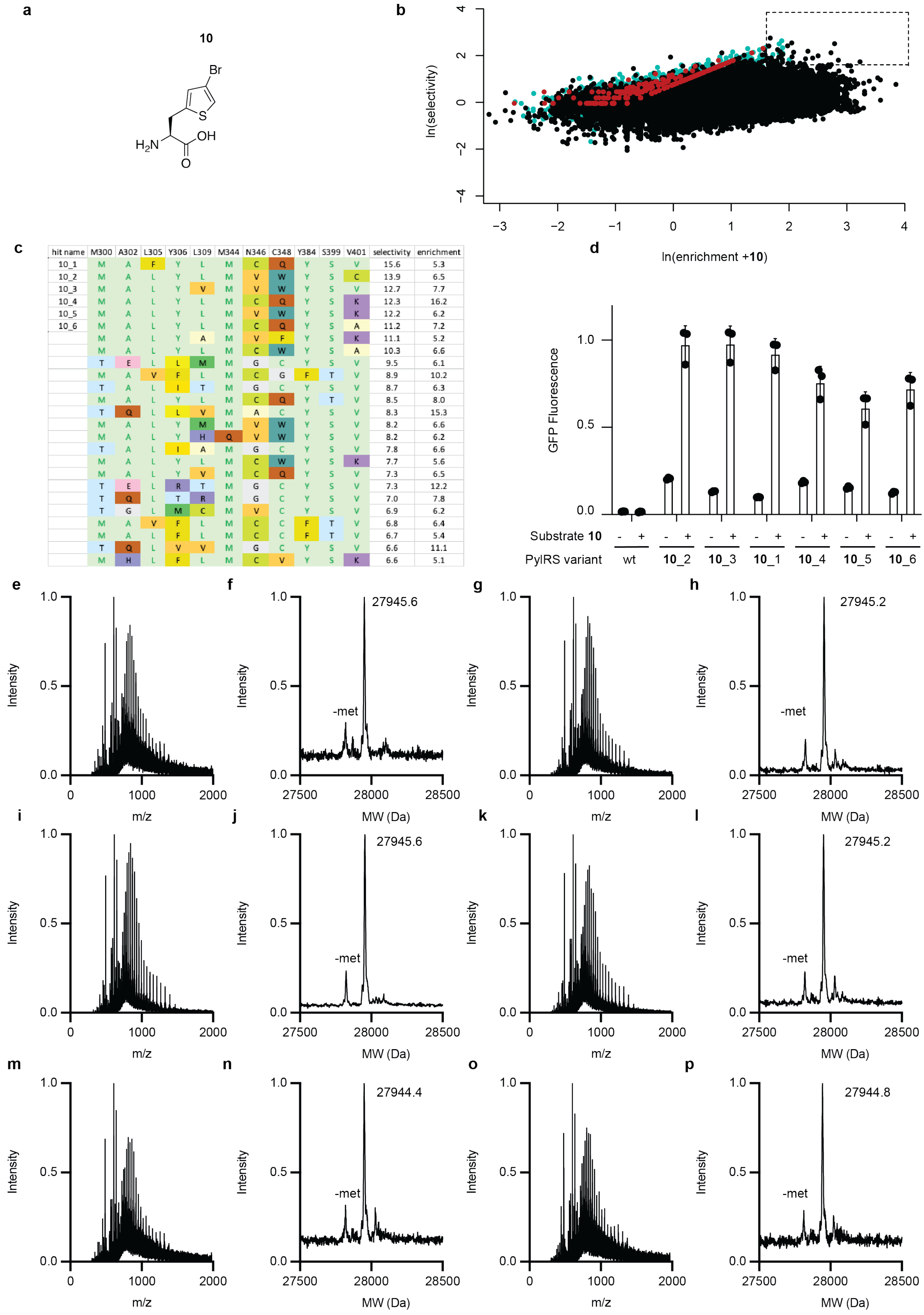

hit name	M300	A302	L305	Y306	L309	M344	N346	C348	Y384	S399	V401	selectivity	enrichment
10_1	M	A	F	Y	L	M	C	Q	Y	S	V	15.6	5.3
10_2	M	A	L	Y	L	M	V	W	Y	S	C	13.9	6.5
10_3	M	A	L	Y	V	M	V	W	Y	S	V	12.7	7.7
10_4	M	A	L	Y	L	M	C	Q	Y	S	K	12.3	16.2
10_5	M	A	L	Y	L	M	V	W	Y	S	K	12.2	6.2
10_6	M	A	L	Y	L	M	C	Q	Y	S	A	11.2	7.2
	M	A	L	Y	A	M	V	F	Y	S	K	11.1	5.2
	M	A	L	Y	L	M	C	W	Y	S	A	10.3	6.6
	T	E	L	L	M	M	G	C	Y	S	V	9.5	6.1
	M	A	V	F	L	M	C	G	F	T	V	8.9	10.2
	T	A	L	I	T	M	G	C	Y	S	V	8.7	6.3
	M	A	L	Y	L	M	C	Q	Y	T	V	8.5	8.0
	T	Q	L	L	V	M	A	C	Y	S	V	8.3	15.3
	M	A	L	Y	M	M	V	W	Y	S	V	8.2	6.6
	M	A	L	Y	H	Q	V	W	Y	S	V	8.2	6.2
	T	A	L	I	A	M	G	C	Y	S	V	7.8	6.6
	M	A	L	Y	L	M	C	W	Y	S	K	7.7	5.6
	M	A	L	Y	V	M	C	Q	Y	S	V	7.3	6.5
	T	E	L	R	T	M	G	C	Y	S	V	7.3	12.2
	T	Q	L	T	R	M	G	C	Y	S	V	7.0	7.8
	T	G	L	M	C	M	V	C	Y	S	V	6.9	6.2
	M	A	V	F	L	M	C	C	F	T	V	6.8	6.4
	M	A	L	F	L	M	C	C	F	T	V	6.7	5.4
	T	Q	L	V	V	M	G	C	Y	S	V	6.6	11.1
	M	H	L	F	L	M	C	V	Y	S	K	6.6	5.1

Supplemenatry Figure 20. Selection of PylRS variants for (*S*)-2-amino-3-(4-bromothiophen-2-yl)propanoic acid (BrThiA) 10 by tRNA display.

a, Chemical structure of BrThiA **10**. **b,** Spindle plot resulting from the tRNA display selection as described in **Supplementary Figure 11**. The selectivity is defined as the ratio of the relative abundance of a particular sequence in the positive samples (+ **10**), divided by the relative abundance in the control sample (-ncAA). The enrichment is defined as the ratio of the same sequence in the positive samples, divided by the relative abundance in the input library. The boxed region indicates sequences with selectivities of ≥ 5, and enrichments of ≥ 5. Black dots are sequences observed in all positive samples and all control samples. Green dots are sequences observed in all positive samples, and at least one control sample. Red dots are sequences only observed in the positive samples. **c**, Top 25 sequences, ordered by selectivity, resulting from the selection for substrate **10** with selectivities of ≥ 5, and enrichments of ≥ 5. **d**, Amber suppressor activity data for selected PylRS variants measured by the production of GFP150BrThiA$_{\text{His6}}$ from $\textit{GFP150TAG}_{\textit{His6}}$ from cells harboring a pMB1 plasmid encoding either wild type (wt) *Mm*PylRS or the indicated *Mm*PylRS variants, and a p15A plasmid encoding $\textit{GFP150TAG}_{\textit{His6}}$ in the presence and absence of 4 mM **10**. The fluorescence is shown relative to cells harboring wt *Mm*PylRS, *Mm*tRNA$^{\text{Pyl}}_{\text{CUA}}$ expressing GFP from $\textit{GFP150TAG}_{\textit{His6}}$ in the presence of 2 mM substrate **1**. **e,** Raw mass spectrum for purified GFP150BrThiA$_{\text{His6}}$ produced with PylRS **10**_2. **f,** Deconvoluted mass spectrum resulting from the primary spectrum shown in panel **e**. Mass predicted 27944.3Da, mass found 27945.6 Da. The minor peak labeled -met corresponds to cleavage of the N-terminal methionine residue. **g-h**, same as **e** and **f** but for GFP150BrThiA$_{\text{His6}}$ produced with PylRS **10**_3. Mass predicted 27944.3Da, mass found 27945.2 Da. **i-j**, same as **e** and **f** but for GFP150BrThiA$_{\text{His6}}$ produced with PylRS **10**_1. Mass predicted 27944.3 Da, mass found 27945.6 Da. **k-l**, same as **e** and **f** but for GFP150BrThiA$_{\text{His6}}$ produced with PylRS **10**_4. Mass predicted 27944.3Da, mass found 27945.2 Da. **m-n**, same as **e** and **f** but for GFP150BrThiA$_{\text{His6}}$ produced with PylRS **10**_5. Mass predicted 27944.3Da, mass found 27944.4 Da. **o-p**, same as **e** and **f** but for GFP150BrThiA$_{\text{His6}}$ produced with PylRS **10**_6. Mass predicted 27944.3Da, mass found 27944.8 Da.

a

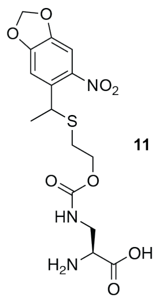

11

b

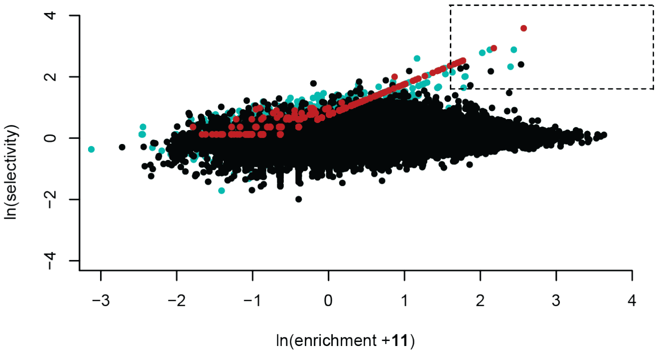

c

hit name	M300	A302	L305	Y306	L309	M344	N346	C348	Y384	S399	V401	selectivity	enrichment
11_2	M	A	V	V	L	M	Q	S	F	T	V	36.0	13.1
11_3	M	A	L	V	L	M	Q	S	F	S	V	18.8	8.8
11_1	M	A	V	V	L	M	Q	S	F	S	V	17.9	11.5
11_4	M	A	V	V	L	M	Q	S	Y	S	V	17.8	8.4
	M	A	V	V	L	M	Q	S	F	S	K	16.1	7.6
	M	A	V	I	L	M	Q	S	F	T	V	12.5	5.9
	M	A	L	L	L	M	Q	C	F	S	K	12.5	5.9
	M	A	L	I	L	M	Q	S	F	S	S	12.3	5.7
	M	A	V	L	L	M	Q	S	F	S	V	12.2	5.7
	M	A	V	L	L	M	Q	S	Y	S	V	12.0	5.6
	M	A	L	I	L	M	Q	S	F	T	V	11.8	5.5
	M	A	V	I	L	M	Q	S	F	S	V	11.6	5.4
	M	A	V	V	L	M	Q	A	F	S	K	11.2	5.3
11_5	M	A	L	A	L	M	G	G	L	S	K	11.1	12.6
	M	A	V	V	L	M	Q	S	F	S	S	10.8	5.1
	M	A	I	V	L	M	Q	S	F	S	V	10.8	5.1
	M	A	L	A	L	M	G	G	F	S	V	10.3	6.1
	M	A	X	A	L	M	G	G	L	S	K	10.3	11.0
	M	A	L	A	L	M	G	G	L	S	V	9.7	5.7
	M	A	L	A	L	M	G	G	Y	S	V	8.8	8.5
	M	A	L	V	L	M	Q	S	Y	S	V	8.6	5.1
	M	A	L	Y	S	M	S	G	F	S	V	7.5	6.1
	M	A	L	A	L	M	G	G	F	T	V	7.4	6.0
	M	A	L	V	L	M	S	G	L	S	K	5.6	6.5
	M	A	L	A	L	M	G	G	Y	S	T	5.2	5.9

d

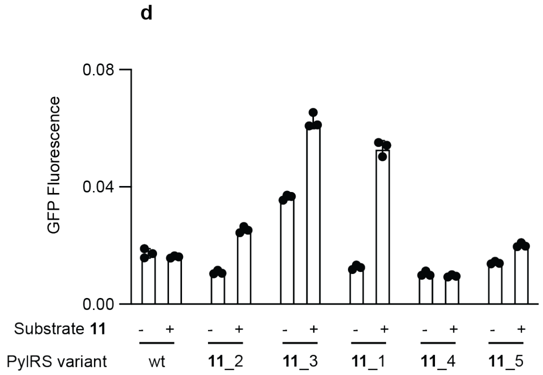

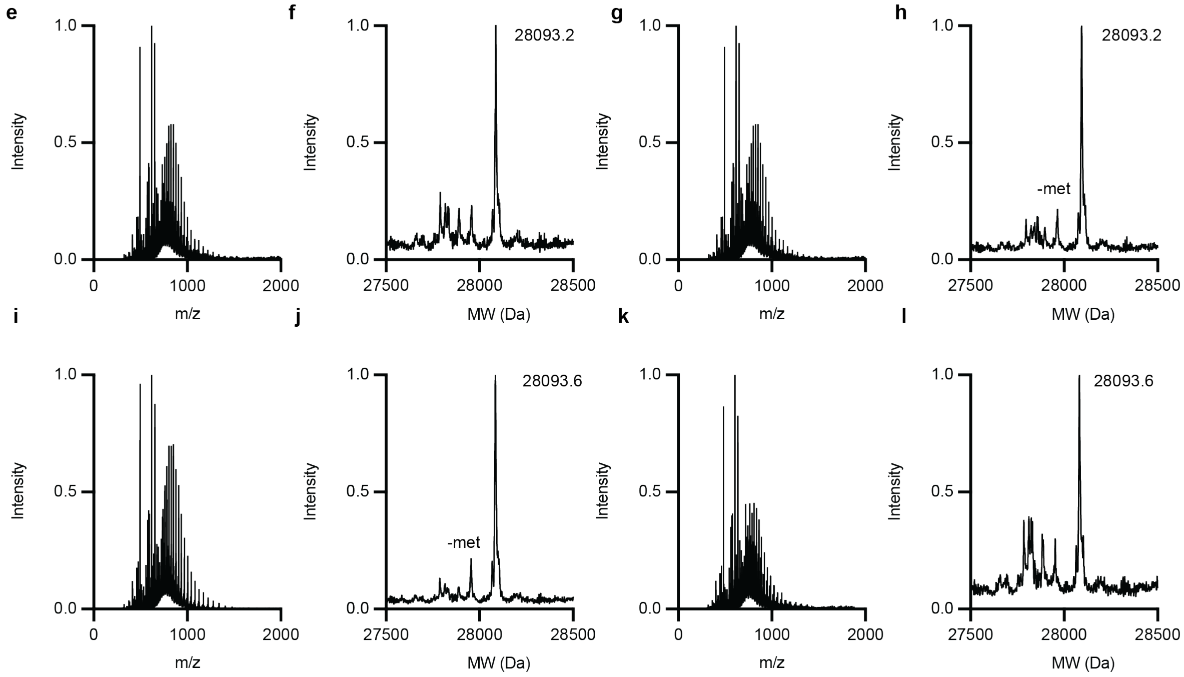

Supplementary Figure 21. Selection of PylRS variants for (2*S*)-2-amino-3-(((2-((1-(6-nitrobenzo[*d*][1,3]dioxol-5yl)ethyl)thio)ethoxy)carbonyl)amino)propanoic acid (pcDAP) 11 by tRNA display.

a, Chemical structure of pcDAP **11**. **b,** Spindle plot resulting from the tRNA display selection as described in **Supplementary Figure 11**. The selectivity is defined as the ratio of the relative abundance of a particular sequence in the positive samples (+ **11**), divided by the relative abundance in the control sample (-ncAA). The enrichment is defined as the ratio of the same sequence in the positive samples, divided by the relative abundance in the input library. The boxed region indicates sequences with selectivities of ≥ 5, and enrichments of ≥ 5. Black dots are sequences observed in all positive samples and all control samples. Green dots are sequences observed in all positive samples, and at least one control sample. Red dots are sequences only observed in the positive samples. **c**, Top 25 sequences, ordered by selectivity, resulting from the selection for substrate **11** with selectivities of ≥ 5, and enrichments of ≥ 5. **d**, Amber suppressor activity data for selected PylRS variants measured by the production of GFP150pcDAP$_{\mathrm{His6}}$ from *GFP150TAG*$_{His6}$ from cells harboring a pMB1 plasmid encoding either wild type (wt) *Mm*PylRS or the indicated *Mm*PylRS variants, and a p15A plasmid encoding *GFP150TAG*$_{His6}$ in the presence and absence of 4 mM **11**. The fluorescence is shown relative to cells harboring wt *Mm*PylRS, *Mm*tRNA$^{\mathrm{Pyl}}_{\mathrm{CUA}}$ expressing GFP from *GFP150TAG*$_{His6}$ in the presence of 2 mM substrate **1**. **e,** Raw mass spectrum for purified GFP150pcDAP$_{\mathrm{His6}}$ produced with PylRS **11**_2. **f,** Deconvoluted mass spectrum resulting from the primary spectrum shown in panel **e**. Mass predicted 28096.4 Da, mass found 28093.2 Da. The minor peak labeled -met corresponds to cleavage of the N-terminal methionine residue. **g-h**, same as **e** and **f** but for GFP150pcDAP$_{\mathrm{His6}}$ produced with PylRS **11**_3. Mass predicted 28096.4 Da, mass found 28096.4 Da. **i-j**, same as **e** and **f** but for GFP150pcDAP$_{\mathrm{His6}}$ produced with PylRS **11**_1. Mass predicted 28096.4 Da, mass found 28097.2 Da. **k-l**, same as **e** and **f** but for GFP150pcDAP$_{\mathrm{His6}}$ produced with PylRS **11**_5. Mass predicted 28096.4 Da, mass found 28093.6 Da.

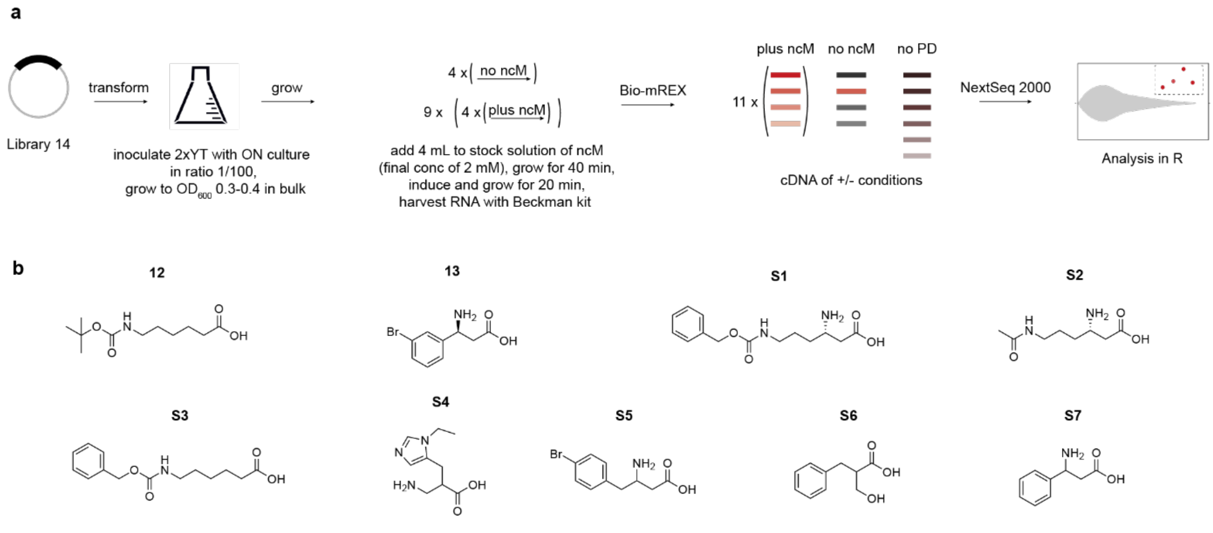

Supplementary Figure 22. Overview of one step tRNA display selection for non-canonical monomers.

a, Schematic representation of selection strategy for non-canonical monomers. Library 14 was transformed into BL21 cells and grown overnight. Cells were grown to OD_{600} of 0.3-0.4. 4 mL of the library culture was added into stock solutions of each ncM. 4 mL of the library culture was also added to a well without ncM. The cells were grown for 40 min, stmRNAs induced, and cells grown for another 20 min and the RNA was isolated. Bio-mREX was performed on the isolated RNA for each sample. The experiment was performed in 4 replicates, leading to 40 cDNA samples. An additional 6 cDNA samples were generated for 6 of the RNA inputs to bio-mREX. The resulting 46 cDNA samples were sequenced by NGS and analyzed to generate spindle plots and sequence tables. **b,** Structures of non-canonical monomers used in this selection. BocAhx (**12**), (*S*)-3-amino-3-(3-bromophenyl)propanoic acid ((*S*)β^3mBrF) (**13**), (*S*)-3-amino-6-(((benzyloxy)carbonyl)amino)hexanoic acid ((*S*)-β^3CbzK) (**S1**), (*S*)-6-acetamido-3-aminohexanoic acid ((*S*)-β^3AcK) (**S2**), 6-(((benzyloxy)carbonyl)amino)hexanoic acid (CbzAhx) (**S3**), 3-amino-2-((1-ethyl-1*H*-imidazol-5-yl)methyl)propanoic acid (β^2NeH (**S4**)), 3-amino-4-(4-bromophenyl)butanoic acid (β^3pBrhF) (**S5**), 2-benzyl-3-hydroxypropanoic acid (β^2OH-F) (**S6**), 3-amino-3-phenylpropanoic acid (β^3F) (**S7**).

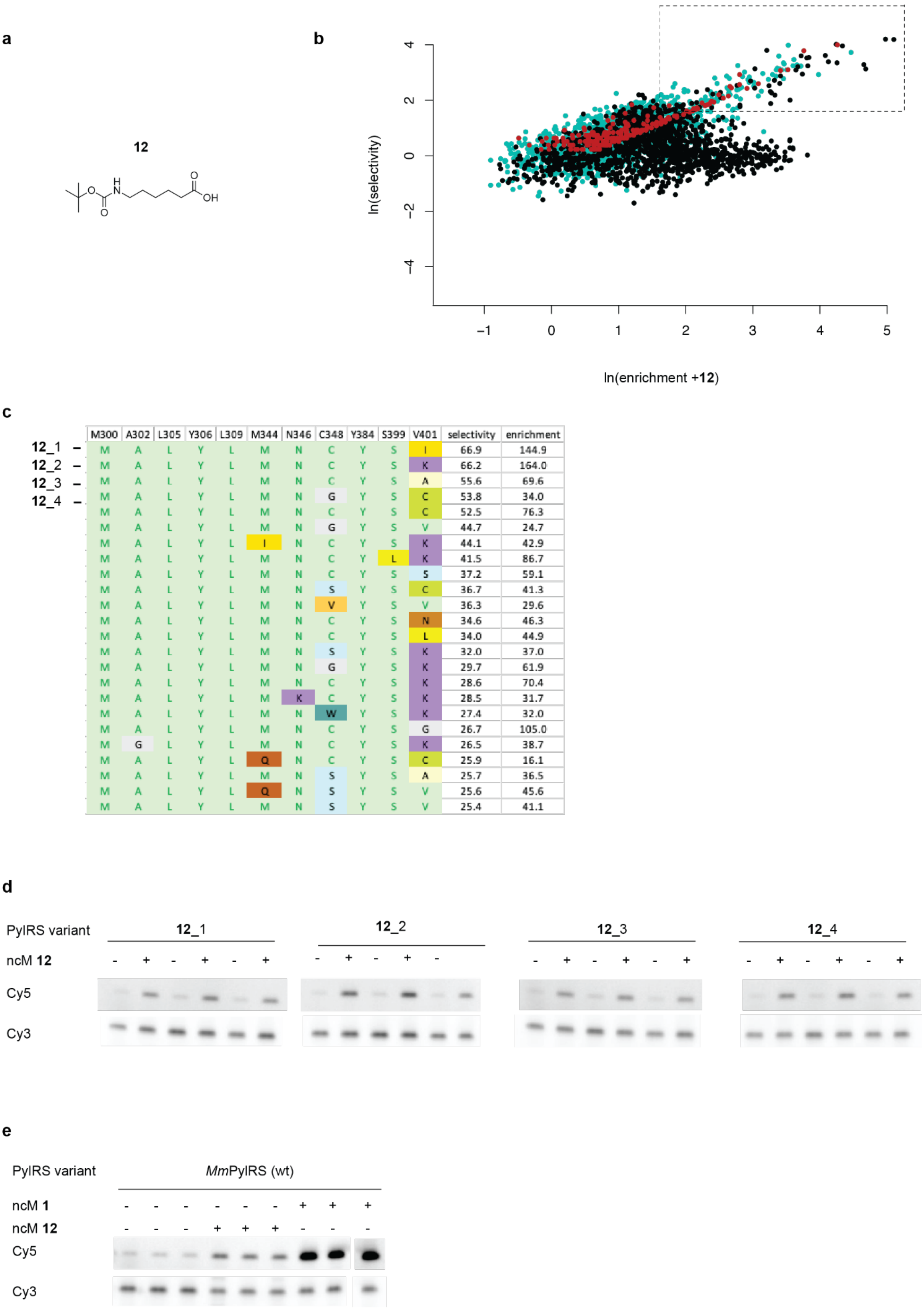

	M300	A302	L305	Y306	L309	M344	N346	C348	Y384	S399	V401	selectivity	enrichment
12_1	M	A	L	Y	L	M	N	C	Y	S	I	66.9	144.9
12_2	M	A	L	Y	L	M	N	C	Y	S	K	66.2	164.0
12_3	M	A	L	Y	L	M	N	C	Y	S	A	55.6	69.6
12_4	M	A	L	Y	L	M	N	G	Y	S	C	53.8	34.0
	M	A	L	Y	L	M	N	C	Y	S	C	52.5	76.3
	M	A	L	Y	L	M	N	G	Y	S	V	44.7	24.7
	M	A	L	Y	L	I	N	C	Y	S	K	44.1	42.9
	M	A	L	Y	L	M	N	C	Y	L	K	41.5	86.7
	M	A	L	Y	L	M	N	C	Y	S	S	37.2	59.1
	M	A	L	Y	L	M	N	S	Y	S	C	36.7	41.3
	M	A	L	Y	L	M	N	V	Y	S	V	36.3	29.6
	M	A	L	Y	L	M	N	C	Y	S	N	34.6	46.3
	M	A	L	Y	L	M	N	C	Y	S	L	34.0	44.9
	M	A	L	Y	L	M	N	S	Y	S	K	32.0	37.0
	M	A	L	Y	L	M	N	G	Y	S	K	29.7	61.9
	M	A	L	Y	L	M	N	C	Y	S	K	28.6	70.4
	M	A	L	Y	L	M	K	C	Y	S	K	28.5	31.7
	M	A	L	Y	L	M	N	W	Y	S	K	27.4	32.0
	M	A	L	Y	L	M	N	C	Y	S	G	26.7	105.0
	M	G	L	Y	L	M	N	C	Y	S	K	26.5	38.7
	M	A	L	Y	L	Q	N	C	Y	S	C	25.9	16.1
	M	A	L	Y	L	M	N	S	Y	S	A	25.7	36.5
	M	A	L	Y	L	Q	N	S	Y	S	V	25.6	45.6
	M	A	L	Y	L	M	N	S	Y	S	V	25.4	41.1

Supplementary Figure 23. Selection of PylRS variants for BocAhx 12 by tRNA display.

a, Chemical structure of BocAhx **12**. **b,** Spindle plot resulting from the tRNA display selection as described in **Supplementary Figure 22**. The selectivity is defined as the ratio of the relative abundance of a particular sequence in the positive samples (+ **12**), divided by the relative abundance in the control sample (-ncAA). The enrichment is defined as the ratio of the same sequence in the positive samples, divided by the relative abundance in the input library. The boxed region indicates sequences with selectivities of ≥ 5, and enrichments of ≥ 5. Black dots are sequences observed in all positive samples and all control samples. Green dots are sequences observed in all positive samples, and at least one control sample. Red dots are sequences only observed in the positive samples. **c**, Top 25 sequences, ordered by selectivity, resulting from the selection for substrate **12** with selectivities of ≥ 5, and enrichments of ≥ 5. **d**, Fluoro-tREX for PylRS variants **12**_1 to **12**_4 as well as wt PylRS. Experiments were performed using tRNA extracted from cells harboring a pMB1 plasmid encoding each *Mm*PylRS and *Mm*tRNA^{Pyl} in presence and absence of 4 mM **12**. Control samples for wt PylRS were performed in the presence and absence of 4 mM **12** and 2 mM **1**. Experiments were performed in triplicate.

a

13

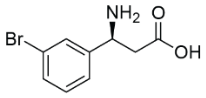

b

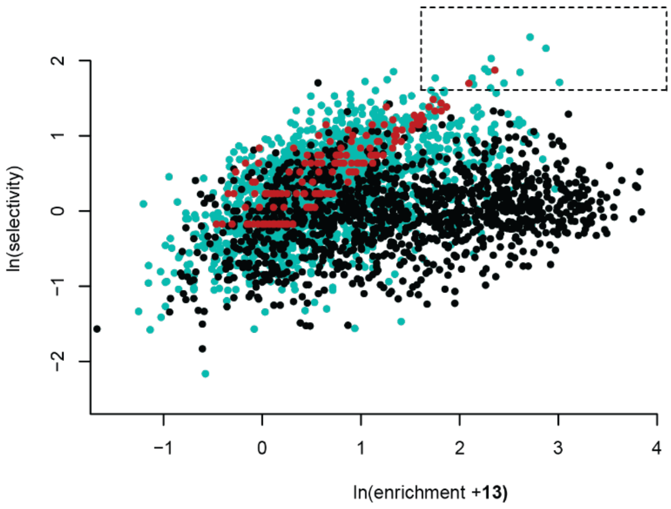

c

	M300	A302	L305	Y306	L309	M344	N346	C348	Y384	S399	V401	selectivity	enrichment
13_3 –	D	H	H	Y	L	M	G	F	Y	S	C	10.1	15.1
13_4 –	D	H	L	L	L	M	G	L	Y	S	S	8.7	17.7
13_1 –	D	H	L	L	L	M	G	L	Y	S	L	7.6	10.2
13_5 –	D	H	L	C	L	M	A	F	Y	S	C	6.6	9.5
13_2 –	D	C	C	F	V	M	G	L	Y	S	C	6.5	10.6
13_6 –	D	H	C	F	C	M	G	I	Y	S	A	6.4	9.9
	M	H	I	L	H	M	V	S	Y	S	V	6.3	13.6
	C	N	N	H	H	Q	V	G	Y	S	K	5.9	5.8
	D	H	H	Y	L	M	T	L	Y	S	K	5.9	8.4
	D	C	C	F	V	M	G	L	Y	S	V	5.5	20.3
	S	H	L	Y	L	M	A	C	Y	S	V	5.5	11.6
	D	H	L	Y	L	M	G	C	Y	S	V	5.5	8.1
	M	H	L	I	L	M	T	N	Y	S	K	5.4	8.5
	M	A	L	Y	L	Q	T	L	Y	S	C	5.1	10.1

d

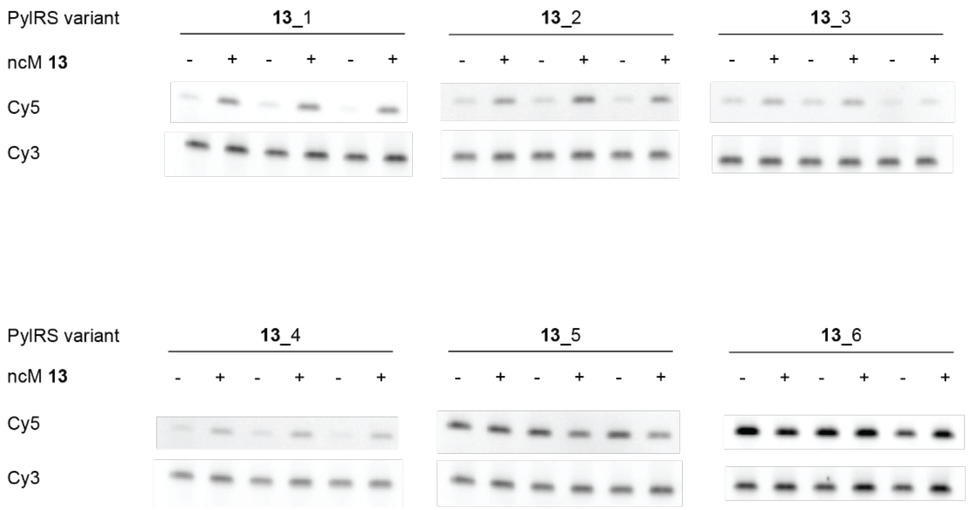

Supplementary Figure 24. Selection of PylRS variants for (*S*)-3-amino-3-(3-bromophenyl)propanoic acid ((*S*)β[3]mBrF) 13 by tRNA display.

a, Chemical structure of (*S*)β[3]mBrF **13**. **b,** Spindle plot resulting from the tRNA display selection as described in **Supplementary Figure 22**. Selectivity is defined as the ratio of the relative abundance of

a particular sequence in the positive samples (+ **12**), divided by the relative abundance in the control sample (-ncAA). The enrichment is defined as the ratio of the same sequence in the positive samples, divided by the relative abundance in the input library. The boxed region indicates sequences with selectivities of ≥ 5, and enrichments of ≥ 5. Black dots are sequences observed in all positive samples and all control samples. Green dots are sequences observed in all positive samples, and at least one control sample. Red dots are sequences only observed in the positive samples. **c**, The 14 sequences, ordered by selectivity, resulting from the selection for substrate **13** with selectivities of ≥ 5, and enrichments of ≥ 5. **d**, Fluoro-tREX for PylRS variants **13**_1 to **13**_6. Experiments were performed using tRNA extracted from cells harboring a pMB1 plasmid encoding each *Mm*PylRS and *Mm*$tRNA^{Pyl}$ in presence and absence of 4 mM **13**. Experiments were performed in triplicate.

a

b

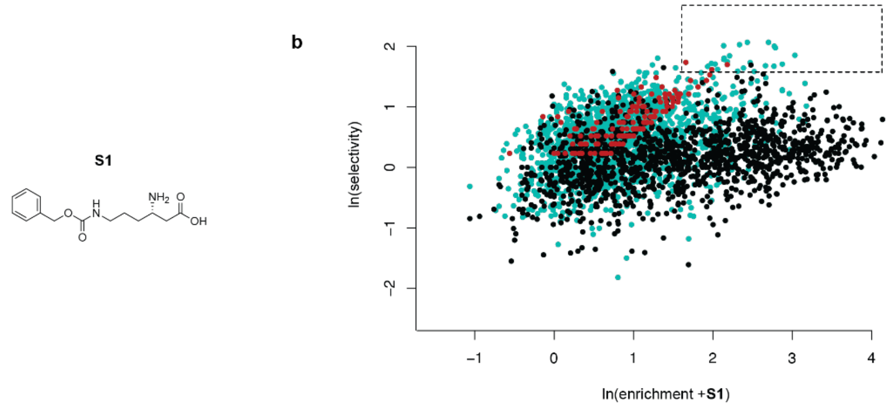

c

	M300	A302	L305	Y306	L309	M344	N346	C348	Y384	S399	V401	selectivity	enrichment
S1_1 –	M	A	L	Y	L	M	T	Y	Y	S	K	7.9	14.9
S1_2 –	D	C	C	F	L	M	G	I	Y	S	V	7.9	11.4
S1_3 –	E	H	L	Y	L	Q	A	L	Y	S	V	7.6	10.3
	C	H	L	Y	L	Q	S	L	Y	S	V	7.5	8.5
S1_4 –	C	H	H	Y	L	M	S	L	Y	S	V	7.4	16.0
S1_5 –	M	H	F	L	L	Q	A	V	Y	S	V	7.2	16.2
S1_6 –	M	H	F	F	L	Q	A	V	Y	S	V	6.4	20.8
	C	H	H	L	L	M	S	V	Y	S	C	6.3	9.1
	D	H	L	F	L	M	A	L	Y	S	K	6.1	8.5
	M	Y	C	F	F	Q	V	G	Y	S	T	6.0	8.5
	S	H	F	Y	L	M	S	L	Y	S	V	5.7	8.9
	S	C	C	F	F	Q	E	F	Y	S	C	5.7	5.3
	M	H	L	C	L	Q	C	V	Y	S	V	5.6	11.8
	M	H	H	L	L	M	E	F	Y	S	C	5.6	7.3
	S	H	L	H	L	M	A	C	Y	S	V	5.5	7.6
	M	H	L	I	L	M	G	V	Y	S	V	5.5	17.7
	M	H	L	R	H	Q	C	V	Y	S	V	5.5	8.9
	M	H	L	H	L	Q	V	L	Y	S	V	5.4	11.9
	C	H	H	Y	L	M	T	L	Y	S	K	5.4	14.3
	D	H	L	Y	L	M	V	F	Y	S	K	5.3	15.9
	M	H	H	L	L	M	T	L	Y	S	C	5.3	6.9
	S	H	L	L	L	M	V	L	Y	S	K	5.2	10.9
	S	C	C	F	L	M	A	S	Y	S	V	5.0	7.8
	M	H	L	L	L	Q	S	H	Y	S	T	5.0	7.3

d

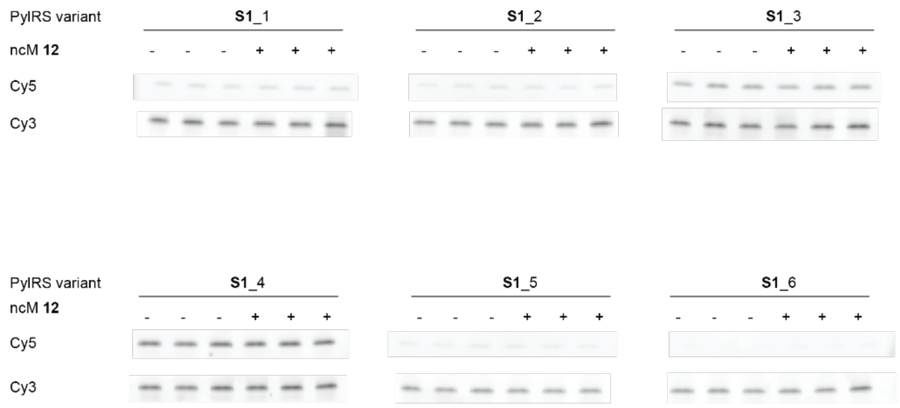

Supplementary Figure 25. Selection of PylRS variants for (*S*)-3-amino-6-(((benzyloxy)carbonyl)amino)hexanoic acid ((*S*)-β[3]CbzK) S1 by tRNA display.

a, Chemical structure of (*S*)-β[3]CbzK **S1**. **b**, Spindle plot resulting from the tRNA display selection as described in **Supplementary Figure 22**. The selectivity is defined as the ratio of the relative abundance of a particular sequence in the positive samples (+ **S1**), divided by the relative abundance in the control sample (-ncAA). The enrichment is defined as the ratio of the same sequence in the positive samples, divided by the relative abundance in the input library. The boxed region indicates sequences with

selectivities of ≥ 5, and enrichments of ≥ 5. Black dots are sequences observed in all positive samples and all control samples. Green dots are sequences observed in all positive samples, and at least one control sample. Red dots are sequences only observed in the positive samples. **c**, Top 25 sequences, ordered by selectivity, resulting from the selection for substrate **S1** with selectivities of ≥ 5, and enrichments of ≥ 5. **d**, Fluoro-tREX for PylRS variants **S1**_1 to **S1**_6. Experiments were performed using tRNA extracted from cells harboring a pMB1 plasmid encoding each *Mm*PylRS and *Mm*tRNA^{Pyl} in presence and absence of 4 mM **S1**. Experiments were performed in triplicate.

a

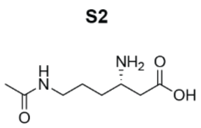

b

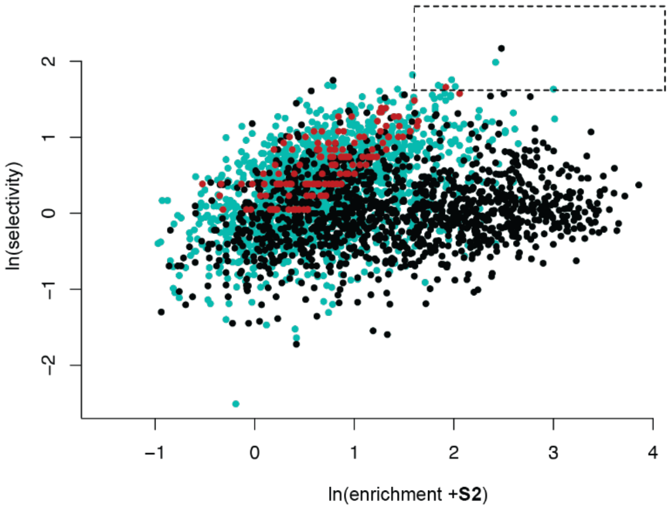

c

M300	A302	L305	Y306	L309	M344	N346	C348	Y384	S399	V401	selectivity	enrichment
C	H	L	I	L	Q	A	V	Y	S	V	8.8	11.9
D	H	L	N	L	M	S	I	Y	S	V	7.3	11.2
M	H	L	F	L	M	A	L	Y	S	V	5.8	7.2
C	N	N	D	H	Q	A	H	Y	S	L	5.4	6.3
S	H	L	Y	L	M	V	G	Y	S	L	5.3	7.5
C	N	N	N	N	M	N	S	Y	S	K	5.3	6.5
M	A	L	Y	L	M	N	C	Y	S	K	5.3	6.8
N	H	C	F	D	Q	C	V	Y	S	T	5.1	20.1

Supplementary Figure 26. Selection of PylRS variants for (*S*)-6-acetamido-3-aminohexanoic acid ((*S*)-β[3]AcK) S2 by tRNA display.

a, Chemical structure of (*S*)-β[3]Ac **S2**. **b,** Spindle plot resulting from the tRNA display selection as described in **Supplementary Figure 22**. The selectivity is defined as the ratio of the relative abundance of a particular sequence in the positive samples (+ **S2**), divided by the relative abundance in the control sample (-ncAA). The enrichment is defined as the ratio of the same sequence in the positive samples, divided by the relative abundance in the input library. The boxed region indicates sequences with selectivities of ≥ 5, and enrichments of ≥ 5. Black dots are sequences observed in all positive samples and all control samples. Green dots are sequences observed in all positive samples, and at least one control sample. Red dots are sequences only observed in the positive samples. **c**, The 8 sequences, ordered by selectivity, resulting from the selection for substrate **S2** with selectivities of ≥ 5, and enrichments of ≥ 5.

a

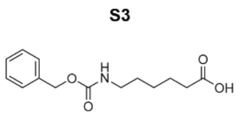

b

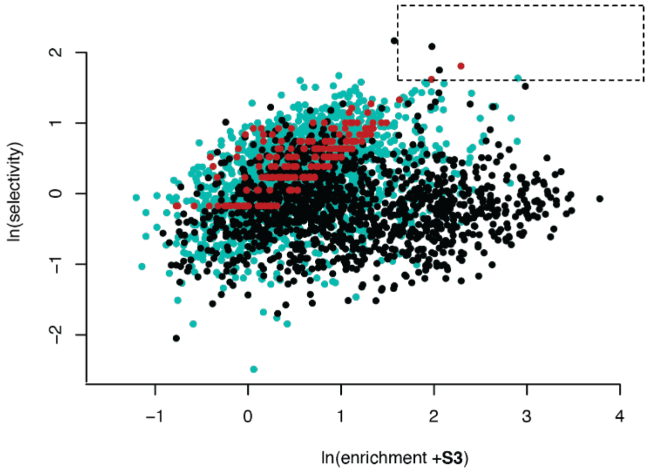

c

	M300	A302	L305	Y306	L309	M344	N346	C348	Y384	S399	V401	selectivity	enrichment
S3_1 –	M	A	L	Y	L	Q	N	G	Y	S	V	8.0	7.2
	M	H	H	Y	L	M	T	I	Y	S	K	6.1	9.9
	D	H	L	F	L	M	G	L	Y	S	C	5.8	7.8
	D	H	L	C	L	M	A	F	Y	S	K	5.1	18.2
	A	N	N	N	N	M	V	H	Y	S	A	5.0	7.2

d

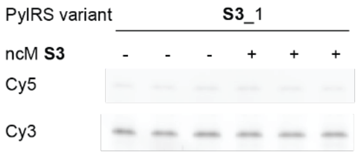

Supplementary Figure 27. Selection of PylRS variants for 6-(((benzyloxy)carbonyl)amino)hexanoic acid (CbzAhx) S3 by tRNA display.

a, Chemical structure of CbzAhx **S3**. **b,** Spindle plot resulting from the tRNA display selection as described in **Supplementary Figure 22**. The selectivity is defined as the ratio of the relative abundance of a particular sequence in the positive samples (+ **S3**), divided by the relative abundance in the control sample (-ncAA). The enrichment is defined as the ratio of the same sequence in the positive samples, divided by the relative abundance in the input library. The boxed region indicates sequences with selectivities of ≥ 5, and enrichments of ≥ 5. Black dots are sequences observed in all positive samples and all control samples. Green dots are sequences observed in all positive samples, and at least one control sample. Red dots are sequences only observed in the positive samples. **c**, The 5 sequences, ordered by selectivity, resulting from the selection for substrate **S3** with selectivities of ≥ 5, and enrichments of ≥ 5. **d**, Fluoro-tREX for PylRS variant **S3**_1. Experiments were performed using tRNA extracted from cells harboring a pMB1 plasmid encoding each *Mm*PylRS and *Mm*tRNA^{Pyl} in presence and absence of 4 mM **S3**. Experiments were performed in triplicate.

a

S4

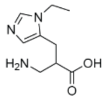

b

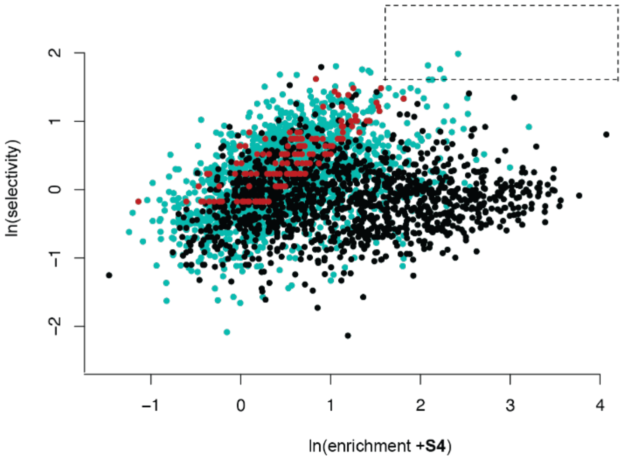

c

M300	A302	L305	Y306	L309	M344	N346	C348	Y384	S399	V401	selectivity	enrichment
C	H	H	Y	L	M	S	F	Y	S	V	7.3	11.2
C	H	L	L	L	M	C	N	Y	S	C	6.1	8.0
S	H	L	Y	L	M	V	S	Y	S	L	5.8	9.2
M	C	F	F	F	M	C	Y	Y	S	F	5.1	9.6

Supplementary Figure 28. Selection of PylRS variants for 3-amino-2-((1-ethyl-1*H*-imidazol-5-yl)methyl)propanoic acid (β²NeH) S4 by tRNA display.

a, Chemical structure of β²NeH **S4**. **b,** Spindle plot resulting from the tRNA display selection as described in **Supplementary Figure 22**. The selectivity is defined as the ratio of the relative abundance of a particular sequence in the positive samples (+ **S4**), divided by the relative abundance in the control sample (-ncAA). The enrichment is defined as the ratio of the same sequence in the positive samples, divided by the relative abundance in the input library. The boxed region indicates sequences with selectivities of ≥ 5, and enrichments of ≥ 5. Black dots are sequences observed in all positive samples and all control samples. Green dots are sequences observed in all positive samples, and at least one control sample. Red dots are sequences only observed in the positive samples. **c**, The 4 sequences, ordered by selectivity, resulting from the selection for substrate **S4** with selectivities of ≥ 5, and enrichments of ≥ 5.

a

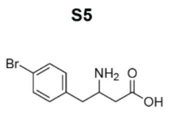

b

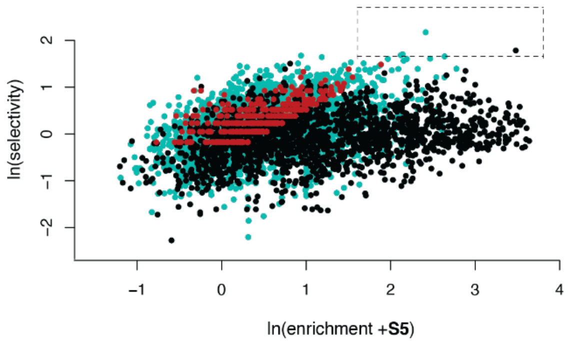

c

	M300	A302	L305	Y306	L309	M344	N346	C348	Y384	S399	V401	selectivity	enrichment
S5_1 –	M	H	F	L	L	M	C	V	Y	S	V	8.8	11.2
S5_2 –	M	H	I	L	L	M	T	S	Y	S	V	5.9	32.5
	D	H	L	Y	L	M	A	S	Y	S	V	5.5	8.5
	D	H	L	L	L	M	G	V	Y	S	V	5.4	8.3
	C	H	H	Y	L	M	T	L	Y	S	K	5.2	14.0

d

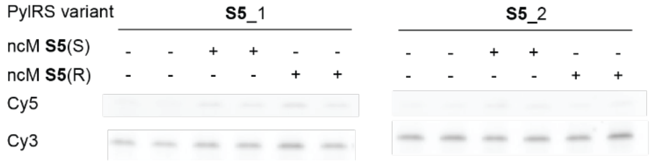

Supplementary Figure 29. Selection of PylRS variants for 3-amino-4-(4-bromophenyl)butanoic acid (β^{3}pBrhF) S5 by tRNA display.

a, Chemical structure of β^{3}pBrhF **S5**. **b,** Spindle plot resulting from the tRNA display selection as described in **Supplementary Figure 22**. The selectivity is defined as the ratio of the relative abundance of a particular sequence in the positive samples (+ **S5**), divided by the relative abundance in the control sample (-ncAA). The enrichment is defined as the ratio of the same sequence in the positive samples, divided by the relative abundance in the input library. The boxed region indicates sequences with selectivities of ≥ 5, and enrichments of ≥ 5. Black dots are sequences observed in all positive samples and all control samples. Green dots are sequences observed in all positive samples, and at least one control sample. Red dots are sequences only observed in the positive samples. **c**, The 5 sequences, ordered by selectivity, resulting from the selection for substrate **S5** with selectivities of ≥ 5, and enrichments of ≥ 5. **d**, Fluoro-tREX for PylRS variants **S5**_1 to **S5**_2. Experiments were performed using tRNA extracted from cells harboring a pMB1 plasmid encoding each *Mm*PylRS and *Mm*tRNA^{Pyl} in presence and absence of 2 mM of either the S or R enantiomer of **S5**. Experiments were performed in duplicates.

a b

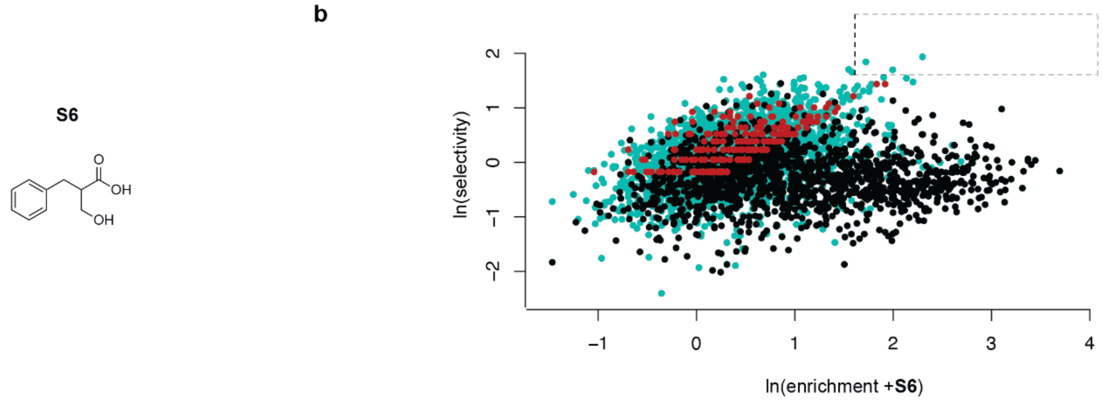

c

M300	A302	L305	Y306	L309	M344	N346	C348	Y384	S399	V401	selectivity	enrichment
M	N	N	N	N	Q	V	C	Y	S	F	6.9	9.9
M	G	S	G	D	Q	V	C	Y	S	L	6.3	5.6
M	H	L	C	L	Q	T	L	Y	S	K	5.5	7.3

Supplementary Figure 30. Selection of PylRS variants for 2-benzyl-3-hydroxypropanoic acid (β^2OH-F) S5. by tRNA display.

a, Chemical structure of β^2OH-F **S6**. **b,** Spindle plot resulting from the tRNA display selection as described in **Supplementary Figure 22**. The selectivity is defined as the ratio of the relative abundance of a particular sequence in the positive samples (+ **S6**), divided by the relative abundance in the control sample (-ncAA). The enrichment is defined as the ratio of the same sequence in the positive samples, divided by the relative abundance in the input library. The boxed region indicates sequences with selectivities of ≥ 5, and enrichments of ≥ 5. Black dots are sequences observed in all positive samples and all control samples. Green dots are sequences observed in all positive samples, and at least one control sample. Red dots are sequences only observed in the positive samples. **c**, The 3 sequences, ordered by selectivity, resulting from the selection for substrate **S6** with selectivities of ≥ 5, and enrichments of ≥ 5.

a

S7

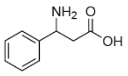

b

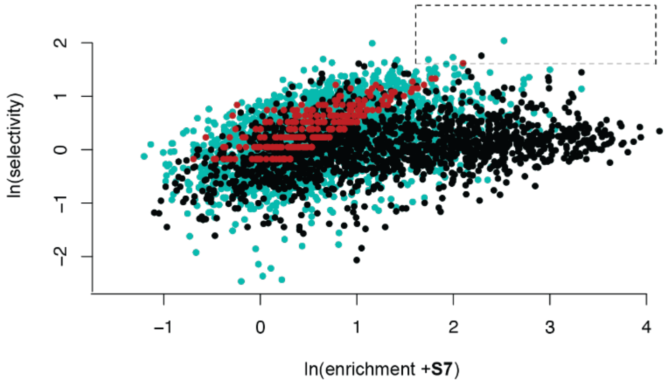

c

M300	A302	L305	Y306	L309	M344	N346	C348	Y384	S399	V401	selectivity	enrichment
S	H	L	Y	L	M	S	C	Y	S	L	7.7	12.5
M	H	H	Y	L	M	C	L	Y	S	L	5.8	9.9
M	H	L	L	L	Q	A	D	Y	S	S	5.6	7.4
C	H	L	L	L	M	G	I	Y	S	A	5.4	5.7
D	D	L	R	L	M	A	V	Y	S	V	5.0	8.2

Supplementary Figure 31. Selection of PylRS variants for 3-amino-3-phenylpropanoic acid (β^3F) S7 by tRNA display.

a, Chemical structure of β^3F **S7**. **b,** Spindle plot resulting from the tRNA display selection as described in **Supplementary Figure 22**. The selectivity is defined as the ratio of the relative abundance of a particular sequence in the positive samples (+ **S7**), divided by the relative abundance in the control sample (-ncAA). The enrichment is defined as the ratio of the same sequence in the positive samples, divided by the relative abundance in the input library. The boxed region indicates sequences with selectivities of ≥ 5, and enrichments of ≥ 5. Black dots are sequences observed in all positive samples and all control samples. Green dots are sequences observed in all positive samples, and at least one control sample. Red dots are sequences only observed in the positive samples. **c**, The 5 sequences, ordered by selectivity, resulting from the selection for substrate **S7** with selectivities of ≥ 5, and enrichments of ≥ 5.

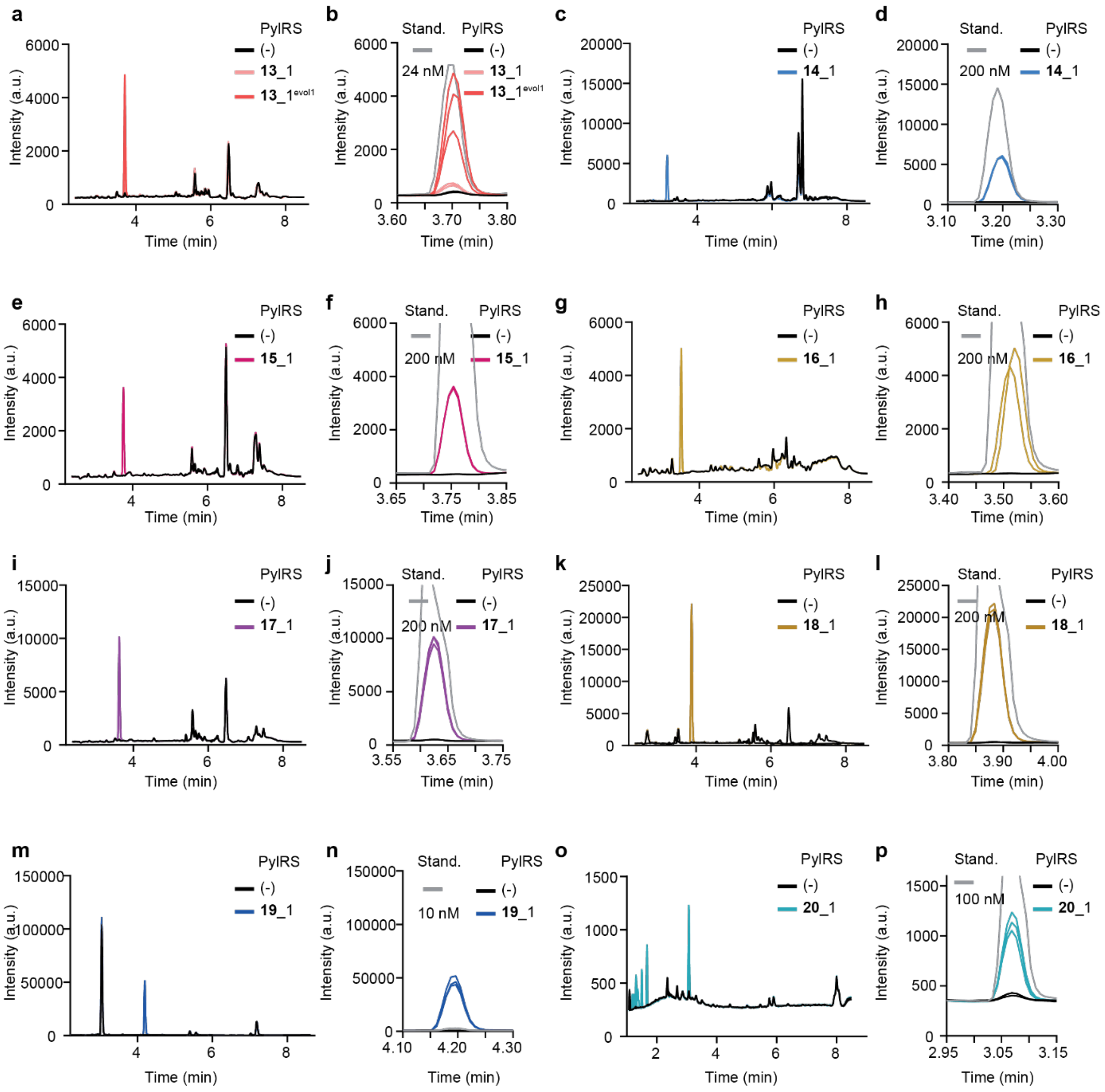

Supplementary Figure 32. LC-MS assay detecting 6-aminoquinolyl-*N*-hydroxysccinimidyl carbamate (AQC) derivatised ncMs 13-20 eluted from acylated *Mm*tRNA^{Pyl} in PylRS variant depending manner (see Extended Data Fig. 5 for schematic of the assay).

a, Full LC-MS spectrum of data presented in **Fig. 5c**. An authentic, derivatised standard of ncM **13** is shown in gray. The experiments were carried out in triplicates with similar results. **b,** Zoomed LC-MS spectrum shown in **a**. **c,** Full LC-MS spectrum of data presented in **Fig. 5e**. An authentic, derivatised standard of ncM **14** is shown in gray. The experiments were carried out in two replicates with similar results. **d,** Zoomed LC-MS spectrum shown in **c**. **d,** Full LC-MS spectrum of data presented in **Fig. 5g**. An authentic, derivatised standard of ncM **15** is shown in gray. The experiments were carried out in

doublicates with similar results. **e,** Zoomed LC-MS spectrum shown in **d**. **f,** Full LC-MS spectrum of data presented in **Fig. 5i**. An authentic, derivatised standard of ncM **16** is shown in gray. The experiments were carried out in two replicates with similar results. **g,** Zoomed LC-MS spectrum shown in **f**. **h,** Full LC-MS spectrum of data presented in **Fig. 5k**. An authentic, derivatised standard of ncM **17** is shown in gray. The experiments were carried out in triplicates with similar results. **i,** Zoomed LC-MS spectrum shown in **h**. **j,** Full LC-MS spectrum of data presented in **Fig. 5m**. An authentic, derivatised standard of ncM **18** is shown in gray. The experiments were carried out in triplicates with similar results. **k,** Zoomed LC-MS spectrum shown in **j**. **l,** Full LC-MS spectrum of data presented in **Fig. 5o**. An authentic, derivatised standard of ncM **19** is shown in gray. The experiments were carried out in triplicates with similar results. **m,** Zoomed LC-MS spectrum shown in **l**. **n,** Full LC-MS spectrum of data presented in **Fig. 5q**. An authentic, derivatised standard of ncM **20** is shown in gray. The experiments were carried out in triplicates with similar results.

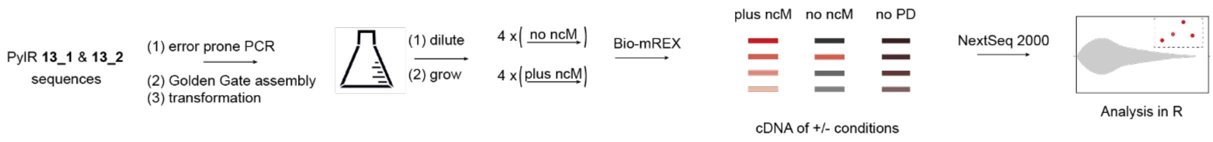

Supplementary Figure 33. Schematic representation of selection strategy for random mutagenesis and selections by tRNA display.

We performed an error prone PCR reaction across the active site sequence of PylRS variants **13_1** and **13_2** using the GeneMorph II (*Agilent*) kit. The diversified PCR amplicons were cloned into a fresh ColE1 plasmid backbone by Golden Gate assembly and the error prone stmRNA libraries transformed into BL21 cells and grown overnight. Cells were grown to OD_{600} of 0.3-0.4. 2.6 mL of the library culture was added into a stock solution of **13** to a concentration of 4 mM. 2.6 mL of the library culture was also added to a well without ncM. The cells were grown for 40 min, stmRNAs induced, and cells grown for another 20 min and the RNA was isolated. Bio-mREX was performed on the isolated RNA for each sample. The experiment was performed in four replicates, leading to eight cDNA samples. An additional four cDNA samples were generated for four of the RNA inputs to Bio-mREX. The resulting twelve cDNA samples were sequenced by NGS and analyzed to generate spindle plots and sequence tables.

a

	M300	A302	L305	Y306	L309	M344	N346	C348	Y384	S399	V401
13_1 –	D	H	L	L	L	M	G	L	Y	S	L
13_2 –	D	C	C	F	V	M	G	L	Y	S	C

b

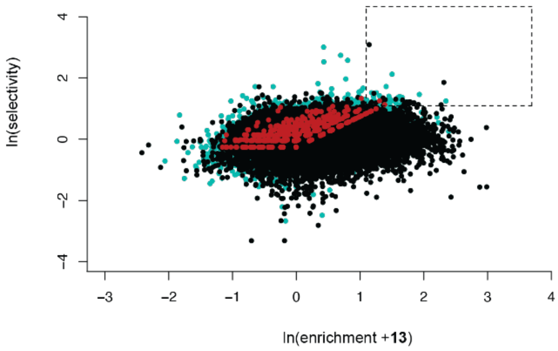

c

	mutation 1	mutation 2	selectivity	enrichment
13_1_evol4	F289V		21.9	3.1
13_1_evol1	N307K	F366L	8.3	4.5
13_1_evol5	T364A	T387I	5.6	4.1
13_1_evol6	P319A	T387I	3.4	5.9
13_1_evol3	G421A		3.4	3.3
13_1_evol2	F295I	T387I	3.3	6.1
13_1_evol7	T387S	D414V	3.3	6.3
13_1_evol8	F295L	K375R	3.1	9.7

d

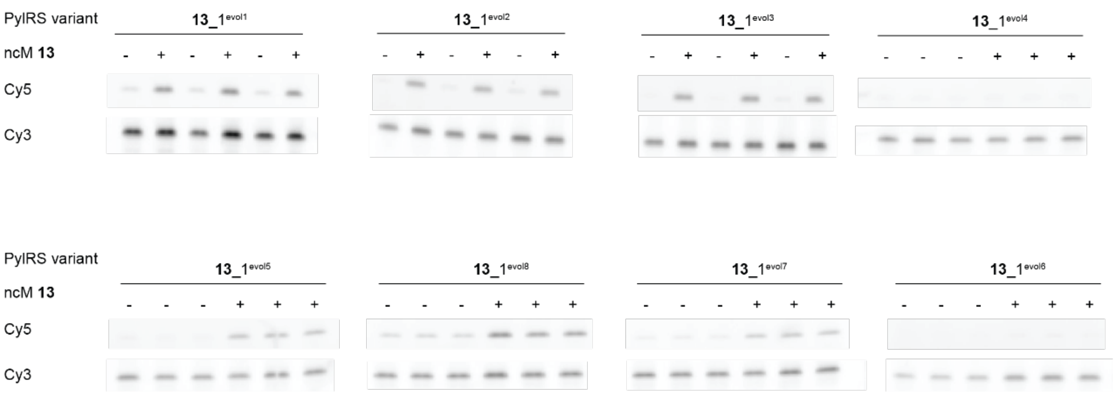

Supplementary Figure 34. Selection of improved PylRS variants for ((*S*)β^{3}mBrF) 13 using random mutagenesis libraries by tRNA display.

a, Parental active site sequences of (*S*)β^{3}mBrF PylRS **13**_1 and **13**_2 used as templates for random mutageneis. **b,** Spindle plot resulting from the tRNA display selection as described in **Supplementary Figure 33**. The selectivity is defined as the ratio of the relative abundance of a particular sequence in the positive samples (+ **13**), divided by the relative abundance in the control sample (-ncAA). The enrichment is defined as the ratio of the same sequence in the positive samples, divided by the relative abundance in the input library. The boxed region indicates sequences with selectivities of ≥ 3, and enrichments of ≥ 3. Black dots are sequences observed in all positive samples and all control samples. Green dots are sequences observed in all positive samples, and at least one control sample. Red dots are sequences only observed in the positive samples. Spindle plot resulting from the tRNA display

selection as described in **Supplementary Figure 33**. The selectivity is defined as the ratio of the relative abundance of a particular sequence in the positive samples (+ **13**), divided by the relative abundance in the control sample (-ncAA). The enrichment is defined as the ratio of the same sequence in the positive samples, divided by the relative abundance in the input library. The boxed region indicates sequences with selectivities of ≥ 3, and enrichments of ≥ 3. Black dots are sequences observed in all positive samples and all control samples. Green dots are sequences observed in all positive samples, and at least one control sample. Red dots are sequences only observed in the positive samples. **c**, Table with non-programmed mutations of evolved PylRS variants ordered by the calculated selectivity from the NGS analysis. We chracterised the most selective, the highest enriched, as well as the most abundant variants from the sequences with a selectivities of ≥ 3, and enrichments of ≥ 3. All sequences were base on the parental PylRS variant **13**_1. **d**, Fluoro-tREX data of PylRS variants **13**_1^{evol} to **13**_1^{evol8}. tRNAs were isolated from DH10β cell harboring a pMB1 plasmid encoding each *Mm*PylRS and *Mm*$tRNA^{Pyl}$ in presence and absence of 4 mM **13**. We performed fluoro-tREX on the isolated tRNAs. All experiments were caried out in triplicates with similar results.

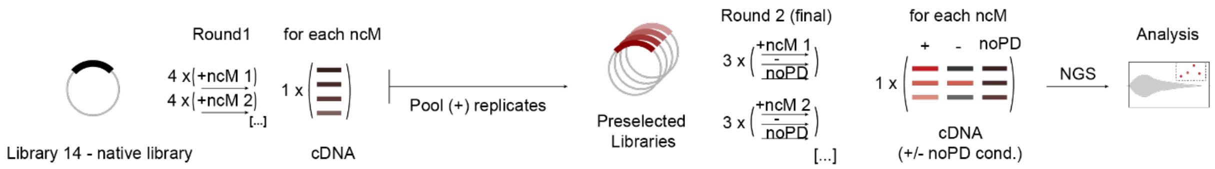

Supplementary Figure 35. Schematic representation of two step tRNA display selection strategy for non-canonical monomers.

Library 14 was transformed into BL21 cells and grown overnight. Cells were grown to OD_{600} of 0.3-0.4. 4 mL of the library culture was added into stock solutions of each ncM. The cells were grown for 40 min, stmRNAs induced, and cells grown for another 20 min and the RNA was isolated. Bio-mREX was performed on the isolated RNA for each sample. The experiment was performed in four replicates. For each replicate the cDNA was amplified with primers suitable for Golden Gate assembly. Then all amplicons of the libraries selected for the same ncM were combined at equimolar ratios and cloned into a fresh ColE1vector backbone. This created one preselected library of each ncM. The pre-selected libraries were transformed into BL21 cells and grown over night.

Cells were grown to OD_{600} of 0.3-0.4. For each ncM, 4 mL of the respective preselected library culture was added into stock solutions of the ncM. For each preselected library 4mL of the library culture was also added to a well without ncM. The cells were grown for 40 min, stmRNAs induced, and cells grown for another 20 min and the RNA was isolated. Bio-mREX was performed on the isolated RNA for each sample. The experiment was performed in three replicates, leading to 6 cDNA samples per ncM. An additional three cDNA samples were generated for each ncM using three RNA inputs to bio-mREX of the respective preselected libraries. For each ncM the resulting nine cDNA samples were sequenced by NGS and analyzed to generate spindle plots and sequence tables.

a

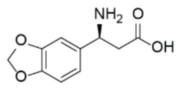

b

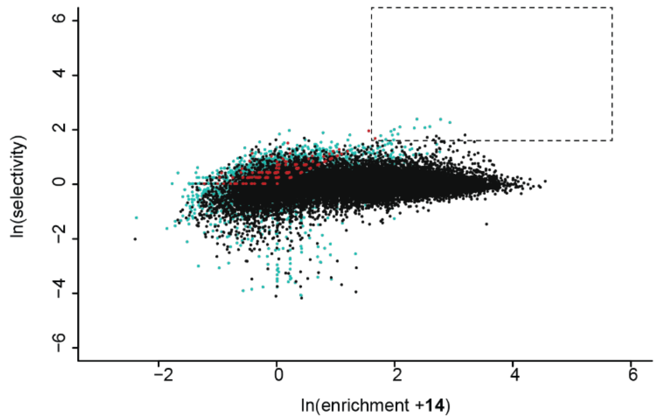

c

	M300	A302	L305	Y306	L309	M344	N346	C348	Y384	S399	V401	selectivity	enrichment
	C	H	G	Y	L	M	S	Y	Y	S	V	10.9	10.8
14_2 –	D	H	L	Y	H	Q	G	S	Y	S	V	10.8	16.1
14_3 –	D	H	I	Y	C	Q	G	V	Y	S	V	9.7	18.8
14_4 –	D	H	L	Y	L	E	A	G	Y	S	V	8.3	13.4
14_5 –	D	H	L	H	N	M	A	S	Y	S	V	8.2	8.1
14_6 –	D	H	L	L	N	M	G	S	Y	S	V	8.0	12.3
14_7 –	D	H	C	Y	F	M	G	G	Y	S	V	7.9	10.3
	M	H	N	Y	L	M	T	I	Y	S	K	7.4	7.3
14_8 –	D	H	L	Y	H	M	A	S	Y	S	C	7.4	7.3
	D	H	I	L	L	M	V	V	Y	S	A	7.0	6.9
	A	A	L	Y	L	M	A	M	Y	S	V	6.3	7.8
14_9 –	D	H	L	I	H	M	A	S	Y	S	V	6.1	24.3
	D	H	L	Y	S	Q	G	L	Y	S	V	6.1	7.1
	D	H	N	Y	N	M	G	S	Y	S	V	6.0	5.8
14_10 –	D	H	I	Y	L	Q	G	V	Y	S	L	5.9	14.9
	D	H	L	I	L	M	G	S	Y	S	V	5.6	12.8
	D	H	L	Y	L	M	C	S	Y	S	L	5.4	15.6
14_11 –	D	H	L	Y	H	Q	A	S	Y	S	V	5.4	5.3
	D	C	I	L	L	M	A	V	Y	S	V	5.4	5.3
	D	H	I	Y	F	M	S	G	Y	S	V	5.2	6.4
	D	H	H	Y	L	M	V	S	Y	S	V	5.1	7.3
14_1 –	D	H	L	Y	L	Q	G	N	Y	S	V	5.1	14.2
14_12 –	D	H	C	Y	L	Q	A	S	Y	S	V	5.1	21.8

d

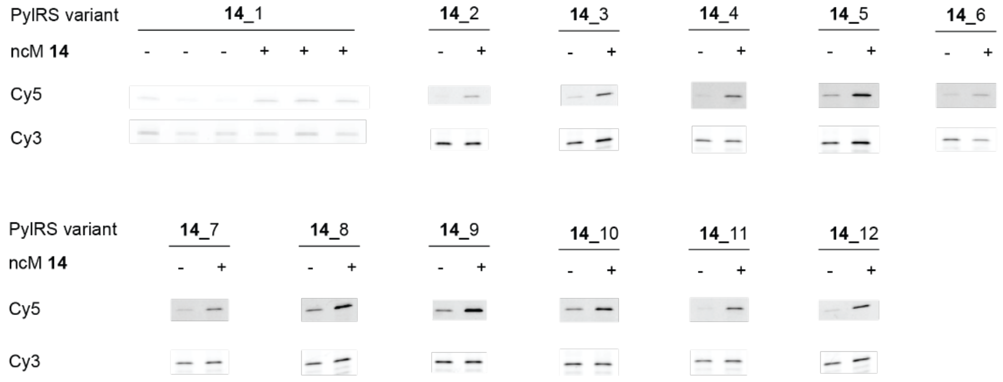

Supplementary Figure 36. election of PylRS variants for (*S*)-3-amino-3-(benzo[*d*][1,3]dioxol-5-yl)propanoic acid ((*S*)β^{3}MDF) (14) by tRNA display.

a, Chemical structure of (*S*)β^{3}MDF **14**. **b,** Spindle plot resulting from the tRNA display selection as described in **Supplementary Figure 35**. The selectivity is defined as the ratio of the relative abundance of a particular sequence in the positive samples (+ **14**), divided by the relative abundance in the control

sample (-ncAA). The enrichment is defined as the ratio of the same sequence in the positive samples, divided by the relative abundance in the input library. The boxed region indicates sequences with selectivities of ≥ 5, and enrichments of ≥ 5. Black dots are sequences observed in all positive samples and all control samples. Green dots are sequences observed in all positive samples, and at least one control sample. Red dots are sequences only observed in the positive samples. **c**, The 25 sequences, ordered by selectivity, resulting from the selection for substrate **14** with selectivities of ≥ 5, and enrichments of ≥ 5. **d**, Fluoro-tREX for PylRS variants **14**_1 to **14**_12. Experiments were performed using tRNA extracted from cells harboring a pMB1 plasmid encoding each *Mm*PylRS and *Mm*tRNA^{Pyl} in presence and absence of 4 mM **14**. Experiments for **14**_1 were performed in triplicates.

a

15

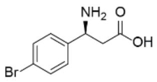

b

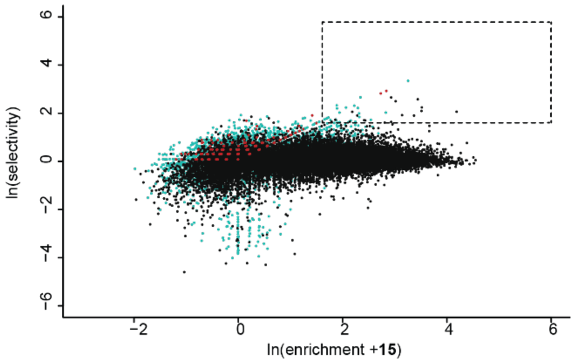

c

	M300	A302	L305	Y306	L309	M344	N346	C348	Y384	S399	V401	selectivity	enrichment
15_2 –	D	H	F	Y	F	Q	A	G	Y	S	V	28.4	25.9
15_1 –	D	H	L	I	F	M	G	G	Y	S	V	18.7	17.1
15_3 –	D	H	I	Y	N	M	G	C	Y	S	L	16.8	15.4
15_4 –	D	H	N	Y	N	Q	A	S	Y	S	V	14.3	10.4
15_5 –	D	H	I	Y	N	Q	A	C	Y	S	V	14.2	18.7
15_6 –	D	H	L	Y	F	Q	G	G	Y	S	V	13.2	31.2
15_7 –	D	H	I	Y	N	Q	G	S	Y	S	V	12.1	20.4
15_8 –	D	H	F	Y	F	Q	A	G	Y	S	C	10.2	6.6
	D	H	L	Y	I	M	G	C	Y	S	L	10.1	9.2
15_9 –	D	H	F	Y	F	M	A	G	Y	S	L	9.4	33.8
15_10 –	D	H	N	Y	N	Q	G	S	Y	S	V	9.3	7.9
	D	H	I	Y	S	M	A	C	Y	S	L	9.2	9.9
	S	G	L	Y	Y	M	A	G	Y	S	V	9.0	6.6
15_11 –	D	H	L	Y	L	Q	G	G	Y	S	V	8.1	33.5
	D	H	N	Y	N	M	A	S	Y	S	V	7.9	65.8
15_12 –	D	H	I	Y	C	Q	G	I	Y	S	V	7.8	8.9
	D	H	L	C	G	M	A	F	Y	S	V	7.6	7.0
	D	H	L	Y	N	Q	G	S	Y	S	V	7.6	16.9
	D	H	I	Y	G	Q	G	F	Y	S	V	7.3	10.1
	D	H	V	Y	L	Q	G	C	Y	S	L	6.8	23.1
	D	H	L	Y	L	Q	G	C	Y	S	L	6.7	9.8
	D	H	V	Y	I	Q	A	C	Y	S	V	6.7	29.0
	D	H	F	Y	F	M	G	G	Y	S	A	6.3	10.2
	D	H	I	Y	S	M	G	H	Y	S	L	6.2	5.6
	D	H	I	Y	I	M	S	C	Y	S	L	5.9	5.4
	C	H	L	Y	F	M	T	I	Y	S	A	5.7	5.3
	M	H	L	C	L	Q	C	C	Y	S	K	5.7	5.2
	S	H	C	Y	L	M	V	N	Y	S	V	5.7	6.5
	D	H	L	Y	V	M	A	N	Y	S	V	5.6	6.4

d

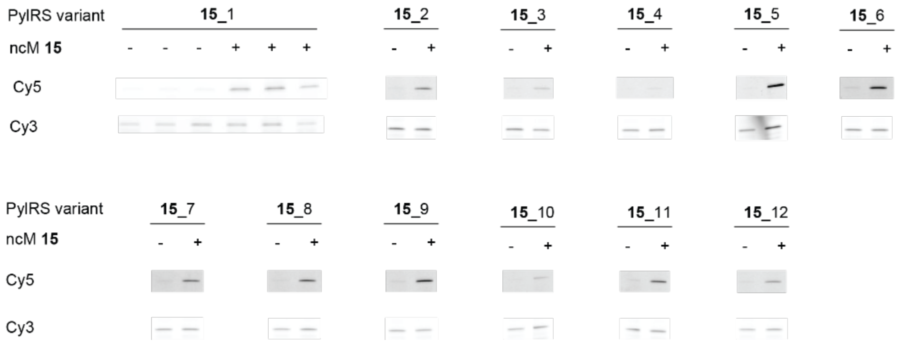

Supplementary Figure 37. Selection of PylRS variants for (*S*)-3-amino-3-(4-bromophenyl)propanoic acid ((*S*)β^{3}pBrF) (15) by tRNA display.

a, Chemical structure of (*S*)β^{3}pBrF **15**. **b,** Spindle plot resulting from the tRNA display selection as described in **Supplementary Figure 35**. The selectivity is defined as the ratio of the relative abundance of a particular sequence in the positive samples (+ **15**), divided by the relative abundance in the control sample (-ncAA). The enrichment is defined as the ratio of the same sequence in the positive samples,

divided by the relative abundance in the input library. The boxed region indicates sequences with selectivities of ≥ 5, and enrichments of ≥ 5. Black dots are sequences observed in all positive samples and all control samples. Green dots are sequences observed in all positive samples, and at least one control sample. Red dots are sequences only observed in the positive samples. **c**, The 25 sequences, ordered by selectivity, resulting from the selection for substrate **15** with selectivities of ≥ 5, and enrichments of ≥ 5. **d**, Fluoro-tREX for PylRS variants **15**_1 to **15**_12. Experiments were performed using tRNA extracted from cells harboring a pMB1 plasmid encoding each *Mm*PylRS and *Mm*$tRNA^{Pyl}$ in presence and absence of 4 mM **15**. Experiments for **15**_1 were performed in triplicates.

a

16

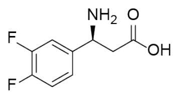

b

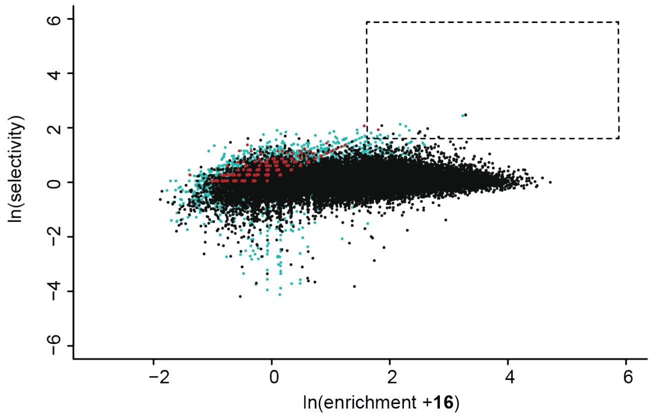

c

	M300	A302	L305	Y306	L309	M344	N346	C348	Y384	S399	V401	selectivity	enrichment
**16_2** –	D	H	L	Y	G	Q	G	F	Y	S	V	12.1	26.7
**16_3** –	D	H	I	L	L	Q	G	S	Y	S	V	11.8	26.7
**16_1** –	D	H	V	Y	L	Q	G	V	Y	S	L	11.5	25.4
**16_4** –	D	H	L	Y	N	Q	G	S	Y	S	V	8.4	8.8
**16_5** –	D	H	L	L	H	Q	G	S	Y	S	V	8.0	6.4
**16_6** –	D	N	L	Y	L	Q	G	A	Y	S	V	7.9	9.7
**16_7** –	D	H	C	Y	V	Q	G	L	Y	S	V	7.2	14.0
**16_8** –	D	H	L	I	L	Q	G	L	Y	S	V	6.9	6.1
**16_9** –	D	H	I	Y	V	M	G	L	Y	S	C	6.9	6.1
	D	H	L	R	H	M	G	C	Y	S	V	6.7	10.3
	D	H	I	L	H	M	G	S	Y	S	V	6.5	12.3
**16_10** –	D	H	L	Y	S	Q	G	F	Y	S	V	6.3	12.3
	A	H	L	Y	L	M	T	L	X	S	K	6.1	5.4
**16_11** –	D	H	L	Y	L	Q	G	S	Y	S	L	6.0	12.0
	D	L	L	Y	L	M	G	T	Y	S	V	5.9	7.8
**16_12** –	D	H	L	Y	G	M	G	F	Y	S	F	5.7	6.3
	D	H	I	Y	L	M	A	F	Y	S	T	5.6	8.7
	D	D	L	L	C	M	G	R	Y	S	V	5.5	13.4
	D	H	C	Y	G	M	G	L	Y	S	V	5.4	7.2
	C	H	H	Y	C	M	S	I	Y	S	C	5.4	6.0
	D	H	I	I	C	M	G	H	Y	S	V	5.3	16.9
	M	H	F	L	L	M	A	V	Y	S	C	5.1	6.8
	D	H	I	F	S	M	A	S	Y	S	V	5.1	5.6
	M	H	I	I	L	M	C	V	Y	S	K	5.1	6.7

d

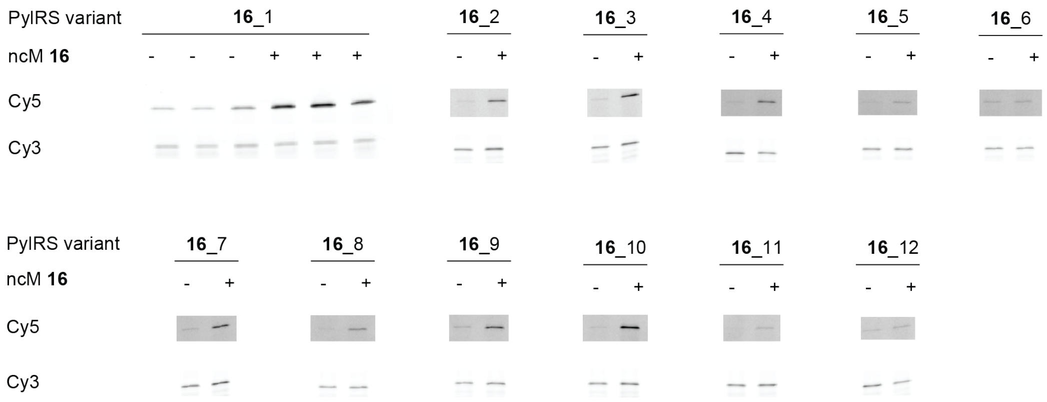

Supplementary Figure 38. Selection of PylRS variants for (*S*)-3-amino-3-(3,4-difluorophenyl)propanoic acid ((*S*)β^3pmFF) (16) by tRNA display.

a, Chemical structure of (*S*)β^3pmFF **16**. **b,** Spindle plot resulting from the tRNA display selection as described in **Supplementary Figure 35**. The selectivity is defined as the ratio of the relative abundance of a particular sequence in the positive samples (+ **16**), divided by the relative abundance in the control

sample (-ncAA). The enrichment is defined as the ratio of the same sequence in the positive samples, divided by the relative abundance in the input library. The boxed region indicates sequences with selectivities of ≥ 5, and enrichments of ≥ 5. Black dots are sequences observed in all positive samples and all control samples. Green dots are sequences observed in all positive samples, and at least one control sample. Red dots are sequences only observed in the positive samples. **c**, The 25 sequences, ordered by selectivity, resulting from the selection for substrate **16** with selectivities of ≥ 5, and enrichments of ≥ 5. **d**, Fluoro-tREX for PylRS variants **16**_1 to **16**_12. Experiments were performed using tRNA extracted from cells harboring a pMB1 plasmid encoding each *Mm*PylRS and *Mm*tRNA^{Pyl} in presence and absence of 4 mM **16**. Experiments for **16**_1 were performed in triplicates.

a

17

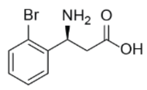

b

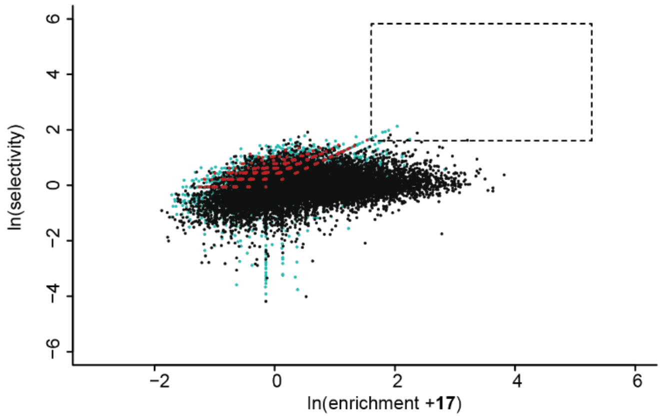

c

hit name	M300	A302	L305	Y306	L309	M344	N346	C348	Y384	S399	V401	selectivity	enrichment
	D	H	L	Y	H	Q	G	S	Y	S	V	8.4	7.7
17_2 –	D	H	L	I	L	Q	G	V	Y	S	K	7.3	6.7
	D	H	L	Y	C	M	A	C	Y	S	A	6.9	6.3
17_3 –	D	H	L	L	H	Q	G	S	Y	S	C	6.8	6.0
17_1 –	D	D	L	R	L	Q	G	Y	Y	S	V	6.6	10.7
17_4 –	D	H	L	L	H	M	G	S	Y	S	K	6.2	6.9
17_5 –	D	H	L	Y	L	Q	T	C	Y	S	A	5.9	5.4
17_6 –	D	H	L	H	L	Q	G	C	Y	S	V	5.6	5.1
17_7 –	M	H	L	Y	G	Q	S	I	Y	S	V	5.5	6.7
	S	H	L	Y	L	G	A	C	Y	S	V	5.3	9.5
	D	H	L	Y	L	Q	G	G	Y	S	C	5.1	9.2
	D	H	L	Y	L	Q	G	V	Y	S	L	5.1	24.7

d

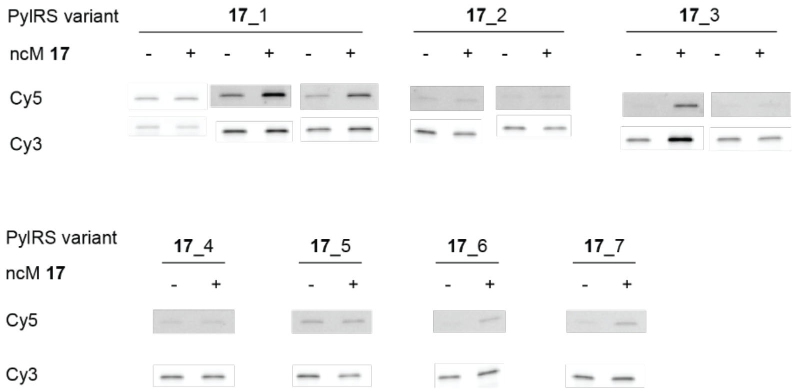

Supplementary Figure 39. Selection of PylRS variants for (*S*)-3-amino-3-(2-bromophenyl)propanoic acid ((*S*)β^{3}oBrF) (17) by tRNA display.

a, Chemical structure of (*S*)β^{3}oBrF **17**. **b,** Spindle plot resulting from the tRNA display selection as described in **Supplementary Figure 35**. The selectivity is defined as the ratio of the relative abundance of a particular sequence in the positive samples (+ **17**), divided by the relative abundance in the control sample (-ncAA). The enrichment is defined as the ratio of the same sequence in the positive samples,

divided by the relative abundance in the input library. The boxed region indicates sequences with selectivities of ≥ 5, and enrichments of ≥ 5. Black dots are sequences observed in all positive samples and all control samples. Green dots are sequences observed in all positive samples, and at least one control sample. Red dots are sequences only observed in the positive samples. **c**, The 12 sequences, ordered by selectivity, resulting from the selection for substrate **17** with selectivities of ≥ 5, and enrichments of ≥ 5. **d**, Fluoro-tREX for PylRS variants **17**_1 to **17**_7. Experiments were performed using tRNA extracted from cells harboring a pMB1 plasmid encoding each *Mm*PylRS and *Mm*tRNA^{Pyl} in presence and absence of 4 mM **17**. Experiments for **17**_1 were performed in triplicates.

a

18

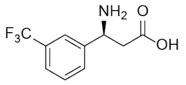

b

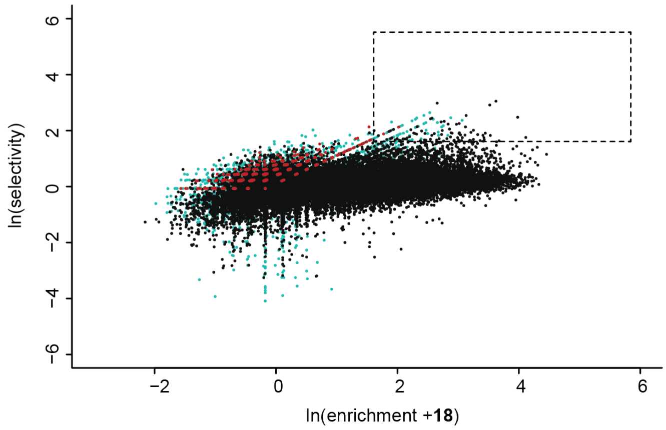

c

hit name	M300	A302	L305	Y306	L309	M344	N346	C348	Y384	S399	V401	selectivity	enrichment
	D	H	V	Y	C	M	G	F	Y	S	T	21.1	37.5
18_2 –	D	H	I	F	C	M	G	F	Y	S	V	19.7	14.2
18_1 –	D	H	F	Y	N	M	G	H	Y	S	V	18.4	33.8
18_3 –	D	Y	V	L	L	M	G	V	Y	S	V	14.0	12.6
18_4 –	D	Y	V	F	C	M	G	V	Y	S	S	12.7	11.4
	D	H	V	V	G	M	G	F	Y	S	V	12.7	22.9
18_5 –	D	H	V	F	L	Q	G	V	Y	S	V	11.9	53.6
18_6 –	D	H	V	Y	C	M	G	V	Y	S	T	11.7	17.8
18_7 –	D	H	I	L	H	M	G	S	Y	S	C	11.6	10.2
18_8 –	D	H	L	F	V	M	G	F	Y	S	V	11.5	13.0
	D	H	F	L	L	M	G	C	Y	S	C	11.2	12.7
	D	H	L	Y	N	Q	G	C	Y	S	V	11.1	13.3
	D	H	L	Y	H	M	G	C	Y	S	V	11.1	9.9
	D	H	I	R	L	M	G	V	Y	S	V	10.7	21.5
	D	Y	L	I	G	M	G	L	Y	S	V	10.5	9.4
	D	H	F	N	V	M	G	Y	Y	S	K	10.3	16.0
	D	H	V	F	L	M	G	I	Y	S	L	10.1	15.2
	D	N	I	Y	L	M	G	L	Y	S	V	10.1	6.7
	D	H	L	R	H	M	G	L	Y	S	V	9.8	14.3
	D	Y	I	F	L	M	G	C	Y	S	C	9.8	11.8
	D	H	V	L	L	M	G	S	Y	S	C	9.5	20.4
	D	H	I	V	L	M	G	V	Y	S	V	9.4	11.4
	D	H	V	L	L	M	G	C	Y	S	V	9.3	17.0
	D	H	I	L	L	M	G	V	Y	S	C	9.3	19.6
	D	H	V	F	C	M	G	L	Y	S	V	8.9	8.0

d

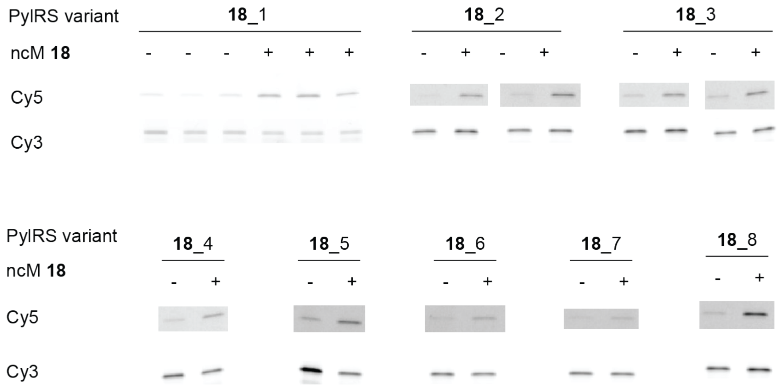

Supplementary Figure 40. Selection of PylRS variants for (*S*)-3-amino-3-(3-(trifluoromethyl)phenyl)propanoic acid (18) ((*S*)β^{3}mCF$_3$F) by tRNA display.

a, Chemical structure of (*S*)β^{3}mCF$_3$F **18**. **b,** Spindle plot resulting from the tRNA display selection as described in **Supplementary Figure 35**. The selectivity is defined as the ratio of the relative abundance of a particular sequence in the positive samples (+ **18**), divided by the relative abundance in the control sample (-ncAA). The enrichment is defined as the ratio of the same sequence in the positive samples, divided by the relative abundance in the input library. The boxed region indicates sequences with selectivities of ≥ 5, and enrichments of ≥ 5. Black dots are sequences observed in all positive samples and all control samples. Green dots are sequences observed in all positive samples, and at least one control sample. Red dots are sequences only observed in the positive samples. **c**, The 25 sequences, ordered by selectivity, resulting from the selection for substrate **18** with selectivities of ≥ 5, and enrichments of ≥ 5. **d**, Fluoro-tREX for PylRS variants **18**_1 to **18**_8. Experiments were performed using tRNA extracted from cells harboring a pMB1 plasmid encoding each *Mm*PylRS and *Mm*tRNA^{Pyl} in presence and absence of 4 mM **18**. Experiments for **18**_1 were performed in triplicates.

a b

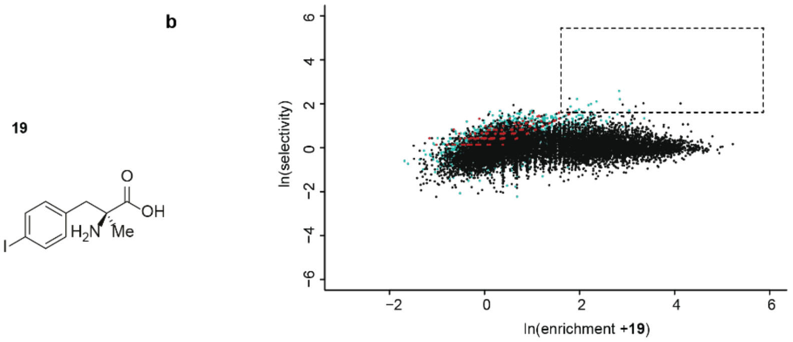

c

hit name	M300	A302	L305	Y306	L309	M344	N346	C348	Y384	S399	V401	selectivity	enrichment
19_2	D	H	S	Y	F	M	G	G	Y	S	V	13.2	17.0
19_3	D	S	L	N	G	M	A	F	Y	S	L	9.4	6.0
	D	C	I	F	C	M	V	C	Y	S	K	9.2	7.0
19_4	D	G	C	F	F	M	A	G	Y	S	L	9.1	17.2
19_5	D	G	C	F	F	M	A	G	Y	S	V	8.2	10.6
19_6	D	H	C	Y	L	M	A	G	Y	S	C	7.6	28.4
19_1	D	H	F	Y	L	M	C	C	Y	S	L	7.5	61.8
19_7	D	H	C	F	F	M	G	G	Y	S	V	7.3	9.4
19_8	D	H	C	Y	F	M	G	G	Y	S	V	7.1	18.3
19_9	D	G	C	F	F	M	G	G	Y	S	L	6.9	8.9
	T	G	L	Y	L	M	C	H	Y	S	V	6.6	24.1
	E	G	N	D	L	M	E	C	Y	S	A	6.5	6.1
	D	S	L	N	G	M	G	F	Y	S	L	5.7	5.7
	C	H	L	Y	L	H	T	L	Y	S	V	5.7	7.3
	M	H	L	Y	H	M	V	S	Y	S	C	5.6	12.1
	D	H	S	Y	F	M	A	G	Y	S	V	5.4	21.0
	S	H	F	Y	N	M	A	C	Y	S	L	5.4	6.9
	A	H	L	Y	L	Q	G	F	Y	S	V	5.2	6.7
	D	H	L	N	V	M	G	V	Y	S	V	5.2	9.4
	D	C	L	F	V	M	A	I	Y	S	V	5.1	8.1
	S	H	H	L	L	M	V	C	Y	S	V	5.1	7.7
	M	H	L	Y	L	H	A	I	Y	S	V	5.0	6.5

d

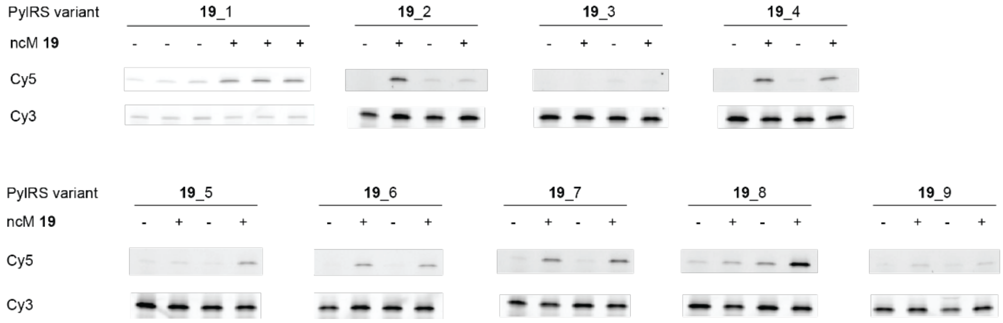

Supplementary Figure 41. Selection of PylRS variants for (*S*)-2-amino-3-(4-iodophenyl)-2-methylpropanoic acid (19) ((*S*)α-Me-pIF) by tRNA display.

a, Chemical structure of (*S*)α-Me-pIF **19**. **b,** Spindle plot resulting from the tRNA display selection as described in **Supplementary Figure 35**. The selectivity is defined as the ratio of the relative abundance of a particular sequence in the positive samples (+ **19**), divided by the relative abundance in the control sample (-ncAA). The enrichment is defined as the ratio of the same sequence in the positive samples, divided by the relative abundance in the input library. The boxed region indicates sequences with selectivities of ≥ 5, and enrichments of ≥ 5. Black dots are sequences observed in all positive samples

and all control samples. Green dots are sequences observed in all positive samples, and at least one control sample. Red dots are sequences only observed in the positive samples. **c**, The 25 sequences, ordered by selectivity, resulting from the selection for substrate **19** with selectivities of ≥ 5, and enrichments of ≥ 5. **d**, Fluoro-tREX for PylRS variants **19**_1 to **19**_9. Experiments were performed using tRNA extracted from cells harboring a pMB1 plasmid encoding each *Mm*PylRS and *Mm*tRNA^{Pyl} in presence and absence of 4 mM **19**. Experiments for **19**_1 were performed in triplicates.

a

20

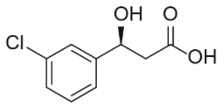

b

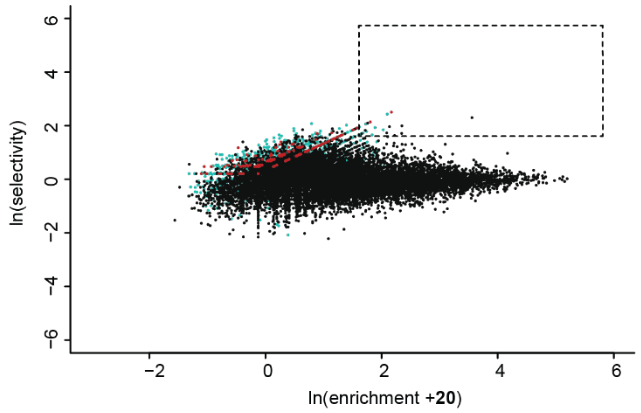

c

	M300	A302	L305	Y306	L309	M344	N346	C348	Y384	S399	V401	selectivity	enrichment
**20_2** –	N	H	L	N	L	M	G	L	Y	S	L	12.2	8.8
**20_3** –	N	H	L	Y	I	M	G	V	Y	S	C	11.4	8.2
**20_1** –	N	H	L	Y	L	M	G	I	Y	S	C	10.0	35.0
**20_4** –	S	H	L	Y	L	E	A	T	Y	S	V	8.1	5.8
**20_5** –	N	H	L	Y	L	Q	C	I	Y	S	K	7.3	10.5
**20_6** –	A	H	C	Y	L	Q	A	L	Y	S	V	7.1	8.5
**20_7** –	N	H	V	Y	S	M	G	I	Y	S	V	6.9	6.6
	D	G	S	D	D	Q	N	C	Y	S	S	6.2	5.6
	N	N	N	R	H	Q	V	C	Y	S	V	6.0	5.7
	S	D	N	S	L	Q	V	V	Y	S	S	5.6	5.4
	D	H	C	F	L	M	S	V	Y	S	A	5.5	10.4
	D	H	L	I	R	M	N	F	Y	S	T	5.5	5.2
	S	H	N	Y	L	M	A	I	Y	S	V	5.5	5.2
	M	H	C	Y	I	Q	E	V	Y	S	L	5.4	5.1

d

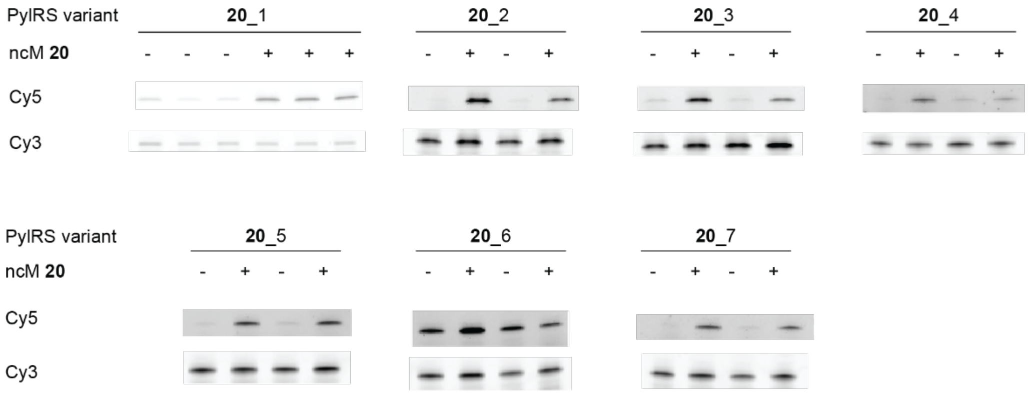

Supplementary Figure 42. Selection of PylRS variants for (*S*)-3-(3-chlorophenyl)-3-hydroxypropanoic acid (20) (OH-(*S*)β^3mClF) by tRNA display.

a, Chemical structure of OH-(*S*)β^3mClF **20**. **b,** Spindle plot resulting from the tRNA display selection as described in **Supplementary Figure 35**. The selectivity is defined as the ratio of the relative abundance of a particular sequence in the positive samples (+ **20**), divided by the relative abundance in the control sample (-ncAA). The enrichment is defined as the ratio of the same sequence in the positive

samples, divided by the relative abundance in the input library. The boxed region indicates sequences with selectivities of ≥ 5, and enrichments of ≥ 5. Black dots are sequences observed in all positive samples and all control samples. Green dots are sequences observed in all positive samples, and at least one control sample. Red dots are sequences only observed in the positive samples. **c**, The 14 sequences, ordered by selectivity, resulting from the selection for substrate **20** with selectivities of ≥ 5, and enrichments of ≥ 5. **d**, Fluoro-tREX for PylRS variants **20**_1 to **20**_7. Experiments were performed using tRNA extracted from cells harboring a pMB1 plasmid encoding each *Mm*PylRS and *Mm*$tRNA^{Pyl}$ in presence and absence of 4 mM **20**. Experiments for **20**_1 were performed in triplicates.

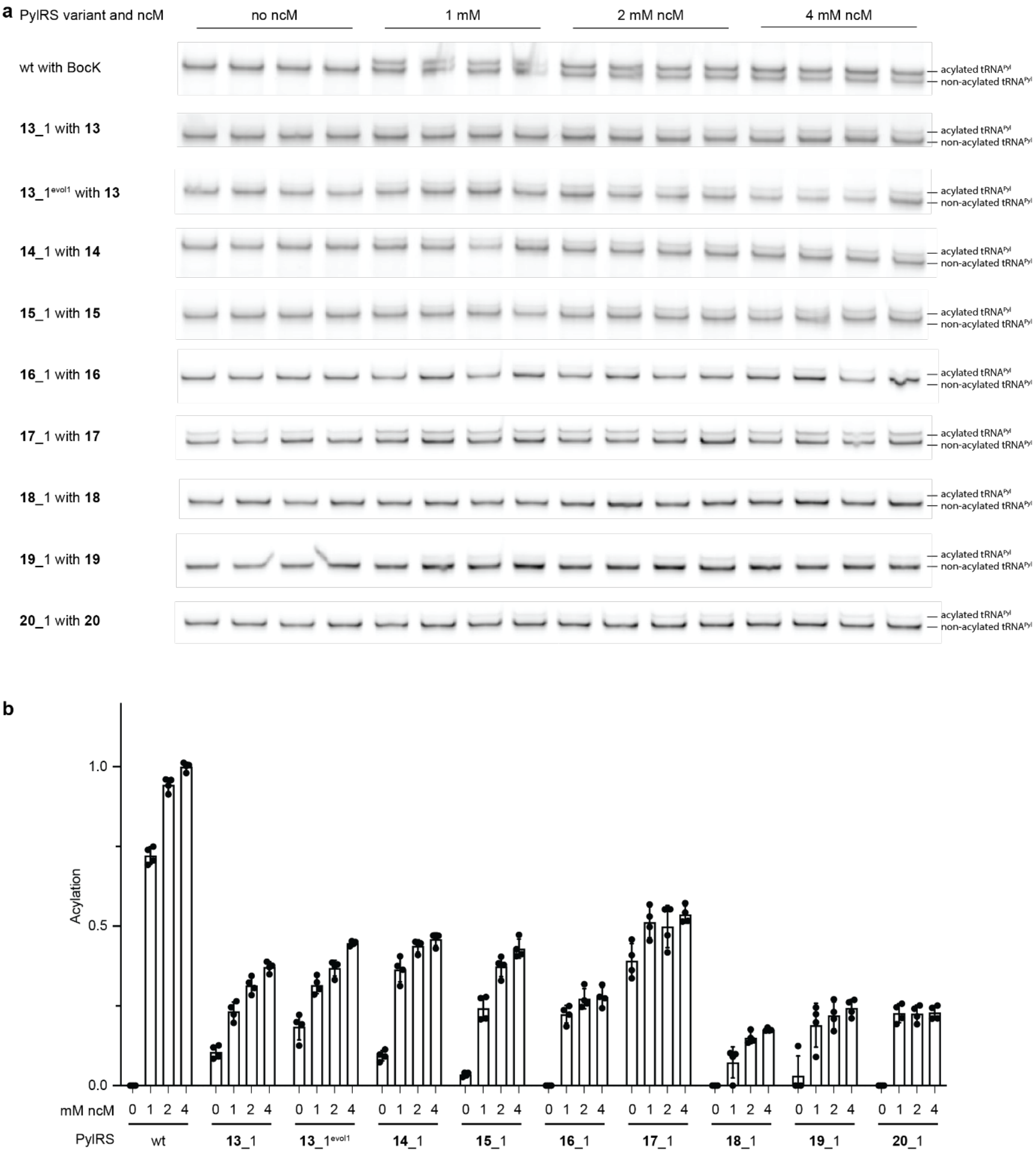

Supplementary Figure 43. Characterisation of *in vivo* acylation activity of evolved, ncM specific PylRS variants by tREX, we have previously shown this assay quantitatively reports on *in vivo* acylation.[1]

a, tREX for evolved PylRS variants. tREX was performed on *Mm*tRNA^{Pyl} extracted from cells harboring a pMB1 plasmid encoding each *Mm*PylRS variant and *Mm*tRNA^{Pyl} grown in the presence of 0, 1, 2 or 4 mM of the indicated ncM. Experiments were performed in four replicates. **b**, Quantification

of *in vivo* acylation activity of evolved PylRS variants. The acylation levels were quantified by taking the ratio of the upper band (acylated tRNA) divided by the sum of the signal intensity of the upper and lower bands (acylated and non-acylated tRNA) in the tREX gels shown in **a**. The data is shown as a fraction of the acylation activity of wt PylRS with 4 mM BocK (55 ± 1 %), which was set to 1. The dots represent the individual data points, the bars represent the mean and the error bars represent the s.d. All numerical values are given in **Supplementary Data 1**. We note that this assay is performed under conditions where the acyl-tRNA complexes are not actively consumed by translation in the cell and can accumulate over time. Under these conditions, enzymes with less activity in the cell might lead to comparable levels of measured acylation to enzymes with more activity in the cell. We note that **13**, **15**,**18**,**19** are ribosomally incorporated so must be acylated at a level to support ribosomal translation; this suggests the other monomers may well be acylated at a level that would support translation if they were efficient substrates for other parts of the translational machinery. In future work it may be useful to measure the time-dependent acylation; this is challenging to assess in vivo because the acylation of a tRNA in vivo is likely dominated by the rate of ncM uptake rather than the rate of acylation. Published in vitro acylation assays for kinetic measurements currently require access to either radioactive monomers or radiolabelled tRNAs; the reagents for generating these tRNAs are not currently available, purifying active soluble synthetase for in vitro measurements is challenging and tRNAs for in vitro measurements typically lack post-transcriptional modifications. As a result, in vitro measurements of acylation with this system may not provide insight into in vivo behaviour.

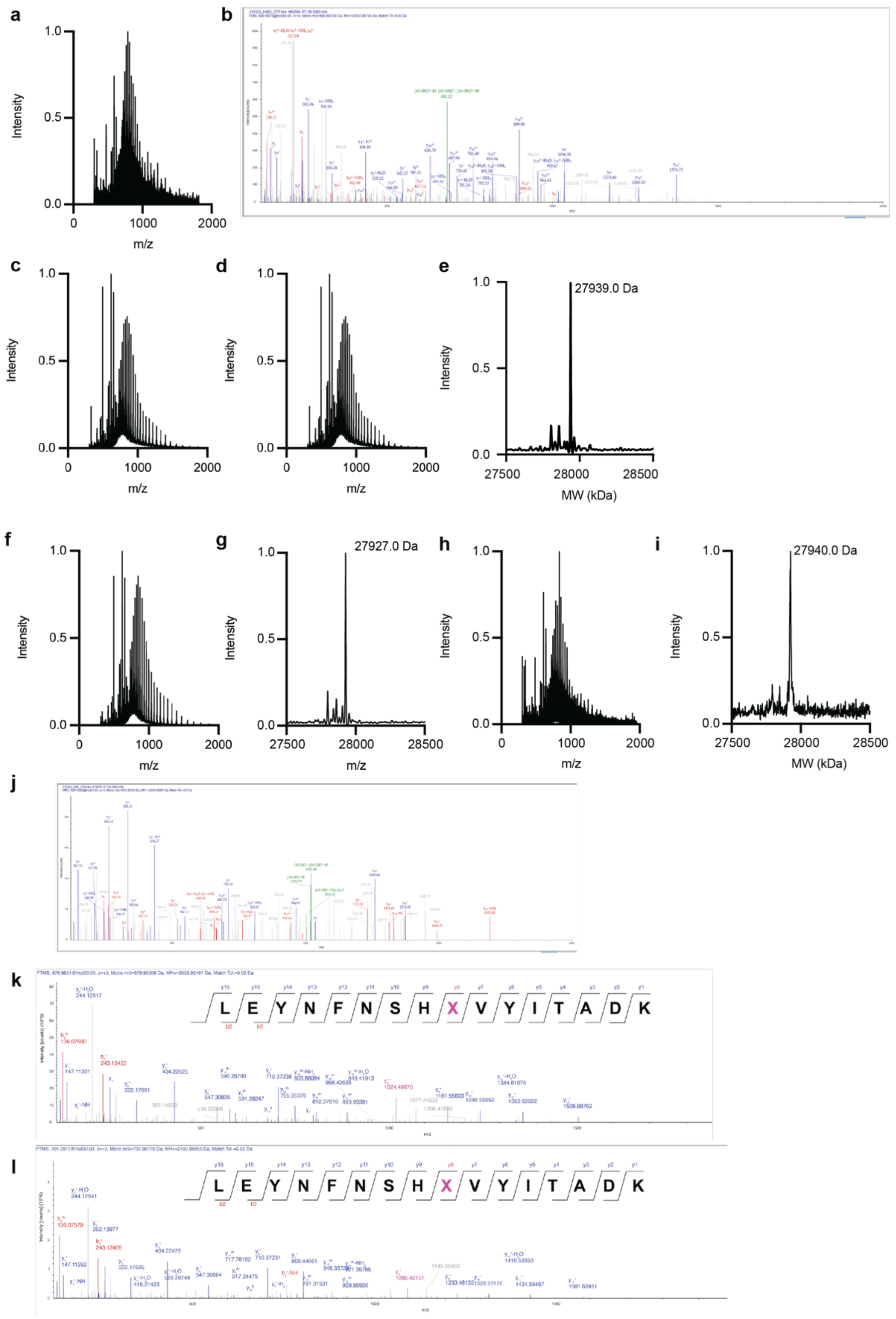

Supplementary Figure 44. Mass spectrometry of ncM containing GFP150X$_{His6}$.

a, Raw mass spectrum for purified GFP150(*S*)β^{3}mBrF$_{His6}$ produced from cells containing PylRS **13**_1^{evol1}, *Mm*tRNA$^{Pyl}_{CUA}$, GFP(150TAG)$_{His6}$ and 4 mM **13**. The deconvoluted mass spectrum is shown

in **Fig. 5s**. **b**, MS/MS spectra of ncAA-containing peptides obtained following tryptic digest of GFP150(S)β^{3}mBrF$_{His6}$ produced as described in **a**. The precursor ions confirm the incorporation of **13** at position 150 of GFP. Fragmentation of each peptide is predicted to yield a series of b ions (red) and a series of y ions (blue), as well as ions corresponding to the full-length peptide (green). **c**, Raw mass spectrum for purified GFP150(*S*)α-Me-pIF$_{His6}$ produced from cells containing PylRS **19**_1, *Mm*tRNA$^{Pyl}_{CUA}$, GFP(150TAG)$_{His6}$ and 4 mM **19**. The deconvoluted mass spectrum is shown in **Fig. 5t**. **d**, Raw mass spectrum for purified GFP150(*S*)β^{3}pBrF$_{His6}$ produced from cells containing PylRS **15**_1, *Mm*tRNA$^{Pyl}_{CUA}$, *GFP(150TAG)$_{His6}$* and 4 mM **15**. **e**, Intact ESI-MS of GFP(150(*S*)β^{3}pBrF)$_{His6}$ purified from cells harbouring PylRS(**15**_1), *Mm*tRNA$^{Pyl}_{CUA}$, and *GFP(150TAG)$_{His6}$* grown in the presence of 4 mM **15**. Found mass: 27,939.0 Da, predicted mass: 27,938.2 Da **f**, Raw mass spectrum for purified GFP150((*S*)β^{3}mCF$_3$F) $_{His6}$ produced with PylRS **18**_1. **g**, intact ESI-MS of GFP150((*S*)β^{3}mCF$_3$F)$_{His6}$ purified from cells harbouring PylRS(**18**_1), *Mm*tRNA$^{Pyl}_{CUA}$, and *GFP(150TAG)$_{His6}$* grown in the presence of 4 mM **18**. Found mass: 27,927.0 Da, predicted mass: 27,928.3 Da. **h**, Raw mass spectrum for purified GFP150(*S*)β^{3}mBrF$_{His6}$ produced with PylRS **13**_1. **i**, intact ESI-MS of GFP(150(*S*)β^{3}mBrF)$_{His6}$ purified from cells harbouring PylRS(**13**_1), *Mm*tRNA$^{Pyl}_{CUA}$, and *GFP(150TAG)$_{His6}$* grown in the presence of 4 mM **13**. Found mass: 27,940.0 Da, predicted mass: 27,938.2 Da. **j-l**, MS/MS spectra of ncAA-containing peptides obtained following tryptic digest of GFP150(S)β^{3}mBrF$_{His6}$ produced with PylRS **13**_1, GFP150(S)β^{3}mCF3F$_{His6}$ produced with PylRS **18**_1, or GFP150(*S*)α-Me-pIF$_{His6}$ produced with PylRS **19**_1, respectively. The precursor ions confirm the incorporation of **13**, **18**, or **19** at position 150 of GFP. Fragmentation of each peptide is predicted to yield a series of b ions (red) and a series of y ions (blue), as well as ions corresponding to the full-length peptide (green or pink for panels **j** and **l**).

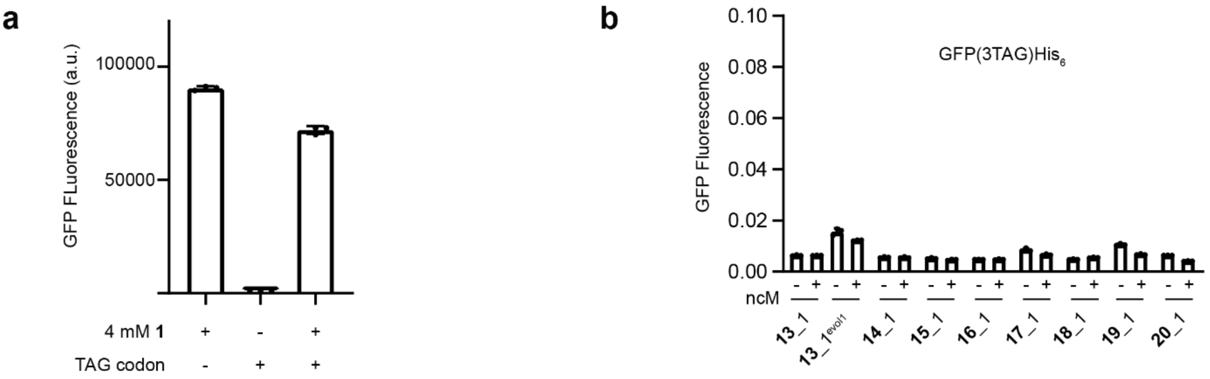

Supplementary Figure 45. GFP production.

a, Production of GFP_{His6} (or GFP_{His6} incorporating **1** at position 150) was measured by GFP fluorescence. Cell contained a pMB1 plasmid encoding wt PylRS/$tRNA^{Pyl}_{CUA}$ pair and a p15A plasmid encoding *$GFP3TAG_{His6}$* or *GFP_{His6}* in the presence and absence of 4 mM of BocK (**1**). The yield of GFP_{His6} incorporating **1**, produced from *$GFP3TAG_{His6}$*, wt PylRS/$tRNA^{Pyl}_{CUA}$ pair and **1**, was 80% of $wtGFP_{His6}$ protein, produced from *$wtGFP_{His6}$*. The fluorescence values shown are normalized by OD. The bars show the mean of three biological replicates, individual replicates are indicated by dots, error bars show ± s.d.. All numerical values are provided (**Supplementary Data 1**). The fluorescence-based yield of GFP_{His6} generated by each ncM incorporation is reported relative to the GFP_{His6} fluorescence generated by incorporation of **1** in **Fig. 5r**; the percentage yields are: 6.6% (for PylRS variant **13**_1 and ncM **13**), 14.4% (for PylRS variant **13**_1^{evol1} and ncM **13**), 3.7% (for PylRS variant **15**_1 and ncM **15**), 4.1% (for PylRS variant **18**_1 and ncM **18**), and 37.0% (for PylRS variant **19**_1 and ncM **19**). For reference, the Ni-NTA purified yield of GFP_{His6}, expressed from cells containing a pMB1 plasmid encoding wt PylRS/$tRNA^{Pyl}_{CUA}$ pair and a p15A plasmid encoding *$GFP3TAG_{His6}$* in the presence of 4 mM of BocK (**1**), was 105 ± 8 mg/l culture. The purified yields of the $GFP150X_{His6}$ with the relevant ncMs were: 8.4 ± 0.2 mg/l culture for PylRS13_1; 16.2 ± 1.2 mg/l culture for PylRS13_1^{evol}; 3.6 ± 0.4 mg/l culture for PylRS15_1; 3.1 ± 0.7 mg/l culture for PylRS18_1; 35.5 ± 0.9 mg/l culture for PylRS19_1. **b**, Attempt of protein production of GFP3$(\mathbf{13\ to\ 20})_{His6}$ from *$GFP3TAG_{His6}$* from cells harboring a pMB1 plasmid encoding the indicated PylRS variant/$tRNA^{Pyl}_{CUA}$ pair and a p15A plasmid encoding *$GFP3TAG_{His6}$* in the presence and absence of 4 mM of the respective ncM. Fluorescence is shown as a fraction of the fluorescence generated by the wt PylRS/$tRNA^{Pyl}_{CUA}$ pair with 4 mM BocK (**1**) and *GFP(3TAG)His_6*. The bars show the mean of three biological replicates, individual replicates

are indicated by dots, error bars show ± s.d.. All numerical values are provided (**Supplementary Data 1**).

Supplementary References

1 Cervettini, D. *et al.* Rapid discovery and evolution of orthogonal aminoacyl-tRNA synthetase–tRNA pairs. *Nat. Biotechnol.*, 1–11 (2020). https://doi.org:10.1038/s41587-020-0479-2

2 Spinck, M. *et al.* Genetically programmed cell-based synthesis of non-natural peptide and depsipeptide macrocycles. *Nature Chemistry* **15**, 61-69 (2023).

3 Kobayashi, T., Yanagisawa, T., Sakamoto, K. & Yokoyama, S. Recognition of non-alpha-amino substrates by pyrrolysyl-tRNA synthetase. *J Mol Biol* **385**, 1352-1360 (2009). https://doi.org:10.1016/j.jmb.2008.11.059

4 Pan, X., Wang, H., Li, C., Zhang, J. Z. H. & Ji, C. MolGpka: A Web Server for Small Molecule pKa Prediction Using a Graph-Convolutional Neural Network. *Journal of Chemical Information and Modeling* **61**, 3159-3165 (2021). https://doi.org:10.1021/acs.jcim.1c00075
